# Membrane-Derived Phospholipids Control Synaptic Neurotransmission and Plasticity

**DOI:** 10.1371/journal.pbio.1002153

**Published:** 2015-05-21

**Authors:** Victoria García-Morales, Fernando Montero, David González-Forero, Guillermo Rodríguez-Bey, Laura Gómez-Pérez, María Jesús Medialdea-Wandossell, Germán Domínguez-Vías, José Manuel García-Verdugo, Bernardo Moreno-López

**Affiliations:** 1 Grupo de Neurodegeneración y Neuroreparación (GRUNEDERE), Área de Fisiología, Facultad de Medicina, Universidad de Cádiz, Cádiz, Spain; 2 Salus Infirmorum, Universidad de Cádiz, Cádiz, Spain; 3 Centro de Investigación Príncipe Felipe, CIBERNED, Universidad de Valencia, Valencia, Spain; ICM—Institut du Cerveau et de la Moelle épinière Hôpital Pitié-Salpêtrière 47, bd de l'Hôpital, FRANCE

## Abstract

Synaptic communication is a dynamic process that is key to the regulation of neuronal excitability and information processing in the brain. To date, however, the molecular signals controlling synaptic dynamics have been poorly understood. Membrane-derived bioactive phospholipids are potential candidates to control short-term tuning of synaptic signaling, a plastic event essential for information processing at both the cellular and neuronal network levels in the brain. Here, we showed that phospholipids affect excitatory and inhibitory neurotransmission by different degrees, loci, and mechanisms of action. Signaling triggered by lysophosphatidic acid (LPA) evoked rapid and reversible depression of excitatory and inhibitory postsynaptic currents. At excitatory synapses, LPA-induced depression depended on LPA_1_/G_αi/o_-protein/phospholipase C/myosin light chain kinase cascade at the presynaptic site. LPA increased myosin light chain phosphorylation, which is known to trigger actomyosin contraction, and reduced the number of synaptic vesicles docked to active zones in excitatory boutons. At inhibitory synapses, postsynaptic LPA signaling led to dephosphorylation, and internalization of the GABA_Aγ2_ subunit through the LPA_1_/G_α12/13_-protein/RhoA/Rho kinase/calcineurin pathway. However, LPA-induced depression of GABAergic transmission was correlated with an endocytosis-independent reduction of GABA_A_ receptors, possibly by GABA_Aγ2_ dephosphorylation and subsequent increased lateral diffusion. Furthermore, endogenous LPA signaling, mainly via LPA_1_, mediated activity-dependent inhibitory depression in a model of experimental synaptic plasticity. Finally, LPA signaling, most likely restraining the excitatory drive incoming to motoneurons, regulated performance of motor output commands, a basic brain processing task. We propose that lysophospholipids serve as potential local messengers that tune synaptic strength to precedent activity of the neuron.

## Abstract 2

Lysophospholipids derived from membranes are important regulators of neurotransmission, acting as local messengers that couple synaptic strength to recent neuronal activity.

## Author Summary

Neuronal networks are modules of synaptic connectivity that underlie all brain functions, from simple reflexes to complex cognitive processes. Synaptic plasticity allows these networks to adapt to changing external and internal environments. Membrane-derived bioactive phospholipids are potential candidates to control short-term synaptic plasticity. We demonstrate that lysophosphatidic acid (LPA), an important intermediary in lipid metabolism, depresses the main excitatory and inhibitory synaptic systems by different mechanisms. LPA depresses inhibitory synaptic transmission by reducing the number of postsynaptic receptors at inhibitory synapses; whereas it depresses excitatory synaptic transmission by decreasing the size of the ready-to-use synaptic vesicle pool at excitatory terminals. Finally, we demonstrate that LPA signaling contributes to the performance of motor output commands in adult animals. Our data documents that synaptic strength and neuronal activity are modulated by products of membrane phospholipid metabolism, which suggests that bioactive phospholipids are candidates in coupling brain function to the metabolic status of the organism.

## Introduction

Activity-dependent plasticity of neuronal networks refers to the adaptive changes in their properties in response to external and internal stimuli. In a prominent form of central nervous system (CNS) plasticity, synaptic strength results in an increase (potentiation) or decrease (depression) of transmission efficacy, depending on the neuron’s precedent activity (activity-dependent synaptic plasticity). Short-lived processes that modify synaptic strength occur in practically all types of synapses [1], and short-term synaptic plasticity is essential in regulating neuronal excitability and is central to information processing at both cellular and neuronal network levels [2]. Homeostatic adjustment of synaptic weights counteracts neuronal rate disturbances that affect self-tuning neuronal activity within a dynamic range via Ca^2+^-dependent sensors [3]. The number of receptors in the surface membrane and at synaptic sites, and the size of the readily releasable pool (RRP) of synaptic vesicles (SVs), are important determinants of synaptic strength, short-term plasticity, and intersynaptic crosstalk [4–8]. Unmasking the feedback mechanisms that are believed to sense neuron activity and adjust synaptic strength (i.e., activity-dependent, coupled messenger synthesis and/or release) would help to explain how circuits adapt during synaptic homeostasis, experience-dependent plasticity, and/or synaptic dysfunctions that underlie cognitive decline in many neurological diseases.

The ligand-gated ionotropic channels—A-type GABA_A_ receptors (GABA_A_Rs) and AMPA-type glutamate receptors (AMPARs)—mediate fast synaptic transmission at the vast majority of inhibitory and excitatory synapses, respectively, in the mammalian brain [4,5,9]. Cell surface stability of receptors is further regulated by post-translational phosphorylation, palmitoylation, and/or ubiquitination. In particular, AMPAR and GABA_A_R phosphorylation modulates the receptor’s biophysical properties and membrane trafficking. Hence, the coordinated activity of kinases and phosphatases plays a pivotal role in controlling synaptic strength and neuronal excitability. Key residues within the intracellular domains of diverse AMPAR and GABA_A_R subunits are targeted by a number of kinases, including protein kinases A and C, calcium/calmodulin-dependent kinase II, and tyrosine kinases of the Src family. Generally, phosphorylation stabilizes the receptor on the surface and, conversely, dephosphorylation appears to be important for receptor endocytosis [4,9].

Lysophosphatidic acid (LPA) is a strong candidate to function as a local messenger that rapidly affects synaptic strength. A membrane-derived bioactive phospholipid that affects all biological systems, LPA is an important intermediary in lipid metabolism and has a vital role in de novo biosynthesis of membrane phospholipids [10]. The nervous system is markedly modulated by LPA signaling. LPA, autotaxin (the main LPA-synthesizing enzyme), and many subtypes of LPA-specific G-protein-coupled receptors (LPA_1–6_) are enriched in the brain [10–12]. Downstream signaling cascades mediating LPA signaling include mitogen-activated protein kinase (MAPK) activation, adenylyl cyclase inhibition or activation, phospholipase C (PLC) activation/Ca^2+^ mobilization and/or protein kinase C (PKC) activation, arachidonic acid release, Akt/PKB activation, and the activation of small GTPase RhoA and subsequent Rho kinase (ROCK) stimulation [10]. Many subtypes of LPA receptors (LPARs) are expressed in the brain; in particular, LPA_1_ is highly expressed and is the most prevalent receptor subtype in both the embryonic and adult brains [13–15]. Accordingly, LPA targets all CNS cell types to modulate developmental processes including neurogenesis, migration, differentiation, and morphological and functional changes [10]. However, little is known about how LPA signaling influences neuron physiology and neuronal connectivity or integrates incoming synaptic drive. Presynaptic LPA_2_ at glutamatergic synapses mediates neuronal network hyperexcitability in an epileptic mouse model [16]. In addition, LPA_1_-deficient mice manifest alterations in managing diverse neurotransmitters [17–20]. Endogenous ROCK activity, an intracellular partner in LPA signaling, is necessary to maintain afferent AMPAergic and GABA_A_ergic synaptic strength in motoneurons [8]. As a conventional link in synaptic plasticity, activity-dependent LPA production occurs downstream of noxious activation of glutamate receptors in models of neuropathic pain [21]. However, whether LPA signaling is actually able to modulate synaptic strength and mediate activity-dependent synaptic plasticity remains unresolved.

The aim of this study was to investigate whether LPA regulates synaptic strength and plasticity of motoneuron excitatory and inhibitory synapses. Here, we show that LPA—mainly via LPA_1_—induced rapid and reversible depression in synaptic strength (short-term depression [STD]), and operated as an autocrine messenger mediating activity-dependent STD at inhibitory synapses. At glutamatergic synapses, presynaptic LPA signaling reduced the size of the RRP of SVs. At GABAergic synapses, postsynaptic LPA action mediated dephosphorylation and endocytosis-dependent internalization of the GABA_Aγ2_ subunit. Strikingly, LPA signaling regulated the performance of motor output commands in vivo. Therefore, LPA seems to have important implications for synaptic plasticity, pathology, and information processing in the brain.

## Results

The hypoglossal motor system was used as an experimental model to test the hypothesis that LPA regulates synaptic function. Hypoglossal motoneurons (HMNs) are arranged in the hypoglossal nucleus (HN) at the dorsal medulla, being easily accessible for functional studies in animal models. In vitro, AMPAR- and GABA_A_R-mediated neurotransmission incoming to HMNs can be feasibly isolated and are well characterized [8]. From an experimental point of view, a considerable advantage of this system is that the inspiratory-related afferent activity in HMNs, almost exclusively mediated by AMPAergic signaling, persists even in the in vivo decerebrated preparation [22,23]. Interestingly, HMNs underpin essential motor commands for normal suckling behavior in the neonate, a vital activity altered in LPA_1_-deficient mice [24]. In addition, *lpa*_*1–6*_ mRNAs are expressed by HMNs in the adult mouse (Allen Mouse Brain Atlas, http://mouse.brain-map.org/; [25]). Altogether, previous findings point to this motor system as a suitable model to investigate the role of LPA in the control of excitatory and inhibitory synaptic neurotransmission at the CNS.

### Anatomical Support for a Role of Lysophospholipids at Excitatory and Inhibitory Synapses

**Fig 1:**
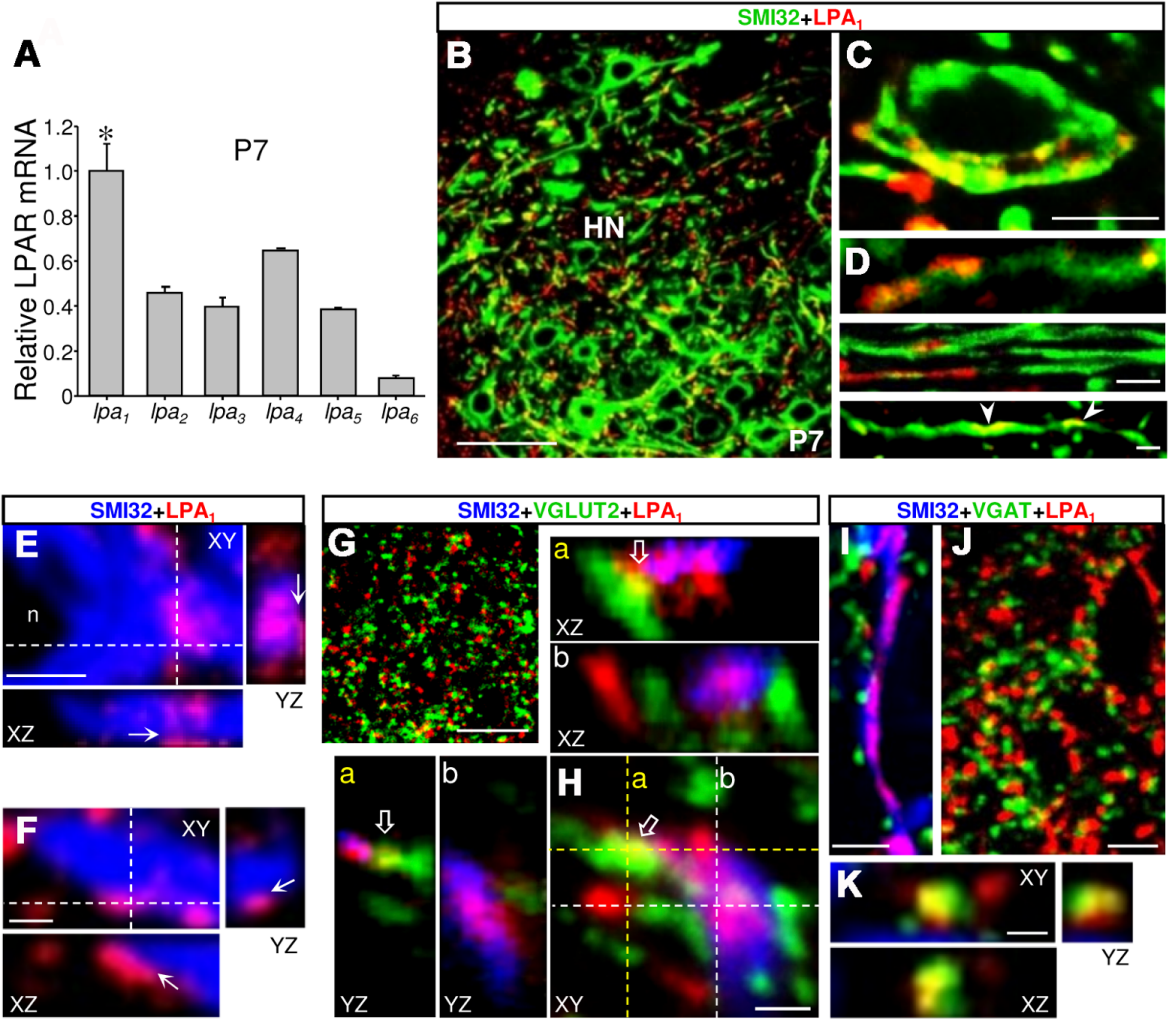
Expression pattern of LPA_1_ in the HN supports a role for lysophospholipids in the control of synaptic neurotransmission. (A) Expression levels of mRNA for indicated LPARs obtained by qRT-PCR of microdissected HNs from neonatal rats (P7) relative to the housekeeping GAPDH. Values were normalized taking mean value for *lpa*_***1***_ as 1. *p < 0.05, one-way ANOVA on Ranks relative to *lpa*_***2–6***_. Plot data can be found in S1 Data. (B–K) Multiple immunolabeling confocal images of the HN from P7 rats showing a close relationship between LPA_**1**_-ir patches and structures expressing the nonphosphorylated form of neurofilament H (SMI32), a motoneuron marker (B–F), VGLUT2- (G, H, open arrows), and/or VGAT-ir (I-K) inputs. The 18.8 ± 2.1% (*n* = 35 HMNs) of VGLUT2-ir and 11.9 ± 1.6% (*n* = 51 HMNs) of VGAT-ir inputs apposed to HMN somata colocalized with LPA_**1**_-ir patches. Note that in 3-D reconstructions (E, F, and Hb), LPA_**1**_ staining colocalizes and borders SMI32-ir somata and dendrites (E, F, arrows). The *yz*- and *xz*-planes also confirm colocalization of LPA_**1**_-ir with excitatory (Ha, open arrows) and inhibitory (K) inputs. Finally, VGLUT2- (Hb) and VGAT-ir (I) inputs appeared apposed on LPA_**1**_-containing SMI32-ir dendrites. The *xz*- and *yz*-planes are located as indicated by the white dashed lines. Scale bars: B, 50 μm; G, 25 μm; C, D, and J, 10 μm; E and I, 5 μm; and F, H, and K, 2 μm.

To explore a possible role of LPA in shaping the normal motor output of the HN, it was necessary to determine the predominant isotype of its main target receptors expressed in this motor nucleus. Assessment of the expression levels of mRNAs for LPA_1–6_ receptors in microdissected HN from neonatal (P7) rats revealed that *lpa*_*1*_ mRNA was 1.5 to 12.5 times more abundant than *lpa*_*2–6*_ transcripts (Fig 1A). Subsequently, confocal analysis of double immunolabeled HN from P7 pups showed LPA_1_-immunoreactive (ir) puncta, patches, and fiber-like structures colocalizing with SMI32-positive HMN perikarya and dendrite-like structures (Fig 1B–1D). Three-dimensional reconstructions agreed with a cytoplasmic and membrane localization of LPA_1_ in perikarya and main dendritic branches of HMNs (Fig 1E and 1F). Triple immunofluorescence for LPA_1_, SMI32, and the vesicular glutamate (VGLUT2) or GABA (VGAT) transporters as synaptic markers confirmed that LPA_1_-ir puncta were colocalizing with excitatory (VGLUT2-ir) or inhibitory (VGAT-ir) presynaptic structures (Fig 1G, 1H, 1J, and 1K). Both excitatory and inhibitory inputs were also found apposed to SMI32-ir neuropil or somata coexpressing LPA_1_ (Fig 1H and 1I). Although LPA_1_ expression in other neural cell types is not excluded, this expression pattern supports pre and/or postsynaptic roles of LPA_1_ at the main excitatory and inhibitory inputs on HMNs, suggesting a potential contribution of LPA to motoneuron physiology.

### LPA Induces STD of Excitatory and Inhibitory Inputs in a Dose-Dependent Manner

**Fig 2:**
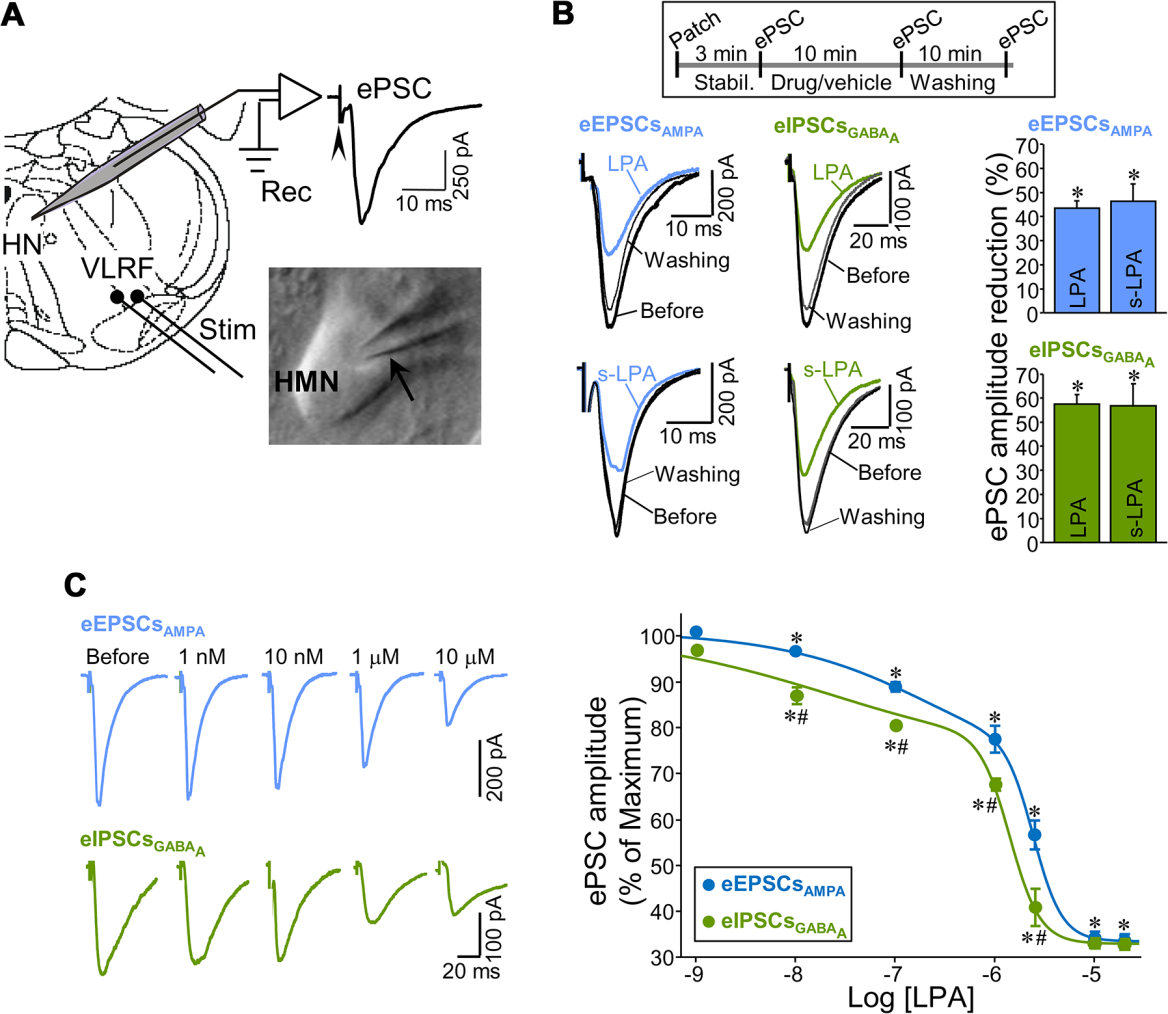
LPA induces STD at excitatory and inhibitory synapses in a dose-dependent manner. (A) Schematic diagram of the in vitro experimental model used to analyze the effects of LPA on synaptic transmission incoming to HMNs. Whole-cell patch-clamp recordings (Rec) were obtained from somata of HMNs in neonatal brain stem slices. Experiments were performed as in our previous published study [8]. ePSCs were evoked by electrical stimulation (Stim) of the VLRF. A micropipette (arrow) near to a HMN before patch performance is illustrated in the inset. (B) Top, schematic representation of the experimental protocol carried out to study drug effects on ePSCs. Motoneurons were initially allowed to stabilize (Stabil.) with normal artificial cerebrospinal fluid (aCSF) to obtain baseline control recordings. Slices were then superfused for 10 min with aCSF alone (vehicle) or with LPA or s-LPA (drug) before current responses were acquired again. Finally, a last round of acquisition was taken after a 10 min washout with drug-free aCSF. Bottom, examples of eEPSCs_**AMPA**_ (left panels) and eIPSCs_**GABAA**_ (middle panels) recorded from HMNs before and following exposure to LPA (2.5 μM) or s-LPA (40 μM) for 10 min and after washing. Right panels, reduction in the eEPSCs_**AMPA**_ (blue; *n* > 10 HMNs per drug) or eIPSCs_**GABAA**_ (green; *n* = 4 HMNs per drug) amplitude for LPA- or s-LPA-treated groups compared to their respective pretreatment (before) periods. eEPSCs_**AMPA**_ or eIPSCs_**GABAA**_ were pharmacologically isolated in the presence of 1 μM strychnine hydrochloride, 30 μM d-tubocurarine, 50 μM (DL)-APV, and 10 μM bicuculline methochloride or NBQX (20 μM), respectively, continuously applied to the bath perfusion. (C) Left panel, examples of eEPSCs_**AMPA**_ (top) and eIPSCs_**GABAA**_ (bottom) recorded from two HMNs before and during exposure to the indicated LPA concentrations. This experimental design was carried out in three HMNs per each synaptic category resulting in dose-dependent attenuation of the ePSCs similar to those presented in the plot. Right panel, reduction in eEPSCs_**AMPA**_ and eIPSCs_**GABAA**_ amplitude induced by LPA at the indicated concentrations relative to control (before) condition. Each HMN was exposed to a single dose of LPA. Data for each drug concentration were averaged from at least three independent experiments. Mean changes in amplitude obtained after 10 min incubation with vehicle (aCSF, S1 Text) were subtracted from alterations induced by each tested LPA concentration. The study included only those motoneurons that recovered after washing to at least the percentage of change obtained by vehicle perfusion. *n* ≥ 4 HMNs per each concentration and synaptic signaling system. *p < 0.05, one-way RM-ANOVA relative to ePSCs recorded before lysophospholipid incubation. #*p* < 0.05, one-way ANOVA on Ranks relative to reduction in amplitude of eEPSCs_**AMPA**_ measured after the same LPA concentration. Plots data can be found in S1 Data.

Next, we investigated the functional effects of LPA on glutamatergic and GABAergic synaptic currents by whole-cell patch-clamp recordings of HMNs (slices from P6–P9 rats). Electrical stimulation of the ventrolateral reticular formation (VLRF) evoked postsynaptic currents (ePSCs) in HMNs (Fig 2A). The AMPAR- or GABA_A_R-mediated components of ePSCs (excitatory [eEPSCs_AMPA_] or inhibitory postsynaptic currents [eIPSCs_GABAA_], respectively) were isolated and recorded as described in S1 Text.

The two major species of LPA (approximately 70%) found in the brain [26], monounsaturated (18:1, or LPA) and saturated (18:0, or s-LPA), were used in this study. While LPA activates LPA_1–3_, s-LPA has high affinity for LPA_1/2_, but is a comparatively poor agonist against LPA_3_ [27]. Unless stated otherwise, LPA was used at a similar concentration (2.5 μM) to that found in serum (1–5 μM) [28]. In general, unsaturated LPAs are more potent than s-LPA in activating LPARs and inducing biological activities [29]. Accordingly, a higher concentration was used for s-LPA (40 μM) than for LPA (2.5 μM) to achieve a similar effect on neurotransmission. Both phospholipids, added for 10 min to the bath solution, strongly attenuated the amplitude of eEPSCs_AMPA_ and eIPSCs_GABAA_ (Fig 2B). The effects were reversed after 10 min of washing. Thus, LPA modulated rapidly and reversibly the strength of AMPAR- and GABA_A_R-mediated synaptic transmission in motoneurons.

The tested dose (2.5 μM) of LPA had a proportionately higher effect on inhibitory than on excitatory inputs (Fig 2B and 2C). Further, differential sensitivity to LPA was studied by applying various concentrations, ranging from 1 nM to 20 μM. After subtracting vehicle-induced changes (S1 Text), an effect on both currents was detectable at concentrations as low as 10 nM and increased with LPA concentration to a similar maximum reduction in both currents (approximately 70%) at 10–20 μM (Fig 2C). Dose-response relationships were well fitted (*p* < 0.001; r^2^ > 0.99) by biphasic (two slopes) five-parameter logistic equations, suggesting that LPA affects synaptic neurotransmission by multiple mechanisms. It remains to be determined whether this is the consequence of the recruitment of diverse isoreceptors and/or downstream signaling pathways. In any case, from the nanomolar to first-order micromolar range, LPA diminished inhibitory inputs (IC_50_ = 1.0 ± 0.17 μM) in greater proportions (*p* < 0.001, Kolmogorov-Smirnov test) than excitatory ones (IC_50_ = 1.8 ± 0.08 μM), but at higher concentrations, LPA affected both synaptic systems similarly (Fig 2C).

### LPA Operates Presynaptically at Excitatory Inputs

**Fig 3:**
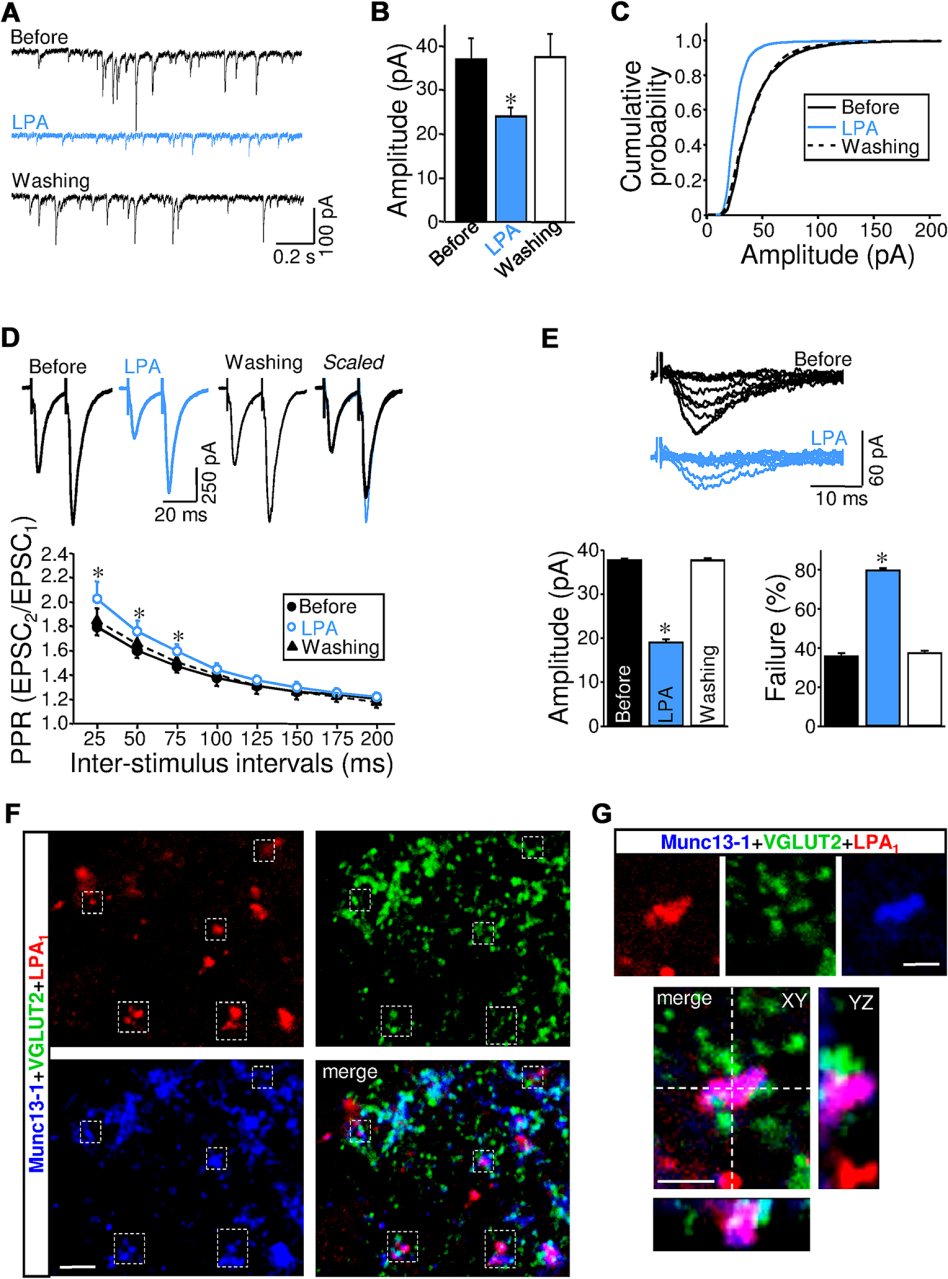
Presynaptic LPA signaling induces excitatory STD. (A) Current traces of sEPSCs_**AMPA**_ recorded from a HMN at the indicated conditions. The recording of sEPSCs_**AMPA**_ was performed under conditions of facilitated synaptic release without TTX in a modified extracellular solution containing high-Ca^2+^ (4 mM), high-K^+^ (9 mM), and the receptor antagonists indicated in Fig 2B. (B) Mean sEPSCs_**AMPA**_ amplitude for LPA-treated group (2.5 μM) compared to their respective pretreatment (before) and washout periods (*n* = 4 HMNs). (C) Normalized cumulative probability distributions of sEPSCs_**AMPA**_ amplitude for each condition. Bin width: 2 pA. Note that the cumulative distribution of sEPSCs_**AMPA**_ amplitude shifted to the left (*p* < 0.001; Kolmogorov-Smirnov test). (D) Top, eEPSCs_**AMPA**_ recorded in a HMN at the indicated conditions in response to paired-pulse stimulation of VLRF. The rightmost trace shows the superimposition of the responses scaled to the peak of the first eEPSCs_**AMPA**_. Bottom, comparison of PPR measured at specified interpulse intervals for HMNs recorded at the indicated conditions (*n* = 4 HMNs). Paired-pulse ratio (PPR) was obtained from the amplitude of the first and second eEPSCs_**AMPA**_ by the formula eEPSCs_**AMPA**_2/eEPSCs_**AMPA**_1. The stimulus intensity was adjusted so that the eEPSC_**AMPA**_1 was approximately 50% of maximal amplitude, then maintained constant throughout the recording period. (E) Top, superimposition of 10 successive eEPSCs_**AMPA**_ evoked at 0.2 Hz by minimal stimulation of VLRF in HMN before and after treatment with LPA. Characteristically, the intensity of the stimulation was set to elicit eEPSCs_**AMPA**_ with 30% to 40% failure at the control (before) condition. Bottom, mean eEPSCs_**AMPA**_ amplitude (left) and failure rate (right) at indicated conditions (*n* = 4 HMNs). Experiments and analysis described in A–E have been performed as in our previously published study [8]. *p < 0.05, one-way (B, E) or two-way (D) RM-ANOVA relative to control (before) condition. (F) Confocal images of the HN obtained from P7 rats processed by triple immunolabeling for LPA_**1**_, VGLUT2 and the presynaptic active zone (a.z.) marker Munc13-1. Note triple colocalizations within the boxed areas. (G) 3-D reconstruction showing LPA_**1**_ expression in the presynaptic a.z. of a glutamatergic bouton. Note that LPA-ir colocalizes with Munc13-1 and with a VGLUT2-ir SVs pool in the three planes. The *xz*- and *yz*-planes are located as indicated by the white dashed lines. Scale bars: F, 5 μm; G, 2 μm. Plots data can be found in S1 Data.

As in our previously published study [8], a combined electrophysiological analysis was performed to identify the LPA synaptic site of action. LPA signaling on AMPAR-mediated transmission is likely not attributable to changes in postsynaptic sensitivity to glutamate. LPA did not alter the amplitude in both the miniature quantal EPSCs_AMPA_ (mEPSCs_AMPA_) and postsynaptic currents evoked by exogenous glutamate pulses (S1 Text; S1 Fig). For that reason, we sought evidence for a presynaptic mechanism by recording spontaneous AMPAergic synaptic currents under facilitated spontaneous glutamate release (sEPSCs_AMPA_). In this condition, synaptic activity was a mixture of action potential-dependent and -independent events. After LPA treatment, the sEPSCs_AMPA_ amplitude, but not frequency (10.8 ± 1.0 Hz, *p* = 0.761), reversibly decreased to a value similar to that recorded for mEPSCs_AMPA_ in control condition (before: 36.0 ± 3.8 pA; LPA: 24.0 ± 2.0 pA; Fig 3A–3C). This agrees with a LPA-induced full inhibition of action potential-dependent events.

In addition, we evaluated eEPSCs_AMPA_ facilitation using paired-pulse and repetitive afferent stimulation protocols as in our previously published study [8]. Under repetitive stimulation, a change in the amount of facilitation is considered to be attributable to a presynaptic change in the release probability of neurotransmitter quanta [1]. In the control condition, paired-pulse stimulation displayed a strong facilitation of eEPSCs_AMPA_ over the entire range of interstimulus intervals tested, but this was more pronounced at shorter interstimulus intervals (Fig 3D; S2 Fig). Facilitated PPR (paired-pulse ratio) showed a marked and reversible increase at 25 ms and 50 ms intervals after application of either s- or LPA (abbreviated as s-/LPA; Fig 3D; S2 Fig). On average, LPA and s-LPA increased the magnitude of PPR by 12.8% and 29.3% at 25 ms, respectively. The finding that LPA also reversibly potentiated the facilitation index of eEPSCs_AMPA_ under repeated VLRF stimulation provided additional evidence in support of these outcomes (S1 Text; S3 Fig).

At this point in our study, the attenuation of eEPSCs_AMPA_ induced by LPA was related to a reduction in the glutamate release probability, which is believed to be determined by the number of fusion-competent SVs or the size of the RRP of SVs [6,7]. This idea was further strengthened by a subsequent analysis of eEPSCs_AMPA_ amplitude using the minimal stimulation paradigm, designed to stimulate only one fiber and a single or small number of release sites. As in our previous study [8], the intensity of the stimulation was set to elicit eEPSCs_AMPA_ with 30% to 40% failure (Fig 3E). In this context, LPA treatment evoked a significant reduction of the mean amplitude of eEPSCs_AMPA_ elicited by minimal stimulation and an enhancement of the eEPSCs_AMPA_ failure rate (Fig 3E; S4 Fig). The presynaptic action of LPA on glutamatergic inputs is further supported because LPA_1_-ir puncta colocalize with Munc13-1, a presynaptic active zone (a.z.) marker [30], in VGLUT2-containing boutons (Fig 3F and 3G). The LPA_1_ association with a region of the presynaptic membrane compromised in the fusion of SVs supports that LPA signaling has a direct relationship with the machinery involved in the regulation of neurotransmitter release.

### LPA Modulates Excitatory Inputs via LPA_1_/G_α i/o_-PLC

**Fig 4:**
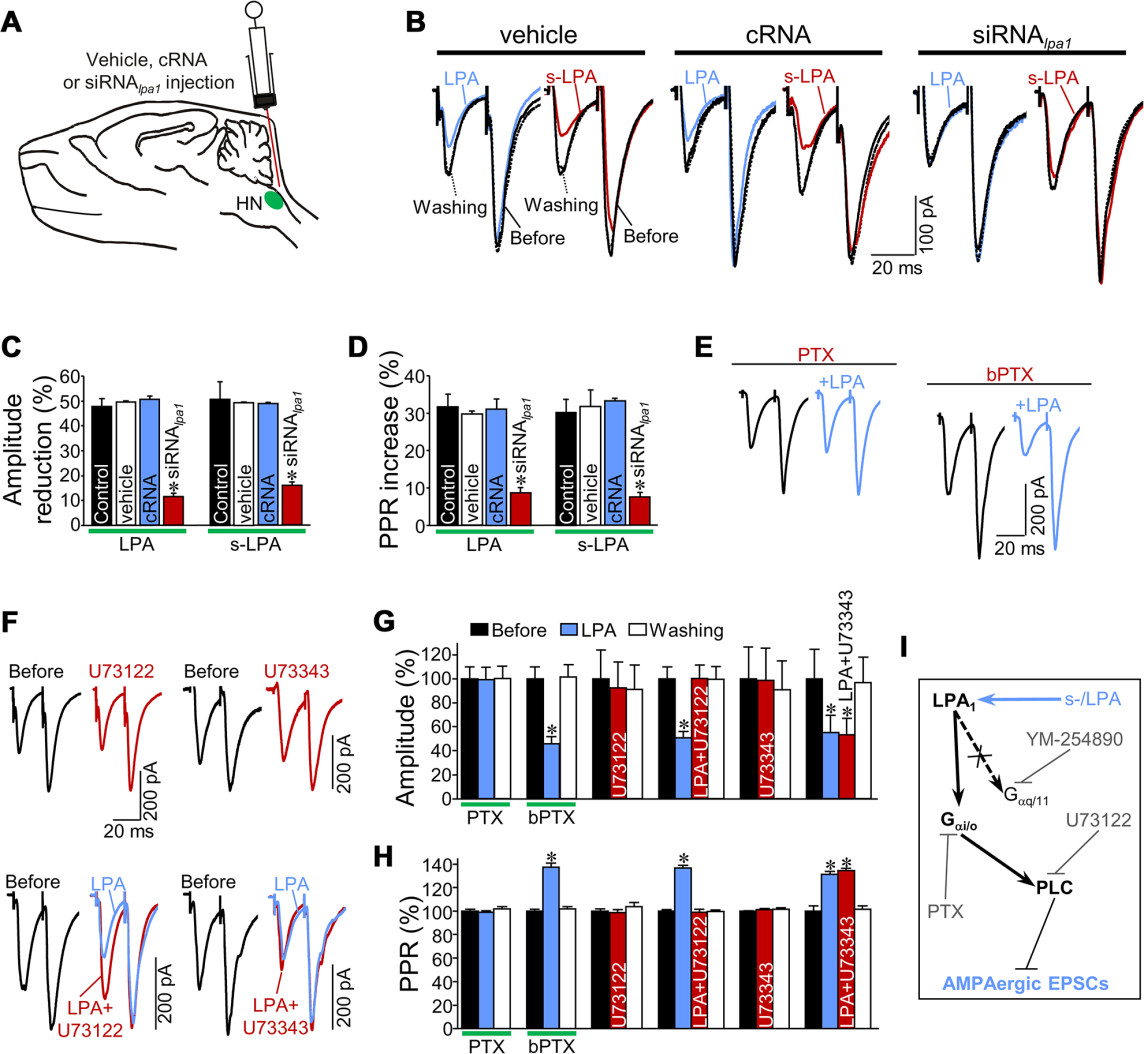
LPA modulates AMPAR-mediated neurotransmission via LPA_1_/G_αi/o_-PLC. (A) Schematic diagram of microinjections performed in the fourth ventricle of neonatal rats at P4. A solution (2 μl) containing the vehicle (RNase-free phosphate-buffered saline [PBS]), control noninterfering RNA (cRNA, 2 μg) or a small interfering RNA directed against *lpa*_***1***_ (siRNA_***lpa1***_, 2 μg) was administered by means of a Hamilton syringe. (B) Representative eEPSCs_**AMPA**_ recorded in HMNs obtained from animals receiving the specified treatments recorded at the indicated conditions. (C, D) Mean eEPSCs_**AMPA**_ amplitude reduction (C, in percent) and PPR ratio increase (D, in percent) in response to addition to the bath of LPA (2.5 μM) or s-LPA (40 μM) measured at 25 ms interpulse intervals for HMNs recorded under the indicated treatments (control, LPA: *n* = 13 HMNs, s-LPA: *n* = 6 HMNs; vehicle, LPA: *n* = 6 HMNs, s-LPA: *n* = 4 HMNs; cRNA, LPA: *n* = 6 HMNs, s-LPA: *n* = 4 HMNs; siRNA, LPA: *n* = 9 HMNs, s-LPA: *n* = 5 HMNs). *p < 0.05, one-way ANOVA relative to control, vehicle and cRNA conditions. (E) Effect of LPA on eEPSCs_**AMPA**_ from two HMNs in response to paired-pulse stimulation under the presence of the G_**αi/o**_ inhibitor pertussis toxin (PTX) (100 ng/ml; left) or the noncatalytic B oligomer of PTX (bPTX) (100 ng/ml; right). Slices were preincubated for 2 h with PTX or bPTX before recordings began and were maintained throughout the experimental procedure. (F) Representative eEPSCs_**AMPA**_ from 4 HMNs in response to paired-pulse stimulation of VLRF showing the effects of the PLC inhibitor U73122 (1 μM) or its inactive analog U73343 (5 μM) per se (top) or coadded after previous incubation for 10 min with LPA (bottom). (G, H) Mean eEPSCs_**AMPA**_ amplitude and PPR ratio (25 ms interpulse intervals) under the indicated treatments (PTX and bPTX, *n* = 6 HMNs; U73122, *n* = 5 HMNs; LPA+U73122, *n* = 5 HMNs; U73343, *n* = 4 HMNs; LPA+U73343, *n* = 5 HMNs). *p < 0.05, one-way RM-ANOVA relative to control (before) condition. (I) Diagram of the proposed pathway mediating LPA-induced STD at AMPAergic signaling, indicating drug targets. Plots data can be found in S1 Data.

The qRT-PCR and immunohistochemical studies, together with additional pharmacological tests (S1 Text: S5 Fig; S6 Fig), robustly point to LPA_1_ as a pivotal LPAR affecting glutamatergic synapses. In this context, injection of a small interfering RNA (siRNA) against *lpa*_*1*_ (siRNA_*lpa1*_; 2 μg/2 μl) into the fourth ventricle efficiently reduced LPA_1_ expression in the brain stem (Fig 4A; S1 Text; S7 Fig). siRNA_*lpa1*_ robustly diminished, but did not fully avoid, (s-)LPA-induced alterations on eEPSCs_AMPA_ amplitude and PPR relative to the administration of control noninterfering siRNA (cRNA; 2 μg/2 μl) or vehicle (RNase-free phosphate buffered saline; 2 μl) (Fig 4A–4D). Whether the remaining response of eEPSCs_AMPA_ to (s-)LPA could be due to residual LPA_1_ expression or to recruitment of compensatory mechanisms—e.g., via up-regulated LPA_3_ in response to LPA_1_ knockdown—remains to be elucidated.

LPA_1_ couples with and activates three G proteins: G_α12/13_, G_αi/o_, and G_αq/11_ [10]. Previous findings [8] and pharmacological data (S1 Text) did not support G_α12/13_ involvement. Alternatively, preincubation for 2 h with the G_αi/o_ specific inhibitor pertussis toxin (PTX), but not with the noncatalytic B oligomer of PTX (bPTX), prevented (s-)LPA-induced STD and PPR increase (Fig 4E, 4G, and 4H; S8A, S8D, and S8E Fig). Cascade downstream of lysophospholipids included PLC activation; the PLC inhibitor U73122, but not its inactive analog U73343, reversed—to a control-like state—the changes in amplitude and PPR provoked by (s-)LPA (Fig 4F–4H; S8B, S8D, and S8E Fig). Finally, the G_αq/11_ inhibitor YM-254890 did not interfere with s-LPA effects on eEPSCs_AMPA_ (S8C–S8E Fig). Altogether, these findings indicate that LPA signaling controls excitatory inputs via presynaptic G_αi/o_-protein-coupled LPA_1_ and PLC (Fig 4I).

### LPA Signaling Reduces the Size of RRP of SVs via MLCK in Excitatory Boutons

**Fig 5:**
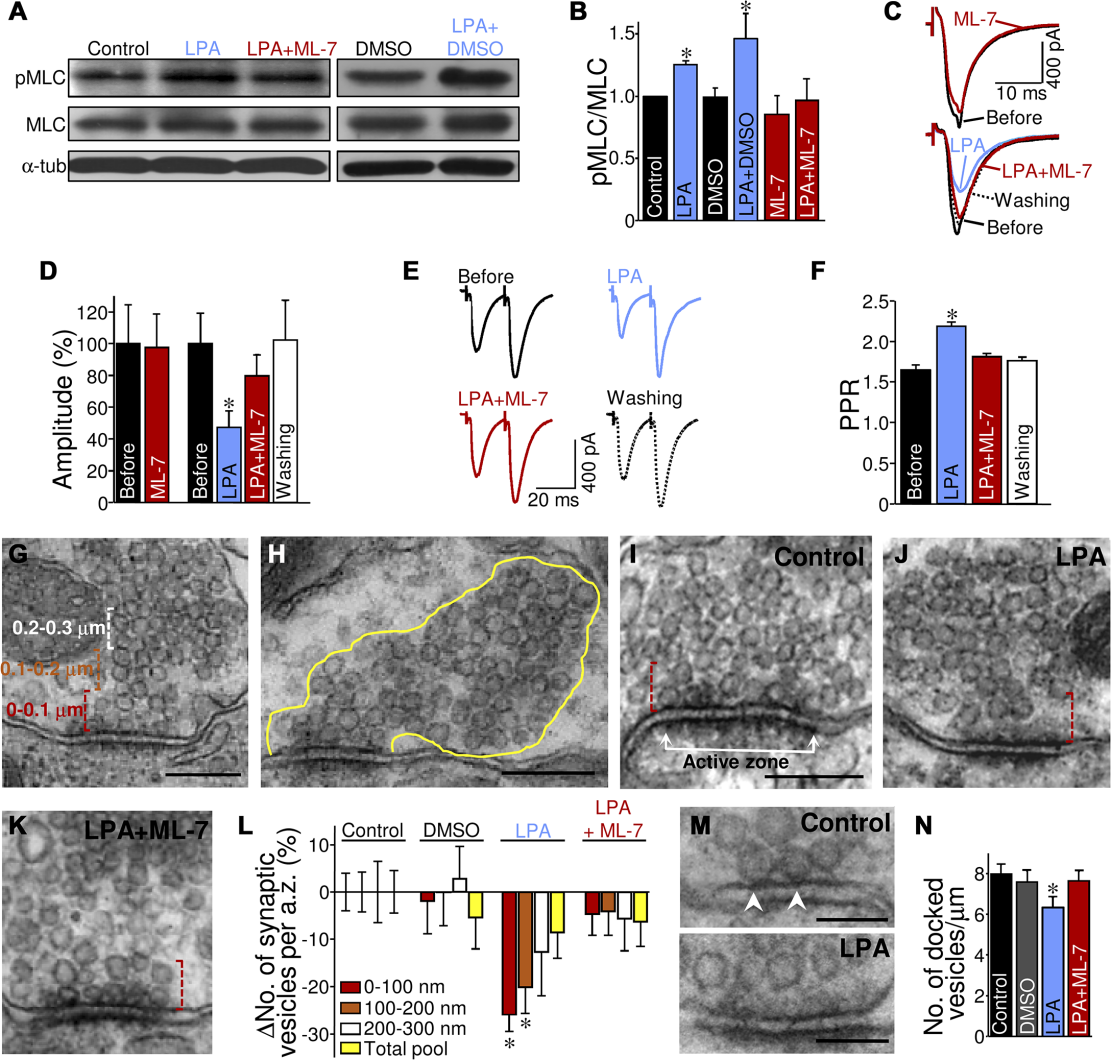
LPA rearranges SVs at excitatory boutons in a MLCK-dependent manner. (A) Western blot of phosphorylated and total MLC protein levels (denoted as pMLC and MLC, respectively) in the HN of neonatal brain stem slices incubated for 10 min in aCSF alone (control) or supplemented with either LPA (2.5 μM), vehicle (0.2% DMSO), LPA + vehicle, or LPA + ML-7 (10 μM). α-tubulin (α-tub) expression was the internal loading reference. (B) Histogram showing the average ratio of pMLC to total MLC densitometric intensity for the control and treated slices. Ratio values were normalized relative to the control group. Columns represent the average of at least three independent experiments. *p < 0.05, one-way ANOVA on Ranks relative to control condition. (C) eEPSCs_**AMPA**_ recorded from two HMNs in normal aCSF and after 10 min bath perfusion with the indicated combination of drugs. (D) Average eEPSC_**AMPA**_ amplitude for the ML-7 (*n* = 5 HMNs) and LPA+ML-7 (*n* = 7 HMNs) treated groups of HMNs compared with their respective pretreatment controls (before). (E) eEPSCs_**AMPA**_ evoked in HMNs by paired-pulse stimulation of VLRF before and following treatment with LPA and finally after coaddition of ML-7. (F) Changes in PPR of eEPSCs_**AMPA**_ measured in HMNs exposed sequentially to LPA and LPA+ML-7. *p < 0.05, one-way RM-ANOVA relative to the control condition in D and F. (G, H) Electron micrographs of two S-type boutons (containing spherical vesicles) with asymmetric synaptic contacts on the somatic membrane of a HMN depicting details of the procedure used to examine topographically the numerical changes in SVs. The number of SVs was counted in three zones, each 0.1 μm wide, parallel to the membrane of the synaptic cleft and at successively greater distances from the a.z. (G). The first region (red dashed line) encloses an area directly adjacent to the a.z. membrane. The intermediate region (orange dashed line) was located in the interval from 0.1 μm to 0.2 μm away from the a.z. Finally, the more distant region (white dashed line) occupied an area corresponding to the distance interval from 0.2 μm to 0.3 μm. The total number of SVs contained in each bouton section was also quantified (H). (I–K) Electron micrographs of S-type boutons in contact with the somatic membrane of HMNs from neonatal rats following incubation (10 min) of brain stem slices in aCSF alone (control) or supplemented with LPA or LPA+ML-7 at concentrations indicated in A. The boxed region (red dashed line) encloses the area directly adjacent to the a.z. membrane. (L) Quantitative changes in the number of SVs (expressed as percentage change from control) are shown in each spatial compartment. Histogram bins indicate distances from the a.z. as indicated in the legend. Increment in the number of the total pool of SVs per bouton section is also illustrated (yellow bars). (M) High-magnification electron microscopy images showing in detail the SVs (membranes in contact with the presynaptic density) docked to the a.z. (arrowheads). Scale bars: G–K, 200 nm; M, 100 nm. (N) Histogram showing the linear density of docked SVs per μm of a.z. under the indicated conditions. Control, *n* = 133 boutons/a.z.; vehicle, *n* = 54/104 boutons/a.z.; LPA, *n* = 102 boutons/a.z.; LPA plus ML-7, *n* = 102 boutons/a.z. *p < 0.05, one-way ANOVA relative to the control condition. Experiments and analysis were performed as in our previous published study [8]. Plots data can be found in S1 Data.

LPA induces smooth muscle contraction in a PLC-dependent, ROCK-independent manner that involves myosin light chain (MLC) phosphorylation by MLC kinase (MLCK) [31]. These findings point to MLCK as a potential kinase mediating the presynaptic action of LPA on excitatory neurotransmission. Accordingly, LPA increased the p-MLC:MLC ratio in the HN relative to aCSF-incubated brain stem slices, which was fully prevented by coincubation with the specific MLCK inhibitor ML-7 (Fig 5A and 5B). In concordance, though ML-7 per se did not alter the amplitude of eEPSCs_AMPA_, as we also recently reported [8], it fully suppressed LPA-induced alterations on eEPSCs_AMPA_ amplitude and PPR (Fig 5C–5F). This further supports MLCK as a main molecular substrate activated by LPA signaling within excitatory presynaptic terminals.

MLC phosphorylation stimulates actomyosin interactions [32], and presynaptic Ca^2+^ concentration regulates MLCK activity and modulates the RRP size in the calyx of the Held synapse [33]. Therefore, LPA signaling, through its modulatory control on MLCK and the actomyosin cytoskeleton, might regulate clustering and spatial distribution of SVs within excitatory (S-type, spherical SVs-containing) boutons (S1 Text). Electron microscopy analysis, performed as in our previous study [8], showed that, in a MLCK-dependent way, LPA noticeably reduced the number of SVs near the a.z. in S-type boutons attached to HMNs, compared to control conditions (Fig 5G–5L; S1 Text). In addition, LPA induced a drop (−20.2 ± 6.3%) in the SV population morphologically docked to (i.e., in contact with) the a.z., which corresponds to the release-ready neurotransmitter quanta [34] that was prevented by coaddition of ML-7 (Fig 5M and 5N). These outcomes robustly support that LPA signaling regulates the size of the RRP of SVs in S-type boutons by a MLCK-dependent mechanism.

Together, these data strongly suggest that the depression of synaptic strength induced by LPA treatment is dependent on a reduction in the probability of release from excitatory glutamatergic terminals. This effect is attributable, at least in part, to a reduction in the size of the RRP of SVs. Our results reaffirm that LPA signaling modulates excitatory synaptic transmission through mechanisms modulating the presynaptic component of the synapse.

### LPA-Induced Inhibitory STD Comprises Postsynaptic LPA_1_-RhoA/ROCK-CaN Signaling and GABA_Aγ2_ Dephosphorylation

**Fig 6:**
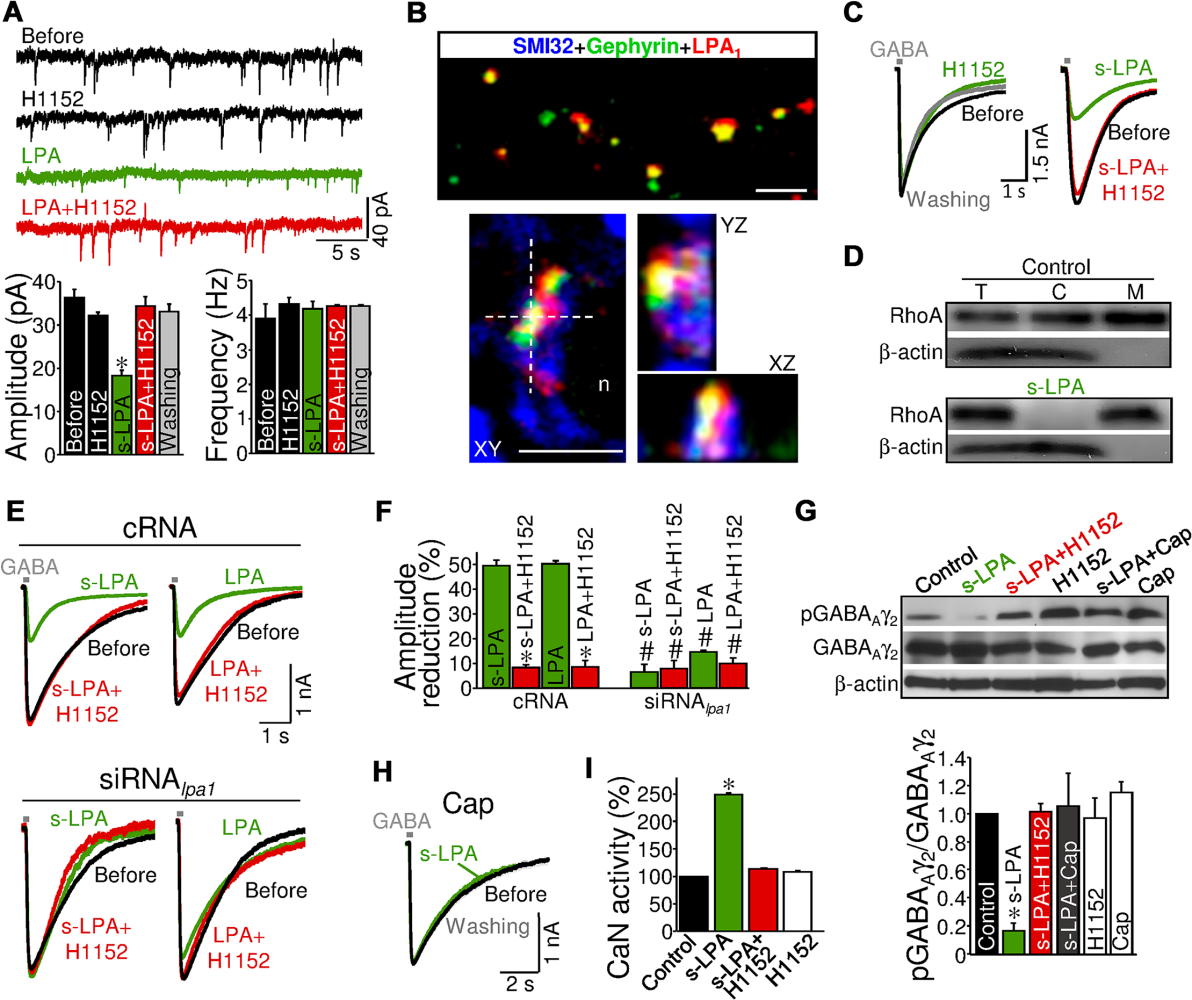
LPA induces GABA_A_ergic STD and GABA_Aγ2_ dephosphorylation via postsynaptic LPA_1_-RhoA/ROCK-CaN signaling. (A) Spontaneously occurring mIPSCs_**GABAA**_ recorded from a representative HMN before and after perfusion with the indicated combination of drugs. Bottom, mean mIPSCs_**GABAA**_ amplitude (left) and frequency (right) at indicated conditions (*n* = 5 HMNs). mIPSCs_**GABAA**_ were pharmacologically isolated in the presence of 1 μM tetrodotoxin (TTX), 1 μM strychnine hydrochloride, 30 μM d-tubocurarine, 50 μM (DL)-APV, and NBQX (20 μM) continuously applied to the bath perfusion. *p < 0.05, one-way RM-ANOVA relative to the control (before) condition. (B) Multiple immunolabeling confocal images of the HN from P7 rats showing colocalization between LPA_**1**_-ir and gephyrin-ir (top). 3-D reconstruction (bottom) showing that LPA_**1**_-ir colocalizes with gephyrin in a SMI32-ir HMN. n, HMN nucleus. The *xz*- and *yz*-planes are located as indicated by the white dashed lines. Scale bars: 5 μm. (C) Whole-cell GABA_**A**_ergic currents evoked by 100 ms pressure pulses of GABA (applied at saturating concentration; 1 mM) in two spinal motoneurons (SMNs) under indicated treatments. Recordings were performed in the presence of TTX in nominally Ca^2+^-free solution. (D) Western blots of total (T), cytosolic (C), and membrane-associated (M) RhoA in SMNs in untreated and s-LPA incubated (for 10 min) cultures. (E) Whole-cell GABA_**A**_ergic currents evoked by pulses of GABA in SMNs preincubated with cRNA (top) or siRNA_***lpa1***_ (bottom) before and after superfusion with the indicated drugs. (F) Summary data showing the changes in GABA_**A**_ergic currents measured in SMNs exposed at different treatments (*n* ≥ 5 SMNs per group). #, *p < 0.05, one-way ANOVA or RM-ANOVA, respectively, relative to s-LPA or LPA treatments of cRNA preincubated SMNs. (G) Western blot (top) and averaged ratio (bottom) of phosphorylated and total GABA_**Aγ2**_ subunit protein levels (denoted as pGABA_**Aγ2**_ and GABA_**Aγ2**_, respectively) in SMNs incubated (10 min) with aCSF alone (control) or supplemented with indicated drugs. β-actin was an internal loading reference. (H) Same as in C under indicated treatments. SMNs were preincubated for 30 min with the calcineurin (CaN) autoinhibitory peptide (Cap; 50 μM). (I) Changes of CaN activity in lysates from cultured SMNs untreated (control) or treated for 10 min with the indicated drugs. *p < 0.05, one-way ANOVA on Ranks relative to control condition. Plots data can be found in S1 Data.

Next, we explored whether LPA modulates GABAergic and glutamatergic synapses by similar mechanisms of action. Amplitude, but not frequency, of miniature quantal IPSCs_GABAA_ (mIPSCs_GABAA_) recorded in HMNs was reduced by LPA, in agreement with a postsynaptic site of action (Fig 6A; S9 Fig). The molecular cascade downstream of LPA is also distinct, since LPA-induced alterations on mIPSCs_GABAA_ were reversed by the ROCK inhibitor H1152 (Fig 6A; S9 Fig). H1152 also returned (s-)LPA-induced changes in eIPSC_GABAA_ amplitude to a control-like state (S10A and S10B Fig). In support of a non-presynaptic action of s-LPA on eIPSCs_GABAA_, the mean PPR remained similar to the control condition in the presence of s-LPA or s-LPA plus H1152 (S10C and S10D Fig). Colocalization in HMNs of LPA_1_-ir with the postsynaptic marker gephyrin, a clustering protein for GABA_A_Rs [35], strengthened the evidence of a postsynaptic site of action for LPA (Fig 6B).

Postsynaptic action and the molecular signaling underlying LPA-induced modulation of GABA_A_ergic system were assessed in primary cultures of spinal motoneurons (SMNs) (S1 Text; S11 Fig). The mean amplitude of inward GABA_A_R-mediated current evoked by exogenous GABA pulses (−4.13 ± 0.98 nA; *n* = 8 SMNs) was robustly reduced by s-LPA (−62.5 ± 10.1%, *p* < 0.001, one-way ANOVA for repeated measures (RM-ANOVA)), in a ROCK-dependent way (s-LPA+H1152: −3.23 ± 0.49 nA, *p* = 0.345) (Fig 6C). In addition, we observed that s-LPA activated the small GTP-binding protein RhoA, the major ROCK activator, in SMNs. This was evidenced by an s-LPA-induced increase (+78.3 ± 25.7%; *p* < 0.05, one-way ANOVA on Ranks) in the membrane (M):cytosolic (C) ratio of RhoA expression relative to the control condition (Fig 6D). Supplementary data support LPA signaling as the activator for the RhoA/ROCK pathway in motoneurons (S1 Text; S12 Fig). Furthermore, pretreatment with siRNA_*lpa1*_ prevented the effects of (s-)LPA on GABA_A_R-mediated currents compared to cRNA-treated SMNs, providing conclusive evidence of postsynaptic LPA_1_ involvement (Fig 6E and 6F; S1 Text; S13 Fig).

Phosphorylation of serine 327 on the GABA_Aγ2_ subunit (pGABA_Aγ2_) regulates GABA_A_R clustering and synaptic strength at inhibitory synapses [36,37]. Therefore, we investigated whether LPA_1_-ROCK signaling regulates phosphorylation of GABA_A_γ_2_. Contrary to expectations of a direct interaction between ROCK and GABA_Aγ2_, s-LPA induced a robust reduction (−83.3 ± 5.2%) of the pGABA_Aγ2_:GABA_Aγ2_ ratio in SMNs that was prevented by coaddition of H1152 (+1.6 ± 6.0%) (Fig 6G). This was also observed in the HN (S1 Text; S14 Fig). Strikingly, direct binding of the phosphatase calcineurin (CaN) to GABA_Aγ2_ subunits dephosphorylates Ser^327^ [37,38], which leads to a reduction in inhibitory postsynaptic current amplitude [37]. Therefore, recruitment of CaN (also named Ca^2+^/calmodulin-dependent phosphatase 2B), was proposed as a potential link between LPA_1_-ROCK signaling and GABA_Aγ2_ dephosphorylation.

Preincubation of SMNs with CaN autoinhibitory peptide (Cap; 50 μM) also prevented (+4.8 ± 16.5%) s-LPA from inducing a reduction in pGABA_Aγ2_:GABA_Aγ2_ ratio (Fig 6G). Expression of GABA_Aγ2_ remained unchanged regardless of treatment (Fig 6G). s-LPA also had no effect on the GABA-evoked currents in SMNs pretreated with Cap for 30 min (Cap: 2.2 ± 0.3 nA; Cap+s-LPA: 2.1 ± 0.3 nA; *n* = 5 SMNs) (Fig 6H). s-LPA-induced alterations in mIPSCs_GABAA_ and eIPSCs_GABAA_ in HMNs were also CaN-dependent (S1 Text; S15 Fig). Additionally, CaN activity strongly increased in SMNs after incubation with s-LPA, but not with s-LPA plus H1152 or H1152 alone (Fig 6I). Altogether, these data show that (s-)LPA, specifically acting through postsynaptic LPA_1_-RhoA/ROCK-CaN signaling pathway, regulate GABA_A_R-mediated neurotransmission, by a mechanism involving dephosphorylation of GABA_Aγ2_ subunit at Ser^327^.

### LPA Induces Internalization of GABA_Aγ2_ Subunit

**Fig 7:**
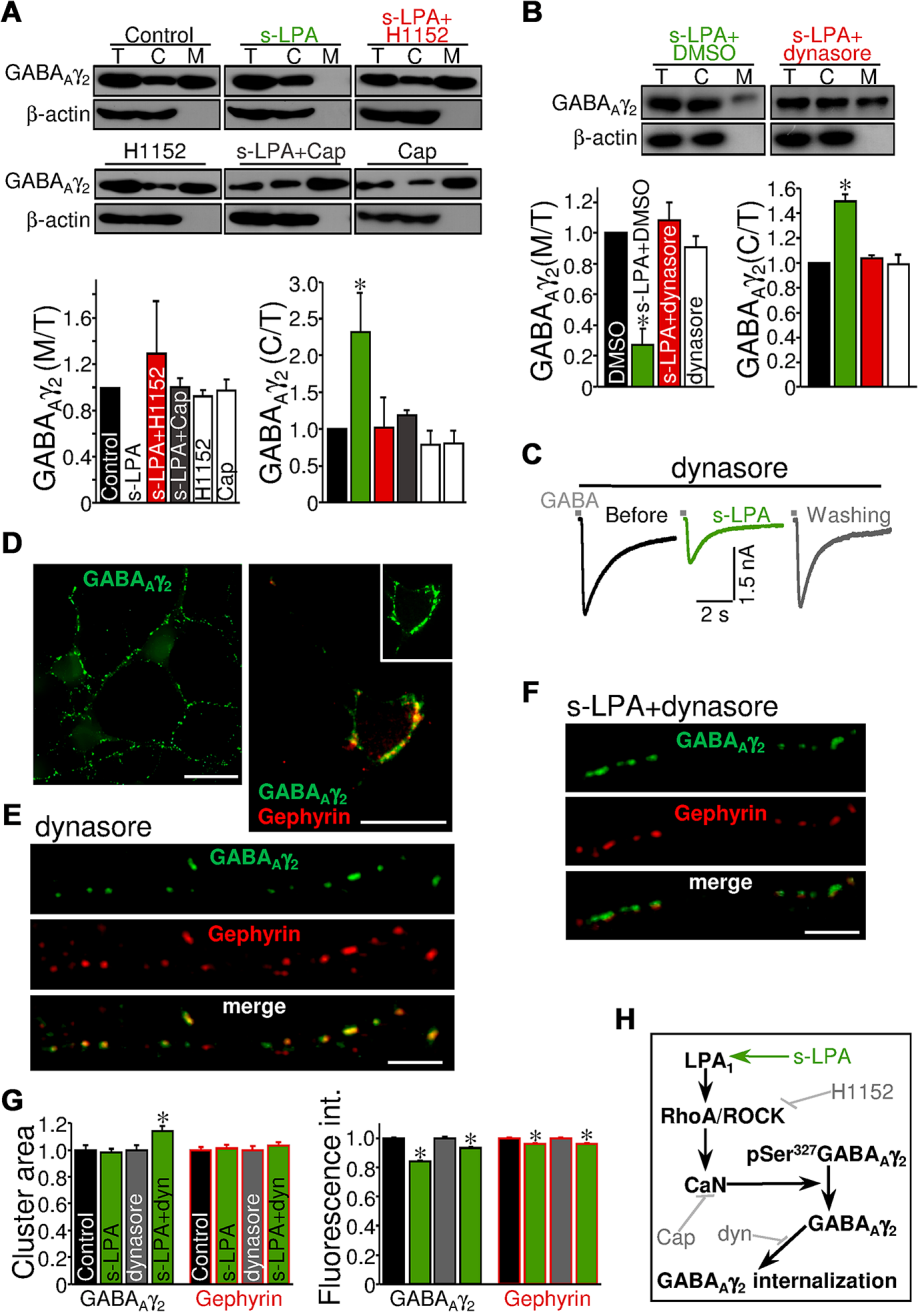
LPA induces dephosphorylation and internalization of the GABA_Aγ2_ subunit in a ROCK/CaN-dependent manner. (A, B) Western blot (top) and averaged ratio (bottom) of total (T), cytosolic (C), and membrane-associated (M) GABA_**Aγ2**_ in cultured SMNs incubated (10 min) with aCSF alone (control) or supplemented with indicated drugs (A). Dynasore (80 μM) or vehicle (0.2% DMSO) were added to the incubation solution 30 min before subsequent s-LPA coaddition for 10 min (B). β-actin was an internal loading reference for T and C fractions and an indicator for fractionation purity. The average densitometric signals for the GABA_**Aγ2**_ C and M samples were expressed as a fraction of T GABA_**Aγ2**_ of the same samples and normalized to the corresponding ratio determined for samples representing control conditions. *p < 0.05, one-way ANOVA on Ranks relative to control or vehicle condition. (C) Same as in Fig 6C under indicated treatments. Treatment with dynasore began at least 30 min before patch performance and was present all along the recording protocol. (D) Left, low-magnification photomicrographs showing a group of SMNs at 6 days in vitro treated for 40 min with aCSF alone and stained for GABA_**Aγ2**_. Right, detail of a SMN exemplifying close association between GABA_**Aγ2**_- and gephyrin-ir clusters. (E, F) Examples of GABA_**Aγ2**_- and gephyrin-ir clusters in the surface of neurites obtained from SMNs treated for 40 min with dynasore (E) or 30 min with dynasore plus 10 min with s-LPA+dynasore (F). Scale bars: D, 50 μm; E, F, 5 μm. (G) Normalized mean cluster area (left) and fluorescence intensity (right) of GABA_**Aγ2**_- and gephyrin-ir clusters analyzed under the indicated treatments (n > 1,200 clusters per condition). *p < 0.005, Student’s *t* test relative to control or dynasore condition. (H) Diagram of the proposed pathway mediating LPA-induced STD on GABA_**A**_R-mediated neurotransmission. Drug targets are also indicated. Plots data can be found in S1 Data.

It is generally accepted that dephosphorylation appears to be important for receptor endocytosis [4,9]. As a next step, we investigated whether LPA-triggered dephosphorylation was accompanied by further subunit internalization. We found that s-LPA (15 min) led to a strong reduction (−99.9 ± 0.01%) in the amount of GABA_Aγ2_ allocated in M fraction in SMN cultures. A proportional increase (+109.4 ± 14.1%) in the quantity of GABA_Aγ2_ was observed in the C fraction relative to total GABA_Aγ2_ (Fig 7A). These outcomes suggest a translocation of at least this subunit from the SMN membrane to the cytosol triggered by s-LPA. The s-LPA-induced translocation was prevented by coincubation with either the ROCK inhibitor H1152 or the CaN inhibitor Cap (Fig 7A). GABA_Aγ2_ compartmentalization in SMNs was maintained after treatment with H1152 or Cap per se (Fig 7A).

To explore whether internalization is actually required for LPA-induced GABA_A_ergic STD, and given that GABA_A_R endocytosis is dynamin-dependent [39], we added the dynamin inhibitor dynasore to the bath to block GABA_A_R endocytosis. Dynasore (80 μM for 30 min) fully prevented both a reduction in the GABA_Aγ2_ M:T ratio and an increase in the C:T ratio induced by s-LPA, which was not altered by vehicle (−84.5 ± 5.8%). Dynasore per se did not modify GABA_Aγ2_ location (−6.4 ± 18.8%) relative to the vehicle condition (100.0 ± 36.7%) (Fig 7B). Interestingly, electrophysiological recordings showed that preincubation with dynasore had no effect on s-LPA-induced changes in GABA-evoked currents (−48.1 ± 8.7%; *n* = 4 SMNs) (Fig 7C). These outcomes support that GABA_Aγ2_ internalization by endocytosis is not required for the attenuation in GABA_A_ergic neurotransmission induced by LPA signaling.

CaN-dependent dephosphorylation of Ser^327^ at the GABA_Aγ2_ subunit is involved in the increase of lateral diffusion and cluster dispersal of surface GABA_A_Rs in the dendrites of cultured hippocampal neurons [36,40]. Therefore, we investigated whether s-LPA-induced STD under endocytosis inhibition conditions would involve GABA_A_R cluster disarrangement. Double immunolabelling for GABA_Aγ2_ and the postsynaptic scaffolding protein, gephyrin, confirmed GABA_Aγ2_-ir clusters at the surface of SMNs, most of them colocalized with gephyrin-ir clusters (Fig 7D). In consonance with phospholipid-evoked GABA_A_R internalization, treatment with s-LPA (10 min) reduced mean fluorescence intensity, but not area, per cluster for these two postsynaptic proteins (Fig 7E–7G). However, the size of surface GABA_Aγ2_-ir clusters increased in parallel with a reduction in fluorescence when s-LPA was added after pretreatment with dynasore (Fig 7E–7G). This agrees with s-LPA-induced lateral diffusion and cluster dispersal of GABA_A_Rs. In addition, the mean area of GABA_Aγ2_-associated clusters of gephyrin was unaltered, but fluorescence was reduced by s-LPA under endocytosis inhibition (Fig 7E–7G). These results are compatible with s-LPA-induced disorganization of GABA_A_R clusters that concludes in receptor internalization. Effects under the presence of dynasore support that this GABA_A_R disarrangement might involve previous lateral diffusion and cluster dispersal of surface GABA_A_Rs like that reported previously for cultured hippocampal neurons [36,40].

In summary, our data highlight a pathway by which, via recruitment of RhoA/ROCK signaling, postsynaptic LPA_1_ evokes CaN-dependent dephosphorylation at Ser^327^ of the GABA_Aγ2_ subunit, which is followed by GABA_A_R cluster dispersion and its concomitant translocation from the plasma membrane to the cytosol (Fig 7H). The latter does not seem to be required for the reduction in GABA_A_ergic synaptic strength triggered by LPA. Phospholipid-induced synaptic strength depression seems to be mainly supported by GABA_Aγ2_ dephosphorylation and subsequent GABA_A_R cluster dispersal.

### LPA_1_ Is Essential for Activity-Dependent Synaptic Plasticity

**Fig 8:**
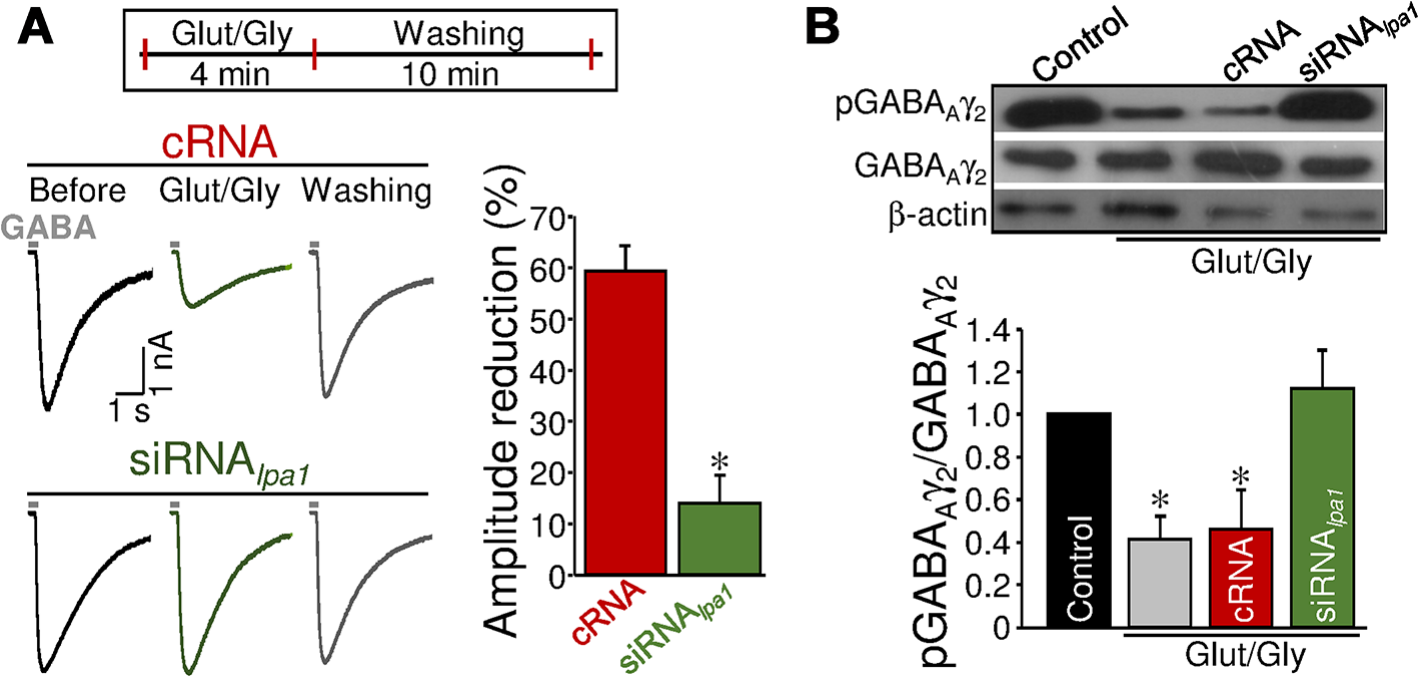
Involvement of LPA_1_ in activity-dependent STD at inhibitory signaling. (A) Same as in Fig 6E, but GABA pulses were performed before and after 4 min addition to the bath of Glut (30 μM) and Gly (1 μM) and after successive washing (cRNA: *n* = 4 SMNs; siRNA_***lpa1***_: *n* = 5 SMNs). *p < 0.05, one-way RM-ANOVA relative to the control (before) condition. (B) Same as in Fig 6G but performed from SMNs cultures receiving indicated pretreatments and incubated for 4 min with aCSF alone or with Glut/Gly to stimulate NMDARs. *p < 0.05, one-way ANOVA on Ranks relative to control (untreated) condition. Experiments were carried out in the presence of TTX, d-tubocurarine, strychnine, and NBQX. Plots data can be found in S1 Data.

Next, the role of LPA signaling in short-term, activity-dependent synaptic plasticity was explored. N-methyl-D-aspartate receptor (NMDAR) activation causes a rapid, local, surface dispersal of synaptic GABA_A_Rs leading to an inhibitory synaptic depression [36,37]. We directly examined the role of LPA_1_-mediated signaling in NMDAR-induced STD of GABA_A_ergic signaling in SMNs. In cRNA-treated SMNs, perfusion of glutamate and glycine (Glut/Gly) for 4 min caused a rapid and reversible depression in GABA-induced current (−59.6 ± 5.3%, *p* < 0.001) in the presence of TTX, d-tubocurarine, strychnine and NBQX. This activity-dependent plastic event was absent in SMNs precultured with siRNA_*lpa1*_ (−15.2 ± 8.7%; Fig 8A), in untreated cells under zero extracellular Ca^2+^ (−9.3 ± 11.5%; *n* = 6 SMNs), or in the presence of APV (−5.4 ± 13.9%; *n* = 6 SMNs), demonstrating Ca^2+^- and NMDAR-dependence. LPA_1_ knockdown reduced by approximately 40% the magnitude of activity-dependent STD at inhibitory synapses. From an extrapolation of these values to the dose-response curve in Fig 2C, it could be indirectly estimated that local concentrations of phospholipids achieved in response to those levels of motoneuron activity were first order micromolar, assuming all synthesized and released phospholipids were the monounsaturated form of LPA (18:1).

Glut/Gly also caused a drastic decrease in the pGABA_Aγ2_:GABA_Aγ2_ ratio in untreated or cRNA-incubated SMNs, which was prevented by siRNA_*lpa1*_ (Fig 8B). Altogether, these data indicate that NMDAR-driven GABA-current depression was spike-independent and essential to extracellular Ca^2+^ entry via NMDARs and LPA_1_ activation, which downstream induces Ser^327^GABA_Aγ2_ dephosphorylation.

Findings from activity-dependent synaptic plasticity experiments agree with the notion that motoneurons are potential sources for Ca^2+^-dependent, spike-independent synthesis and release of lysophospholipids, which in turn might stimulate autocrine signaling pathways (to modulate inhibitory synapses), at least by way of the LPA_1_ receptor. These outcomes also strongly point to lysophospholipids as paracrine retrograde messengers that act on presynaptic LPA_1_ to regulate excitatory synapses; however, further research is needed to confirm this possibility.

### Endogenous LPA Signaling Restrains Baseline Activity of Motoneurons in Adulthood

Finally, physiological involvement of LPA signaling in performance of motor output commands was investigated. In vivo, most HMNs exhibit rhythmic inspiratory-related bursting discharges driven by glutamatergic brain stem afferents, mainly acting on AMPARs, with little or no contribution of inhibitory inputs [22,23].

**Fig 9:**
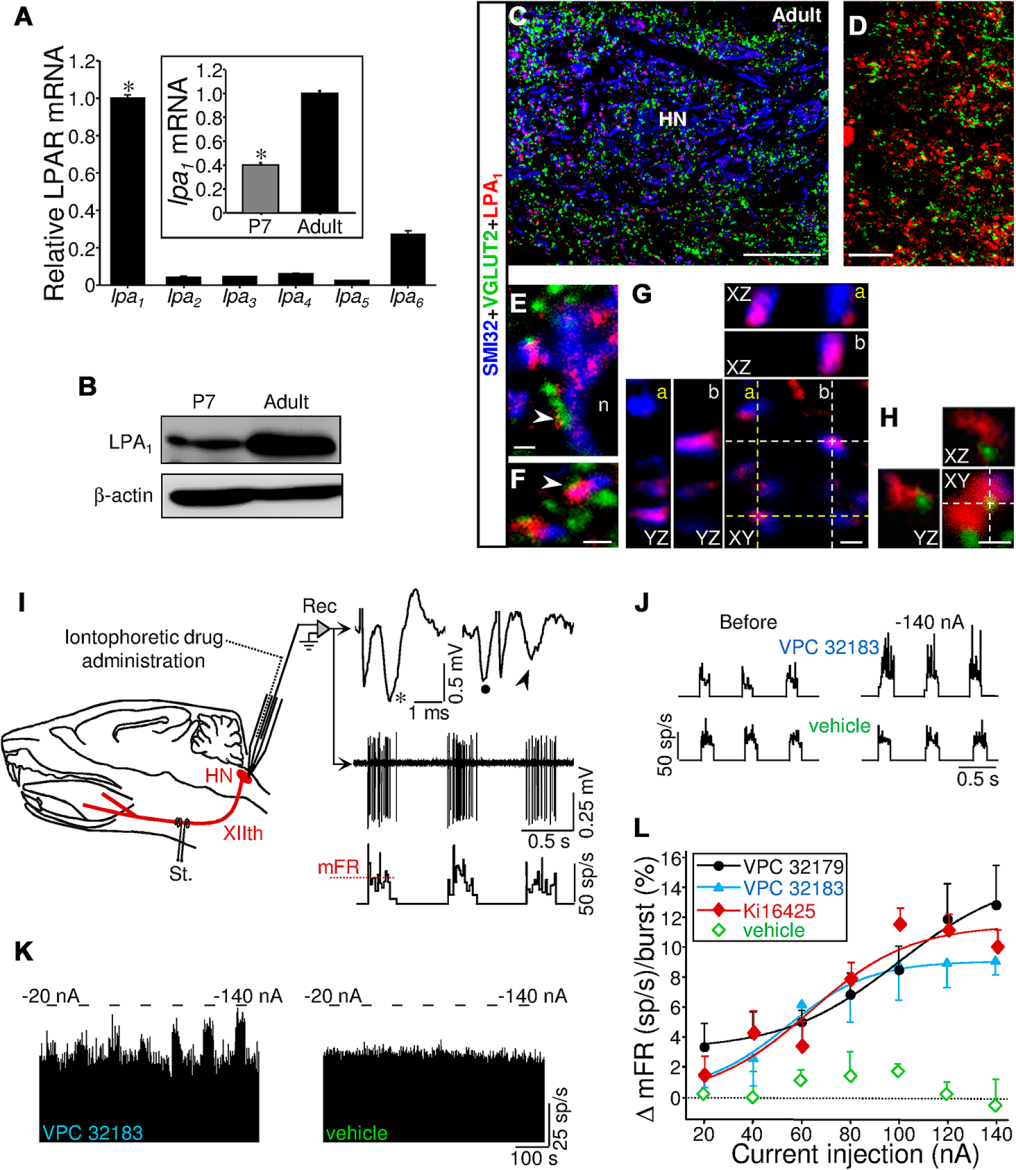
Endogenous LPA signaling restrains inspiratory-related baseline activity of HMNs in the adult rat. (A) Like Fig 1A, but tissue was extracted from adult rats. Inset, comparative expression levels of *lpa*_***1***_ mRNA in HNs from neonatal (P7) and adult rats. *p < 0.05, one-way ANOVA on Ranks relative to *lpa*_***2–6***_ or the adult condition in the inset. (B) Western blot for LPA_**1**_ in the HN at P7 and at the adult age. β-actin was an internal loading reference. (C) Low-magnification confocal image taken from a selected region of the HN in an adult rat showing triple immunolabeling for SMI32, VGLUT2, and LPA_**1**_. (D) Double immunolabeling noticed LPA_**1**_ colocalizing with excitatory terminals (yellow) in the HN. (E, F) Confocal *xy*-planes showing immune LPA_**1**_ staining colocalizing with excitatory terminals (yellow, arrowheads) and SMI32-ir structures (purple). Note in E a VGLUT2-immunopositive input colocalizing with LPA_**1**_ that is apposed to the soma of a LPA_**1**_-expressing HMN (n, HMN nucleus). (G, H) Images of LPA_**1**_ and SMI32 (G) or VGLUT2 (H) are shown in the *xy*-, *xz*-, and *yz*-planes illustrating 3-D reconstructions. The white and yellow crosshairs display locations of *xz*- and *yz*-planes. Note that LPA_**1**_-ir colocalizes and borders SMI32-ir structures (E–G), supporting cytosolic and membrane localization of the LPAR in HMNs. It also colocalizes with VGLUT2 (E, F, and H), indicating its expression in excitatory terminals. Scale bars: C, 50 μm; D, 20 μm; E–H, 2 μm. (I) Schematic diagram of the in vivo experimental preparation. Unitary discharge activity (Rec) of HMNs was obtained in decerebrated, vagotomized, and artificially ventilated adult rats, which had been injected with a neuromuscular blocking agent. A three-barreled pipette with a barrel for electrophysiological recordings and another for microiontophoretic administration of a drug are illustrated. HMNs were identified by their antidromic activation from the electrode (St.) implanted in the XIIth nerve and by the collision test (top traces) between spontaneous orthodromic (dot) and antidromic (asterisk) evoked action potentials. When the stimulus was triggered by a spontaneous spike at a short latency, the antidromic action potential was occluded (arrowhead). Middle and bottom traces represent the extracellularly recorded spike discharge for an inspiratory HMN and the histogram of the instantaneous firing rate (FR, in spikes (sp)/s), respectively. Mean firing rate (mFR, red dotted line) in each burst was measured and subsequently plotted along time. (J) Instantaneous firing rates (sp/s) of two HMNs in response to microiontophoretic administration of VPC 32183 or vehicle (10% DMSO in PBS, pH 8.0) at the indicated current. During the before condition, a retention current of +5 nA is continuously applied. Note the lack of effect of vehicle and the stimulating effect exerted by the application of the LPA_**1/3**_ inhibitor. (K) Time course of the mean FR (mFR, sp/s) per burst in response to microiontophoretic administration (30 s on, 60 s off) of VPC 32183 or vehicle at the indicated applied currents. (L) Mean current-response curves illustrating the effects of microiontophoretically-administered LPA_**1/3**_ antagonists VPC 32179 (0.5 mM; *n* = 7 HMNs), VPC 32183 (1 mM; *n* = 5 HMNs), Ki16425 (2 mM; *n* = 8 HMNs) or vehicle (*n* = 4 HMNs) on motoneuron activity characterized by the change in the mFR per burst. Plots data can be found in S1 Data.

We began by analyzing the level and pattern of expression of the LPA_1_ receptor within the HN of the adult rat. qRT-PCR analysis showed that disparity between *lpa*_*1*_ and *lpa*_*2–6*_ transcripts in the HN was even more accentuated in adults than at the neonatal stage (Fig 9A). Interestingly, mRNA and protein levels for LPA_1_ at adulthood were approximately 150% and 140%, respectively, higher than in neonatal animals (Fig 9A and 9B). These results suggest a gain in relevance of LPA_1_-mediated signaling in the HN during postnatal development, supporting previous observations in the murine brain [41]. Immunohistochemistry revealed LPA_1_-ir puncta-like structures all along the HN (Fig 9C) and colocalization between VGLUT2- and LPA_1_-ir puncta (Fig 9D, 9E, and 9H). A high proportion of VGLUT2-ir inputs (47.9 ± 3.4%; *n* = 55 HMNs) apposed to the perikarya of SMI32-identified HMNs were colocalizing with LPA_1_-ir puncta (Fig 9E and 9F). This also supposed an increase of approximately 150% during postnatal maturation. LPA_1_-ir appeared to border and colocalize with SMI32-ir structures (Fig 9G), supporting cytoplasmic and membrane location of LPA_1_ in adult HMNs. Therefore, the molecular machinery to support a role of LPA_1_ in modulating excitatory neurotransmission is also present in adults.

Additionally, in vivo decerebrated rats maintain respiratory activity [22,23]. To look for a role of LPA signaling in processing motoneuron inspiratory activity, LPA_1/3_ inhibitors VPC 32179 (0.5 mM), VPC 32183 (1 mM), and Ki16425 (2 mM) or its vehicle (10% DMSO) were microiontophoretically applied to antidromically-identified HMNs subjected to unitary extracellular recordings (Fig 9I). The effect of these drugs on the unitary basal firing inspiratory-related activity of HMNs in basal conditions (end-tidal CO_2_ = 4.8%–5.2%) was evaluated. The time course of the mean firing rate averaged over the duration of the inspiratory burst (mFR/burst) was measured by applying increasing currents (−20 to −140 nA, 30 s duration) through the drug barrels (Fig 9J–9L). A current-dependent increase in the mFR/burst of HMNs was observed for all drugs but not when current was applied to the vehicle solution (Fig 9J–9L). In summary, these data point to a physiological role for LPA signaling in motor output performance by restraining the inspiratory-related activity driven by glutamatergic inputs to HMNs.

## Discussion

The present study showed that bioactive membrane-derived phospholipids evoke rapid and reversible synaptic depression and mediate activity-dependent synaptic plasticity, mainly via LPA_1_. Phospholipids likely operate as local messengers in activity-dependent GABAergic STD in a Ca^2+^-dependent, spike-independent manner. Strikingly, at physiological concentrations of nanomolar to first order micromolar, LPA has a greater effect on inhibitory than excitatory inputs. Finally, LPA signaling regulates brain-elemental processing tasks such as performance of motor output commands. These data open a new scenario in which the membrane-phospholipid metabolism actively participates in controlling synaptic strength, and then affects neuronal excitability in physiological and pathological states.

**Fig 10:**
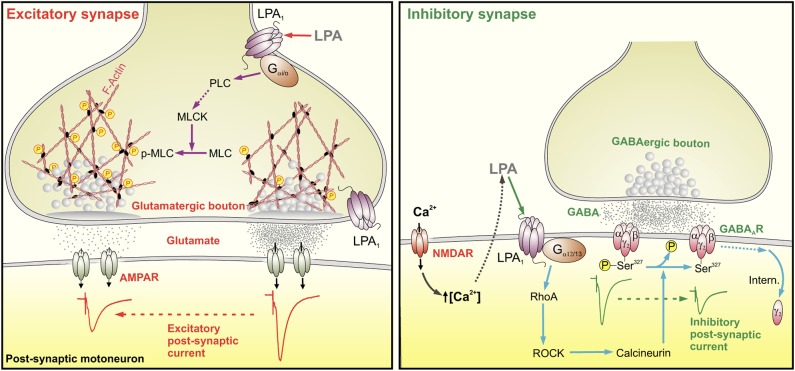
Proposed mechanism by which a membrane-derived bioactive phospholipid such as LPA regulates synaptic strength. LPA affects main excitatory and inhibitory synapses by different degrees, loci, and mechanisms of action. At glutamatergic synapses (left diagram), binding of phospholipids to presynaptic G_**αi/o**_-protein-coupled LPA_**1**_ indirectly activates MLCK via PLC, promoting MLC phosphorylation and subsequent actomyosin cytoskeleton contraction. This would alter the spatial distribution of SVs within the presynaptic terminal and the RRP size of SVs, which results in rapid and reversible excitatory STD. At the GABAergic synapse (right diagram), LPA—synthesized and/or released in response to Ca^2+^ entry through NMDAR—interacts with postsynaptic G_**α12/13**_-coupled LPA_**1**_, then activates RhoA/ROCK. Subsequently, phosphatase calcineurin acts to dephosphorylate Ser^327^ of the GABA_**Aγ2**_ subunit, which in turn undergoes lateral diffusion and internalization by endocytosis. Dephosphorylation of the GABA_**Aγ2**_ component of GABA_**A**_Rs results in rapid and reversible inhibitory STD.

Important determinants of synaptic strength, short-term plasticity and intersynaptic crosstalk mainly involve fine-tuning of the number of neurotransmitter receptors and the RRP size of SVs [4,8]. LPA depresses the main excitatory and inhibitory synaptic systems, affecting both by different degrees, loci, and mechanisms of action. At glutamatergic synapses, and by way of presynaptic G_αi/o_-protein-coupled LPA_1_ and PLC-MLCK activation, LPA results in MLC phosphorylation, which might stimulate the actomyosin contractile apparatus [32] to reduce the bulk of the RRP of SVs (Fig 10). Depletion of some RRP of SVs usually underlies short-term forms of synaptic depression [1,2]. Ultrastructural correlates for LPA-induced STD further supported that functional synaptic changes are partly explained by a reduction in the size of the RRP of SVs. Changes in the actin cytoskeleton are a prerequisite for exocytosis, enabling docking and fusion of SVs with the plasmalemma [32]. As in our results, LPA-dependent contraction of smooth muscle cells involves activation of PLC and MLCK, followed by MLC phosphorylation [31] that promotes actomyosin interactions [32]. In this context, a physical relationship between p-MLC and glutamatergic synapses on adult and neonatal motoneurons has been recently reported [42]. At the calyx of Held synapse, MLCK controls the size of the fast-releasing pool of SVs [43]. In addition, ROCK regulates p-MLC levels via MLCK inhibition to maintain basal RRP ordering of SVs at excitatory inputs [8,42]. Therefore, presynaptic LPA-dependent and ROCK signaling seem to converge onto a common molecular mechanism, namely MLC phosphorylation and size of the RRP at excitatory synapses. It is interesting, then, that the ROCK inhibitor did not actually enhance LPA-induced depression of AMPAR currents. These outcomes suggest that the antagonistic functional actions of ROCK and LPA_1_-signaling, converging on MLCK, results in a push–pull mechanism that regulates the size of the RRP of SVs at excitatory synapses.

At GABAergic synapses, LPA dephosphorylates Ser^327^ of GABA_Aγ2_ subunits and favors GABA_Aγ2_ internalization via postsynaptic G_α12/13_-coupled LPA_1_/RhoA/ROCK signaling and subsequent CaN activation (Fig 10). The cell surface stability of GABA_A_Rs is regulated by post-translational modifications such as phosphorylation. GABA_A_R phosphorylation is involved in the modulation of receptor biophysical properties and membrane trafficking [44]. Phosphorylation stabilizes the GABA_A_R on the surface and, conversely, dephosphorylation is important for receptor endocytosis [4]. NMDAR activation causes GABA_A_R cluster dispersal and lateral diffusion by CaN activation and dephosphorylation of Ser^327^GABA_A_γ_2_ [36,40], leading to long-term depression at CA1 inhibitory synapses [37]. Dispersal could involve receptor clustering at clathrin-coated sites at the plasmalemma, which invaginate and pinch off to form clathrin-coated vesicles. Internalized receptors are then either subject to rapid recycling or are targeted for lysosomal degradation [4].

Our results indicated that the LPA_1_-RhoA/ROCK-CaN pathway dephosphorylates the GABA_A_γ_2_ subunit, which undergoes lateral diffusion, dispersal of clusters, and subsequent endocytosis (Fig 10). However, endocytosis does not seem to be crucial for LPA-induced functional depression at GABA_A_ergic neurotransmission, which seemed to be mainly supported by GABA_A_γ_2_ dephosphorylation and subsequent clusters dispersal of surface GABA_A_Rs. The kinetic recovery suggests rapid replenishment of the synaptic GABA_A_R content, given that re-establishment of inhibitory synaptic strength occurred with 7 to 10 min washing after LPA-induced depression. The coordinated action of kinases and phosphatases, downstream of LPA_1_-triggered signaling, then plays a pivotal role in controlling neuronal excitability by modulation of GABA_A_γ_2_ phosphorylation and receptor recycling.

The present results seem controversial in relation to our previous findings demonstrating a presynaptic role for endogenous baseline ROCK activity in the regulation of AMPAergic and GABA_A_ergic neurotransmission [8]; here, we describe that ROCK also acts postsynaptically to mediate LPA-induced depression of the GABA_A_ergic transmission. Whether presynaptic baseline ROCK activity in inhibitory inputs depends on membrane-derived bioactive lipid mediators, such as LPA and/or sphingosine 1-phosphate, remains to be elucidated. Nevertheless, at glutamatergic synapses, ROCK activity is likely independent of LPA_1/3_ signaling, because inhibitors of these receptors did not mimic AMPAergic STD induced by ROCK inhibition. However, we cannot discard the involvement of another LPAR in maintaining baseline ROCK activity in the synaptic terminals. Interestingly, although presynaptic ROCK is active in our experimental conditions [8], postsynaptic endogenous activity of ROCK, if any, is even below the level required to reveal its impact on synaptic strength and membrane properties [8] of motoneurons. This could be explained by the differential expression of ROCK isoforms at the two compartments, ROCKα in the postsynaptic site and ROCKβ in the presynaptic one, and/or the lower concentration of ROCKα in motoneurons relative to synaptic structures [8]. Anyway, data suggest that when motoneuron activity is low, presynaptic ROCK activity maintains inhibitory synaptic strength by stabilizing the size of the RRP of SVs. However, after exogenous addition of LPA or when motoneuron activity rises, and subsequent coupled LPA synthesis and/or release occurs, postsynaptic LPA_1_ stimulates ROCK. This leads to deinhibition by GABA_Aγ2_ dephosphorylation and receptor endocytosis.

In the rat, the highest LPA concentration in tissue is found in the brain [12]. Cultured cortical neurons produce LPA at nanomolar concentrations [45], but LPA levels increase up to 10 μM after injury, trauma, or hemorrhage involving blood–brain barrier damage [46]. Here, physiological concentrations (nanomolar to first order micromolar) of LPA affected GABAergic to a greater degree than glutamatergic inputs, achieving maximal and similar affectation at 10 μM. Thus, it is possible that LPA signaling maintains neuronal excitability around a dynamic range, promoting deinhibition at low levels of neuronal activity and depressing excitatory inputs when activity increases, perhaps as part of a homeostatic mechanism that prevents excitotoxicity. Any candidate for coupling synaptic strength to neuronal activity must be regulated by activity at the postsynaptic site. Interestingly, noxious stimulation of primary afferent neurons induces LPA production in the dorsal horn in a glutamate-dependent manner [21]. Here, LPA signaling, mainly via LPA_1_, was essential in STD of inhibitory inputs triggered by precedent activity of the neuron. Autocrine LPA signaling was essential for NMDAR-driven GABA-current depression, which depends on extracellular Ca^2+^ entry passing through NMDARs. Activity-dependent synaptic plasticity occurred independently of the generation of action potentials at the postsynaptic neuron. Postsynaptic [Ca^2+^] increase and LPA signaling dependence for activity-dependent STD in cultured motoneurons strongly support that this cell type is a potential source for activity-dependent LPA synthesis and/or release.

Despite the apparent lack of endogenous LPA signaling affecting synaptic strength in our in vitro model, local iontophoretic application of three LPA_1/3_ inhibitors increased, in a dose-dependent manner, the baseline inspiratory-related activity of HMNs in the adult rat. This rhythmic inspiratory-related bursting discharge of HMNs is driven mainly by glutamatergic brain stem afferences, with little or no contribution of inhibitory inputs [22,47]. There is an apparent gain in relevance of LPA_1_-mediated signaling in the HN during postnatal development, to the detriment of LPA_2–6_-triggered pathways, as well as excitatory inputs apposed to adult HMNs express LPA_1_. Taken together, these findings support that phospholipids, most likely activating LPA_1_ at glutamatergic synapses, controlled physiological inspiratory-related activity of HMNs, presumably by restraining their AMPAergic input drive [22]. Thus, endogenous LPA signaling physiologically contributes in the performance of normal patterns of motor output commands in adult animals.

Alterations in phospholipid homeostasis affect various pathological conditions, thus attracting increased diagnostic and pharmacological interest [48]. The exquisite balance between excitatory and inhibitory inputs is critical for the proper functioning of the brain, and its imbalance leads to the cognitive impairment associated with neurodegenerative diseases and metabolic syndromes related to obesity, dyslipidemia, lipodystrophy, insulin resistance, and alcoholism [49–51]. In particular, LPA production and/or autotaxin are increased in obesity-associated metabolic diseases [52], induced hypercholesterolemia [53], congenital lipodystrophy [54], as well as in ethanol-fed mice [55] and in patients with Alzheimer disease [56] or multiple sclerosis [57]. In addition, phospholipids uptake in mammalian cells depends on their activation status, a critical support for cellular incorporation of nutrition-derived fatty acids. Imported phospholipids are utilized for production of bioactive lipids, such as LPA [58], and thereby modify synaptic transmission. Therefore, we can point to LPA as a promising candidate in coupling brain function, by modulating synaptic strength and plasticity, to the metabolic condition of the organism across physiological and pathological states.

## Materials and Methods

Wistar rats of either sex and CD1 pregnant mice were obtained from an authorized supplier (Animal Supply Services, University of Cádiz, Spain), and were cared for and handled in accordance with the guidelines of the European Union Council (86/609/UE) and Spanish regulations (BOE 67/8509-12; BOE 1201/2005) on the use of laboratory animals. Animals were individually housed—except neonatal animals, which were housed with their mother—in cages with water and food pellets available ad libitum, under temperature-controlled conditions at 21 ± 1°C, with a 12 h light and dark cycle. Efforts were made to minimize the number of animals used and their suffering. All surgical procedures were carried out under aseptic conditions. Experimental procedures were approved by the local Animal Care and Ethics Committee.

### Electrophysiological Recordings

#### In vitro whole-cell patch-clamp recordings of motoneurons

Whole-cell patch-clamp experiments were performed on cultured SMNs or on HMNs from transverse brain stem slices (300–400 μm thick) of P6–P9 rats as previously described [8,42,59]. Whole-cell AMPAergic responses were recorded at a holding potential of −65 mV with the KGluconate-based intracellular solution. GABA_A_ postsynaptic currents were recorded in cells voltage-clamped at −75 mV using the CsCl-based electrode solution. The AMPAergic or GABA_A_ergic component of the evoked currents was pharmacologically isolated as indicated in the legend of Fig 2B.

#### Unitary extracellular recordings of HMNs in the adult rat

Adult animals (250–300 gr) were prepared for extracellular recordings as reported previously [60,61]. Tracheotomized, vagotomized, and decerebrated animals were paralyzed and mechanically ventilated. End-tidal CO_2_ was kept at 4.8%–5.2% along the recording session. Three-barreled, microfilament-filled glass pipettes were used for single-unit recording and microiontophoretic drug administration.

### Immunohistochemistry

Brain stem coronal sections (30 μm thick) and SMNs were processed by immunohistochemistry against vesicular glutamate (VGLUT2), GABA (VGAT) transporters, GABA_Aγ2_ subunit, gephyrin and/or Munc13-1 as synapse-related markers, LPA_1_, and/or the nonphosphorylated form of neurofilament H (SMI32) as a motoneuron marker, following standard protocols.

### Electron Microscopy

Brain stem slices (300 μm thick) incubated for 10 min (approximately 22°C), with aCSF alone, 0.2% DMSO (vehicle) or with various drug treatments were immediately fixed and processed for electron microscopy analysis. Ultrathin sections (70–80 nm thick) were analyzed at high magnification (43,000x). Only boutons, contacting with motoneurons at the level of the nucleolus, evidencing at least an a.z. were included in this study [8].

### siRNA-Mediated Silencing of *lpa1*

Neonatal rats (P4) received an acute injection of siRNA_*lpa1*,_ or nontargeting siRNA (cRNA), (2 μg/rat) in 2 μl of RNase-free PBS into the fourth ventricle. The target sequence for the siRNA_*lpa1*_ was UCAUUGUGCUUGGUGCCUU. A group of animals was infused with 2 μl of RNase-free PBS (vehicle) as an additional control. Primary cultures of SMNs were incubated with 2.5 μl of either cRNA or siRNA_*lpa1*_ (each 100 μM) for 72 h at 37°C. Cells were then collected for qRT-PCR analyses or used for electrophysiological studies.

### Quantitative Real-Time Reverse Transcriptase PCR (qRT-PCR)

Total RNA was extracted from the HN or cultured SMNs using TRIzol, and 0.5 μg of RNA was used for cDNA synthesis with iScript cDNA synthesis. The PCR primers were as indicated in S2 Table.

### Western Blotting

Total protein was extracted from microdissected HNs, NSC34 cells, and membrane and cytosol fractions of NSC34 cells and SMNs. Membranes were blotted with specific antibodies against GABA_Aγ2_, p^Ser327^GABA_Aγ2_, LPA_1_, p-MLC, MLC, or RhoA. Membranes were also probed with anti-α_1_-tubulin or anti-β-actin antibodies as control for the total amount of protein contained in each well.

### Statistics and Data Analysis

Data are expressed as the mean ± standard error of the mean (SEM). The number of analyzed specimens per experimental condition is indicated in figure legends or in the result section. Data were obtained from at least three animals per experimental condition. In ROCK activity, western blotting and qRT-PCR experiments, each individual assay was performed by using tissue samples collected from at least six animals per experimental condition. Quantitative data from ROCK and CaN activity assays, western blot, and qRT-PCR represent the average of, at least, three independent experiments. Applied statistical tests per experimental condition are indicated in figure legends or in results. Post hoc Holm Sidak or Dunn tests were applied for ANOVA for repeated measures or on Ranks, respectively. In all cases, the minimum significance level was set at *p* < 0.05.

## Supporting Information

S1 Data A dataset file with original data for all figures. (XLSX)

S1 Fig LPA does not act postsynaptically on AMPAergic signaling. (A) Traces of spontaneously occurring mEPSCs_AMPA_ recorded from a representative HMN before and after 10 min bath perfusion with LPA (2.5 μM). mEPSCs_AMPA_ were pharmacologically isolated in the presence of 1 μM tetrodotoxin (TTX), 1 μM strychnine hydrochloride, 30 μM d-tubocurarine, 50 μM (DL)-APV, and 10 μM bicuculline methochloride applied to the bath perfusion. (B) Cumulative probability functions of mEPSC_AMPA_ amplitudes pooled from 4 HMNs recorded under indicated conditions. Bin width: 2 pA. Plot data can be found in S1 Data. (C) Whole-cell AMPAergic currents evoked by 100 ms pressure pulses of glutamate (applied at saturating concentrations; 1 mM) in a HMN before and after superfusion with LPA. Recordings were performed in the presence of TTX in nominally Ca^2+^-free solution. Experiments and analysis were performed as in our previously published study [8].
(TIF)

S2 Fig s-LPA operates presynaptically at AMPAergic synaptic signaling. Top, examples of eEPSCs_AMPA_ recorded in a HMN in response to paired-pulse stimulation of VLRF axons at the indicated conditions. Stimulus interval was 25 ms. The rightmost trace shows the superimposition of the responses scaled to the peak of the first eEPSCs_AMPA_. Bottom, PPR was obtained from the amplitude of the first and second eEPSCs_AMPA_ by the formula eEPSCs_AMPA_2/eEPSCs_AMPA_1. Comparison of PPR measured at interpulse intervals ranging from 25 to 200 ms for HMNs recorded before, during, and after washout of the s-LPA (40 μM; *n* = 6 HMNs). *p < 0.05, two-way RM-ANOVA relative to control (before) condition. Experiments and analysis were performed as in our previously published study [8]. Plots data can be found in S1 Data.
(TIF)

S3 Fig LPA potentiates the facilitation index of eEPSCs_AMPA_ under repeated VLRF stimulation. Top, recorded succession of eEPSCs_AMPA_ in a HMN evoked by a train of 20 stimuli at 40 Hz applied to the VLRF before and after adding LPA. Traces are scaled, with first eEPSCs_AMPA_ of train being equal at both conditions. Bottom, mean eEPSCs_AMPA_ amplitude, normalized to the first eEPSCs_AMPA_ (eEPSCs_AMPA_n/eEPSCs_AMPA_1) plotted against the position number of eEPSCs_AMPA_n within the train (1–20) at the indicated conditions (*n* = 5 HMNs). The symbol code is as in S2 Fig. The stimulus intensity was adjusted so that the eEPSC_AMPA_1 was approximately 50% of the maximal amplitude and then was maintained constant throughout the recording period. *p < 0.05, two-way RM-ANOVA relative to control (before) condition. Plot data can be found in S1 Data.
(TIF)

S4 Fig LPA alters distribution of eEPSCs_AMPA_ amplitude evoked by minimal stimulation. (A) Amplitude distribution histograms of eEPSCs_AMPA_ before and after treatment with LPA. Amplitude of eEPSCs_AMPA_ was distributed over a range from zero to around 115 pA at the before condition; however, LPA narrowed the amplitude distribution toward lower amplitudes (upper limit of approximately 35 pA). Each histogram is made of 800 responses (5 pA bin size) pooled from 4 HMNs. (B) Normalized cumulative probability distributions of eEPSCs_AMPA_ amplitude. Note that LPA displaced to the left the cumulative distribution of eEPSCs_AMPA_ amplitude (*p* < 0.05; Kolmogorov-Smirnov test). Bin width: 2 pA. Failures were excluded. Plots data can be found in S1 Data.
(TIF)

S5 Fig Pharmacological insights for LPA_1_ as a key receptor mediating LPA-induced AMPAergic STD. (A) eEPSCs_AMPA_ from HMNs recorded before and after exposure to OMPT (1 μM) or VPC 32183 (1 μM) alone (top panels) and LPA (2.5 μM) or s-LPA (40 μM) followed by coaddition of VPC 32183 (bottom panels). (B) Mean eEPSCs_AMPA_ amplitude reduction (in percent) at the indicated treatments (*n* ≥ 5 HMNs per condition). *p < 0.05, one-way ANOVA relative to control condition. Plots data can be found in S1 Data.
(TIF)

S6 Fig The LPA_1/3_ inhibitor Ki16425 reverses LPA-induced AMPAergic STD. (A) Top, timing of experimental protocols. HMNs were initially allowed to stabilize (Stabil.) with normal aCSF to obtain baseline control recordings. Slices were then superfused for 10 min with aCSF supplemented with 0.2% DMSO, the LPA_1/3_ inhibitor Ki16425 (0.4 μM in 0.2% DMSO; Drug, left protocol) or LPA (2.5 μM; Drug-1, right protocol) before current responses were acquired again. In the right protocol, slices were additionally incubated for 10 min with LPA plus DMSO or with Ki16425 (0.4 μM; Drug-2). Finally, a last round of acquisition was taken after a 10 min washout with drug-free aCSF. Bottom, representative eEPSCs_AMPA_ from HMNs recorded at the indicated conditions. (B, C) Mean eEPSCs_AMPA_ amplitude (B) and PPR ratio (C) measured at 25 ms interpulse intervals for HMNs recorded under the indicated treatments (*n* ≥ 5 HMNs per condition). *p < 0.05, one-way RM-ANOVA relative to control (before) condition. Plots data can be found in S1 Data.
(TIF)

S7 Fig Effectiveness of siRNA_lpa1_ in knockdown LPA_1_ in the brain stem of neonatal rats. (A) Expression levels of mRNA for indicated LPARs obtained by qRT-PCR of isolated brain stems at P6 after receiving the indicated treatments at P4. GAPDH was used as housekeeping. Values were normalized taking control condition (untreated animals) as 1. *p < 0.05, one-way ANOVA on Ranks relative to control, vehicle, and cRNA conditions for each receptor. (B, C) Immunohistochemistry against LPA_1_ of brain stem coronal hemisections obtained from P6 pups untreated (Control), or receiving the indicated treatments at P4. Scale bar: 500 μm. Plot data can be found in S1 Data.
(TIF)

S8 Fig s-LPA induces AMPAergic STD by a protein G_αi/o_-PLC-dependent mechanism. (A–C) Representative recordings showing the effect of s-LPA (40 μM) on eEPSCs_AMPA_ from 4 HMNs in response to paired-pulse stimulation in the presence of the G_αi/o_ inhibitor PTX (100 ng/ml; A, left), the noncatalytic bPTX (100 ng/ml; A, right), the PLC inhibitor U73122 (1 μM; B), or the G_αq/11_ inhibitor YM-254890 (1 μM; C). Stimulus interval was 25 ms. (D, E) Mean eEPSCs_AMPA_ amplitude reduction (D) and PPR ratio increase (E) measured at 25 ms interpulse intervals for HMNs recorded under the indicated treatments (*n* ≥ 4 HMNs per condition). *p < 0.05, one-way ANOVA relative to control condition. Plots data can be found in S1 Data.
(TIF)

S9 Fig LPA alters distribution of mEPSCs_GABAA_ amplitude in a ROCK-dependent way. Amplitude distribution histograms (A) and cumulative probability functions (B) of mIPSCs_GABAA_ at the indicated conditions. Each condition is represented by 600 events (5 pA bin width) pooled from 5 HMNs. Note that H1152 reversed the LPA-induced shift to the left of the distribution histograms and the cumulative probability functions of mIPSCs_GABAA_ amplitude (*p* < 0.05; Kolmogorov-Smirnov test). Plots data can be found in S1 Data.
(TIF)

S10 Fig Evidence for a non-presynaptic mechanism underlying LPA-ROCK-induced depression of GABA_A_ergic neurotransmission. (A, B) Illustrative eIPSCs_GABAA_ of two HMNs (A) and summary data of eIPSCs_GABAA_ amplitude (B) recorded before and after LPA (2.5 μM; left) or s-LPA (40 μM; right) treatment, after the next coaddition of H1152 (20 μM) and subsequent washing (*n* = 4 HMNs). *p < 0.05, one-way RM-ANOVA relative to the control (before) condition. (C, D) Examples of eIPSCs_GABAA_ recorded in a HMN (C) in response to paired-pulse stimulation of VLRF axons and changes in PPR (D) (*n* = 4 HMNs). Plots data can be found in S1 Data.
(TIF)

S11 Fig SMNs express LPA_1_. (A) Epifluorescence images of cultured SMNs processed by immunohistochemistry for the motoneuron marker SMI32 (left) and counterstained with the nuclear marker DAPI (right). Note that all cells in the field are SMI32-ir. (B) Expression levels of mRNA for the indicated LPARs obtained by qRT-PCR of cultured SMNs relative to the housekeeping GAPDH. *p < 0.05, one-way ANOVA on Ranks relative to *lpa*_*2–6*_. (C) Epifluorescence images of cultured SMNs processed by immunohistochemistry for SMI32 (top) and LPA_1_ (bottom). Scale bars: A, 25 μm; C, 100 μm. Plot data can be found in S1 Data.
(TIF)

S12 Fig (s-)LPA stimulates RhoA/ROCK signaling in motoneurons. (A) Left, western blots of LPA_1_ and total (T), cytosolic (C), and membrane-associated (M) RhoA in the motoneuron-like cell line NSC34 after indicated treatments. For LPA_1_, the cell line HEK293 was taken as a negative control and β-actin expression was used as an internal loading reference. Right, histogram showing the average ratio of densitometric intensity in M or C fractions relative to total RhoA at the indicated conditions. Ratio values were normalized relative to the control group. (B, C) Summary histogram of changes in ROCK activity in homogenates from HN (B) and cultured NSC34 (C) untreated (control) or treated with either s-LPA (40 μM), H1152 (100 μM), or s-LPA plus H1152. *, # *p* < 0.05, one-way ANOVA on Ranks relative to the control and both control and s-LPA-treated groups, respectively. Plots data can be found in S1 Data.
(TIF)

S13 Fig Effectiveness of siRNA_lpa1_ in knockdown LPA_1_ in SMNs. (A) Expression levels of LPAR mRNAs in SMNs after incubation with the small interfering RNA against *lpa*_*1*_ (siRNA_*lpa1*_) relative to cultures treated with a nontargeting siRNA (cRNA). *p < 0.05, one-way ANOVA on Ranks relative to *lpa*_*2–6*_. (B, C) Epifluorescence images of cultured SMNs receiving the indicated treatments processed by immunohistochemistry for SMI32 and LPA_1_. Immunohistochemical processing was performed in parallel. Scale bars: 25 μm. Plot data can be found in S1 Data.
(TIF)

S14 Fig s-LPA induces GABA_A_γ_2_ dephosphorylation in the HN by a ROCK-dependent mechanism. Western blot (top) and averaged ratio (bottom) of phosphorylated and total GABA_A_γ_2_ subunit protein levels (denoted as pGABA_A_γ_2_ and GABA_A_γ_2_, respectively) in the HN of neonatal brain stem slices incubated (10 min) with aCSF alone (control) or supplemented with indicated drugs. β-actin was an internal loading reference. *p < 0.05, one-way ANOVA on Ranks relative to control condition. Plot data can be found in S1 Data.
(TIF)

S15 Fig s-LPA-induced alterations in mIPSCs_GABAA_ and eIPSCs_GABAA_ in HMNs were CaN-dependent. (A–C) Traces of spontaneously occurring mIPSCs_GABAA_ (A), amplitude distribution histograms (B, 5 pA bin size), and cumulative probability functions (C, 5 pA bin size) pooled from 8 HMNs before and after exposure to s-LPA (40 μM). (D) Examples of recorded eIPSCs_GABAA_ in a HMN in response to paired-pulse stimulation of VLRF under the specified treatments. All HMNs were recorded in the presence of CaN autoinhibitory peptide (Cap; 12.5 μM) added to the recording pipette solution. Plots data can be found in S1 Data.
(TIF)

S1 Table Ultrastructural characterization of S-type boutons attached to HMNs. The data used to generate the table can be found in S1 Data.
(DOC)

S2 Table Sequence of primers used for qRT-PCR. (DOC)

S1 Text Supporting results and a more detailed description of materials and methods. (DOC)

## Abbreviations

aCSF: artificial cerebrospinal fluid
AMPAR: AMPA-type glutamate receptor
a.z.: active zone
bPTX: b oligomer of PTX
CaN: calcineurin
Cap: calcineurin auto-inhibitory peptide
CNS: central nervous system
cRNA: control non-interfering siRNA
ePSC: evoked postsynaptic current
eEPSC: evoked excitatory postsynaptic current
eIPSC: evoked inhibitory postsynaptic current
GABA_A_R: A-type GABA receptor
HMN: hypoglossal motoneuron
HN: hypoglossal nucleus
ir: immunoreactive
LPA: lysophosphatidic acid
LPAR: LPA receptor
MAPK: mitogen-activated protein kinase
mIPSC: miniature quantal inhibitory postsynaptic current
MLC: myosin light chain
MLCK: myosin light chain kinase
NMDAR: N-methyl-D-aspartate receptor
PBS: phosphate-buffered saline
PKC: protein kinase C
PLC: phospholipase C
PPR: paired-pulse ratio
PTX: pertussis toxin
ROCK: Rho kinase
RRP: readily releasable pool
siRNA: small interfering RNA
SMN: spinal cord motoneuron
STD: short-term depression
SV: synaptic vesicle
TTX: tetrodotoxin
VGAT: vesicular GABA transporter
VGLUT2: vesicular glutamate transporter 2
VLRF: ventrolateral reticular formation
