# Supplementary figures and images for: Membrane-Derived Phospholipids Control Synaptic Neurotransmission and Plasticity
Source: PLoS Biol. 2015 May 21;13(5):e1002153. doi: 10.1371/journal.pbio.1002153 (PMC4440815; doi:10.1371/journal.pbio.1002153)

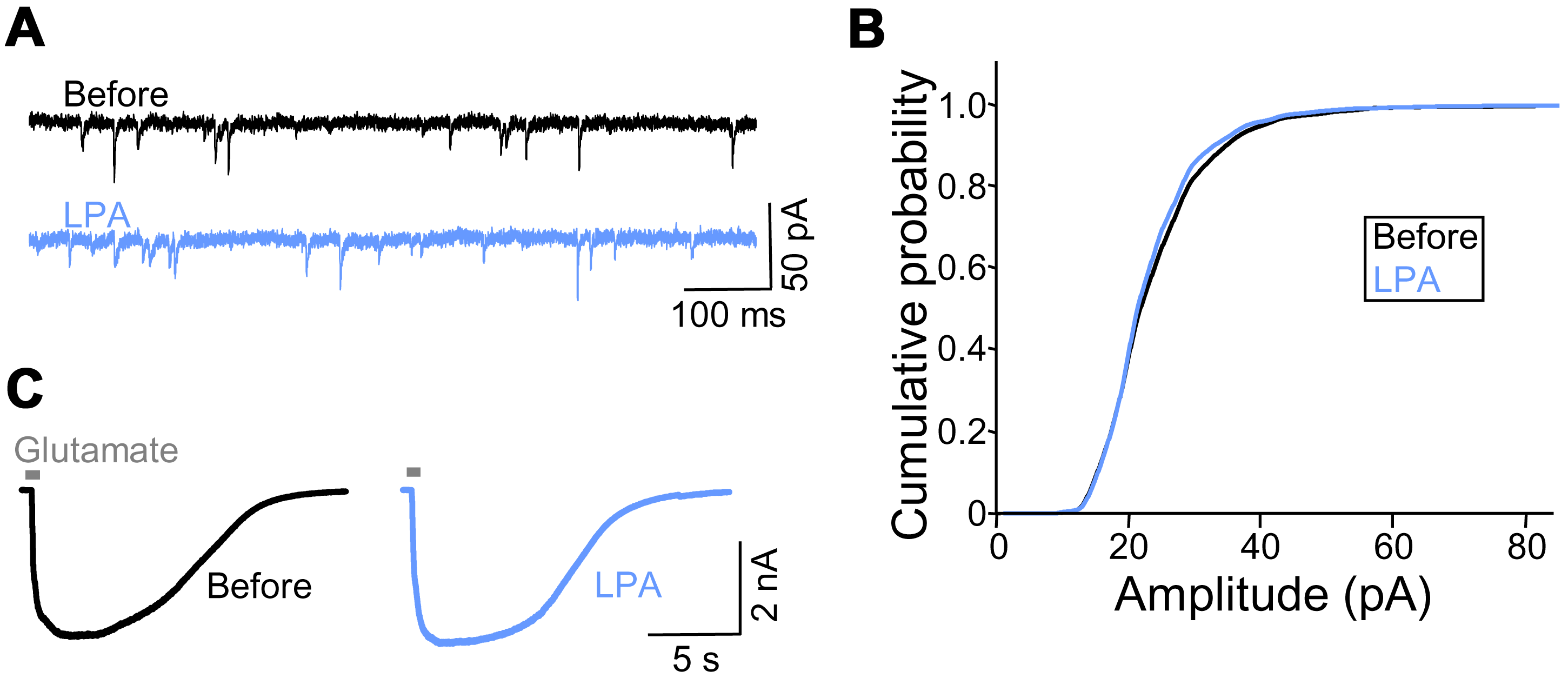

Supplement: S1 Fig — (A) Traces of spontaneously occurring mEPSCsAMPA recorded from a representative HMN before and after 10 min bath perfusion with LPA (2.5 μM). mEPSCsAMPA were pharmacologically isolated in the presence of 1 μM tetrodotoxin (TTX), 1 μM strychnine hydrochloride, 30 μM d-tubocurarine, 50 μM (DL)-APV, and 10 μM bicuculline methochloride applied to the bath perfusion. (B) Cumulative probability functions of mEPSCAMPA amplitudes pooled from 4 HMNs recorded under indicated conditions. Bin width: 2 pA. Plot data can be found in S1 Data. (C) Whole-cell AMPAergic currents evoked by 100 ms pressure pulses of glutamate (applied at saturating concentrations; 1 mM) in a HMN before and after superfusion with LPA. Recordings were performed in the presence of TTX in nominally Ca2+-free solution. Experiments and analysis were performed as in our previously published study [8]. (TIF) [file pbio.1002153.s002.tif]

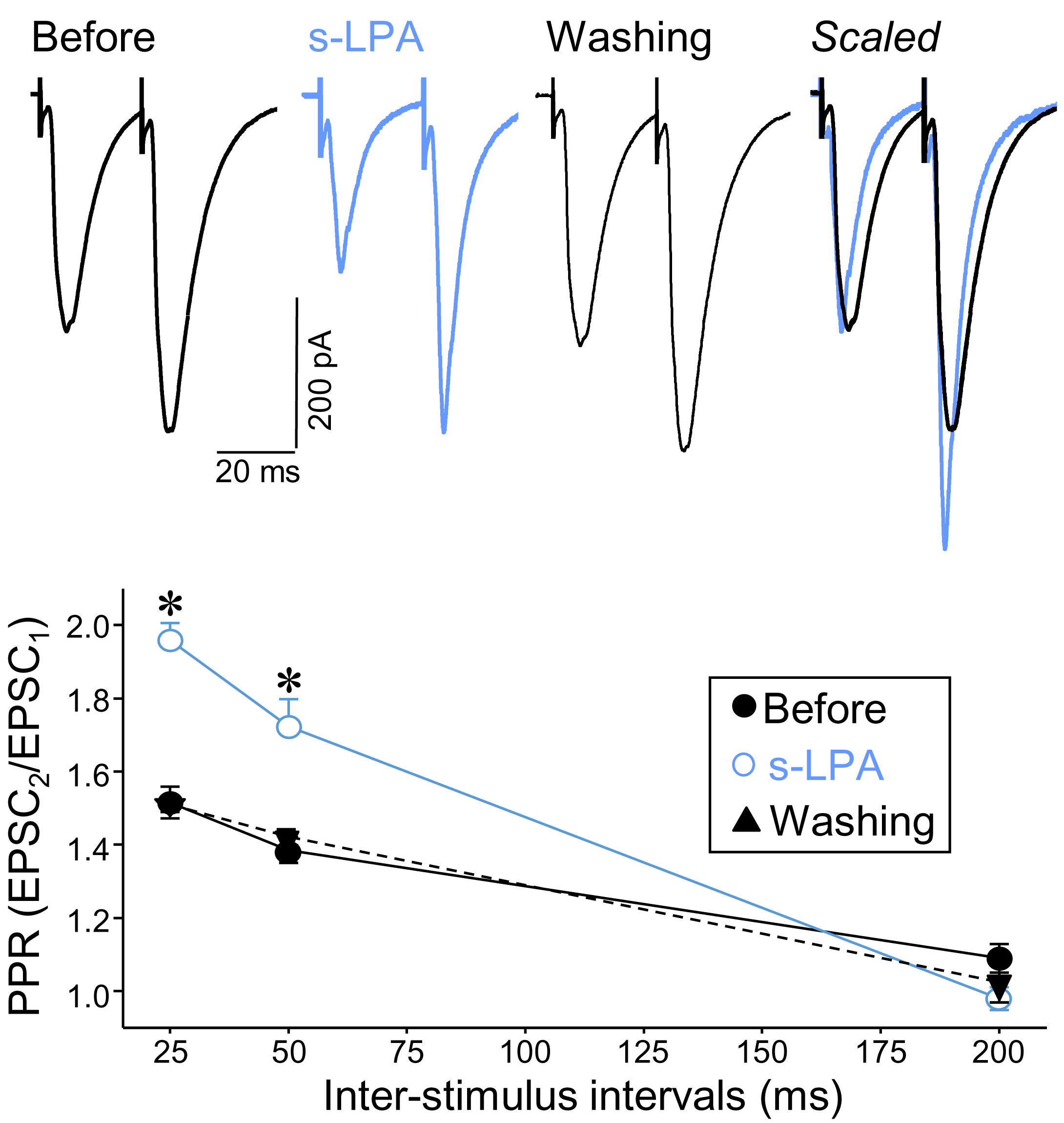

Supplement: S2 Fig — Top, examples of eEPSCsAMPA recorded in a HMN in response to paired-pulse stimulation of VLRF axons at the indicated conditions. Stimulus interval was 25 ms. The rightmost trace shows the superimposition of the responses scaled to the peak of the first eEPSCsAMPA. Bottom, PPR was obtained from the amplitude of the first and second eEPSCsAMPA by the formula eEPSCsAMPA2/eEPSCsAMPA1. Comparison of PPR measured at interpulse intervals ranging from 25 to 200 ms for HMNs recorded before, during, and after washout of the s-LPA (40 μM; n = 6 HMNs). *p < 0.05, two-way RM-ANOVA relative to control (before) condition. Experiments and analysis were performed as in our previously published study [8]. Plots data can be found in S1 Data. (TIF) [file pbio.1002153.s003.tif]

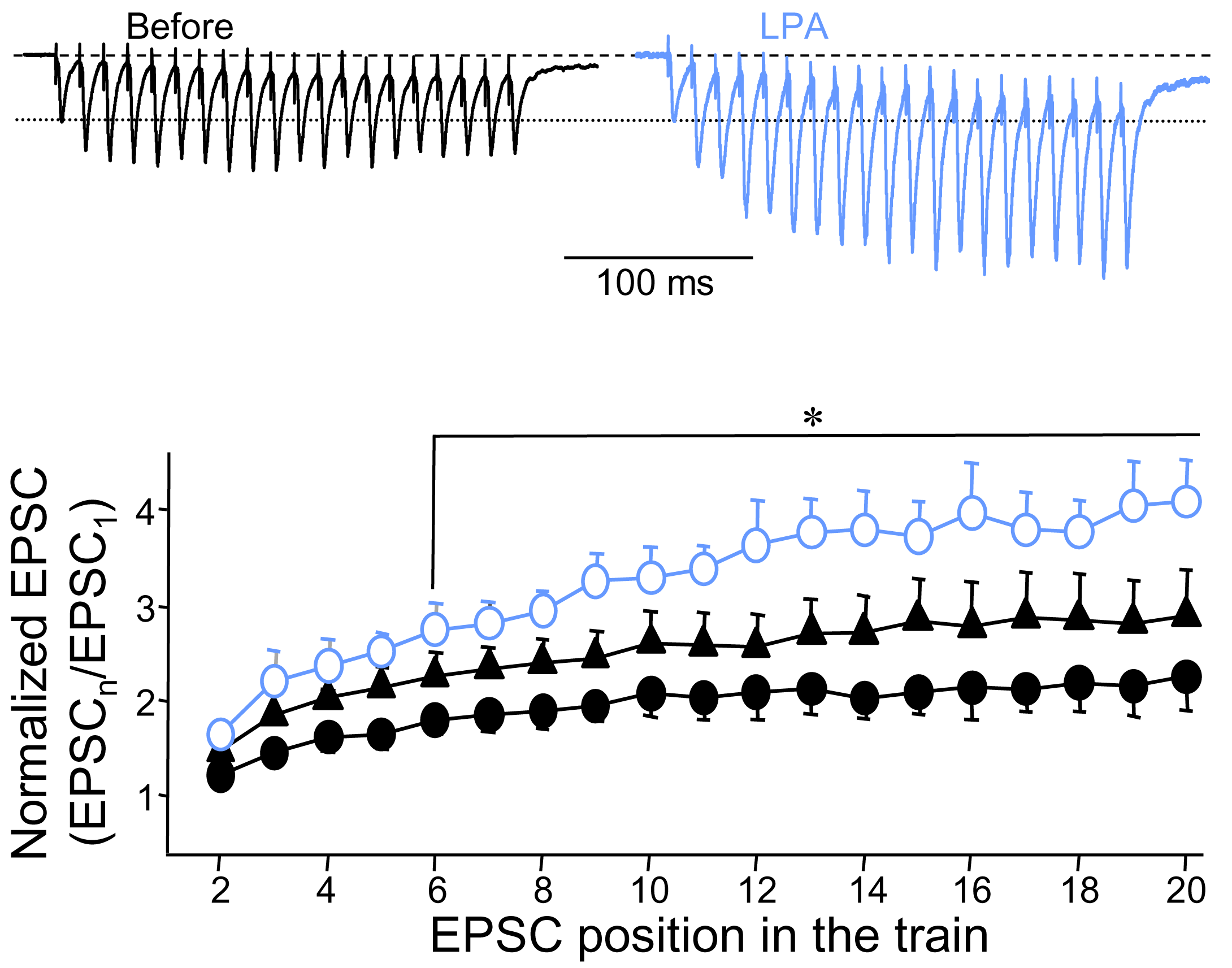

Supplement: S3 Fig — Top, recorded succession of eEPSCsAMPA in a HMN evoked by a train of 20 stimuli at 40 Hz applied to the VLRF before and after adding LPA. Traces are scaled, with first eEPSCsAMPA of train being equal at both conditions. Bottom, mean eEPSCsAMPA amplitude, normalized to the first eEPSCsAMPA (eEPSCsAMPAn/eEPSCsAMPA1) plotted against the position number of eEPSCsAMPAn within the train (1–20) at the indicated conditions (n = 5 HMNs). The symbol code is as in S2 Fig. The stimulus intensity was adjusted so that the eEPSCAMPA1 was approximately 50% of the maximal amplitude and then was maintained constant throughout the recording period. *p < 0.05, two-way RM-ANOVA relative to control (before) condition. Plot data can be found in S1 Data. (TIF) [file pbio.1002153.s004.tif]

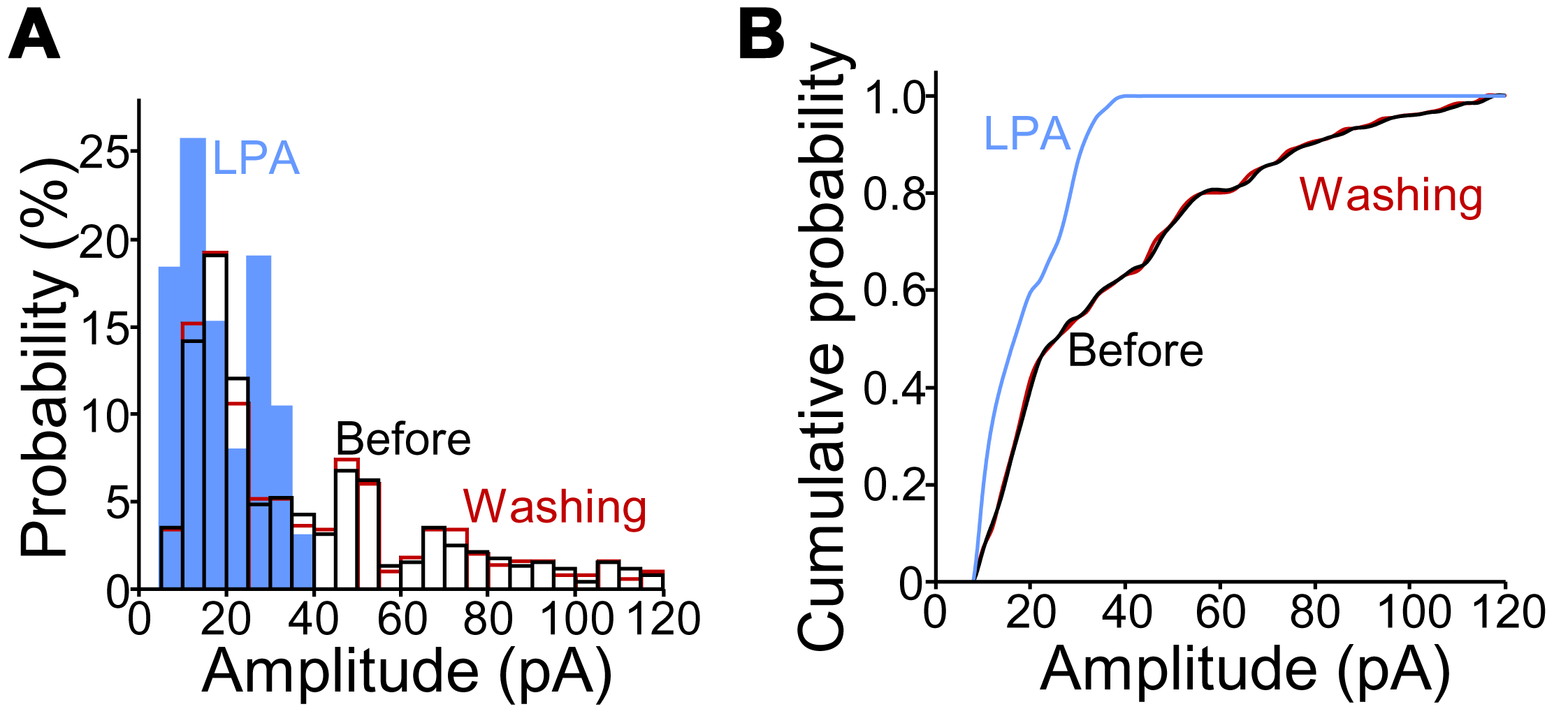

Supplement: S4 Fig — (A) Amplitude distribution histograms of eEPSCsAMPA before and after treatment with LPA. Amplitude of eEPSCsAMPA was distributed over a range from zero to around 115 pA at the before condition; however, LPA narrowed the amplitude distribution toward lower amplitudes (upper limit of approximately 35 pA). Each histogram is made of 800 responses (5 pA bin size) pooled from 4 HMNs. (B) Normalized cumulative probability distributions of eEPSCsAMPA amplitude. Note that LPA displaced to the left the cumulative distribution of eEPSCsAMPA amplitude (p < 0.05; Kolmogorov-Smirnov test). Bin width: 2 pA. Failures were excluded. Plots data can be found in S1 Data. (TIF) [file pbio.1002153.s005.tif]

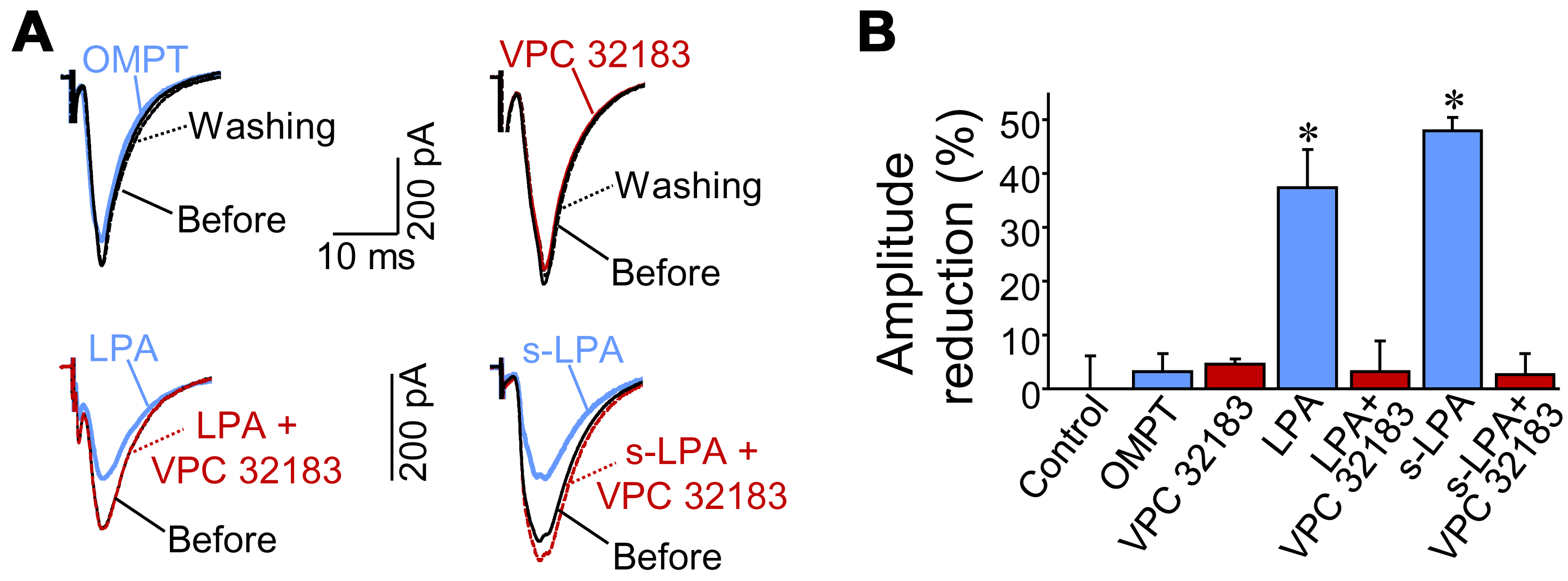

Supplement: S5 Fig — (A) eEPSCsAMPA from HMNs recorded before and after exposure to OMPT (1 μM) or VPC 32183 (1 μM) alone (top panels) and LPA (2.5 μM) or s-LPA (40 μM) followed by coaddition of VPC 32183 (bottom panels). (B) Mean eEPSCsAMPA amplitude reduction (in percent) at the indicated treatments (n ≥ 5 HMNs per condition). *p < 0.05, one-way ANOVA relative to control condition. Plots data can be found in S1 Data. (TIF) [file pbio.1002153.s006.tif]

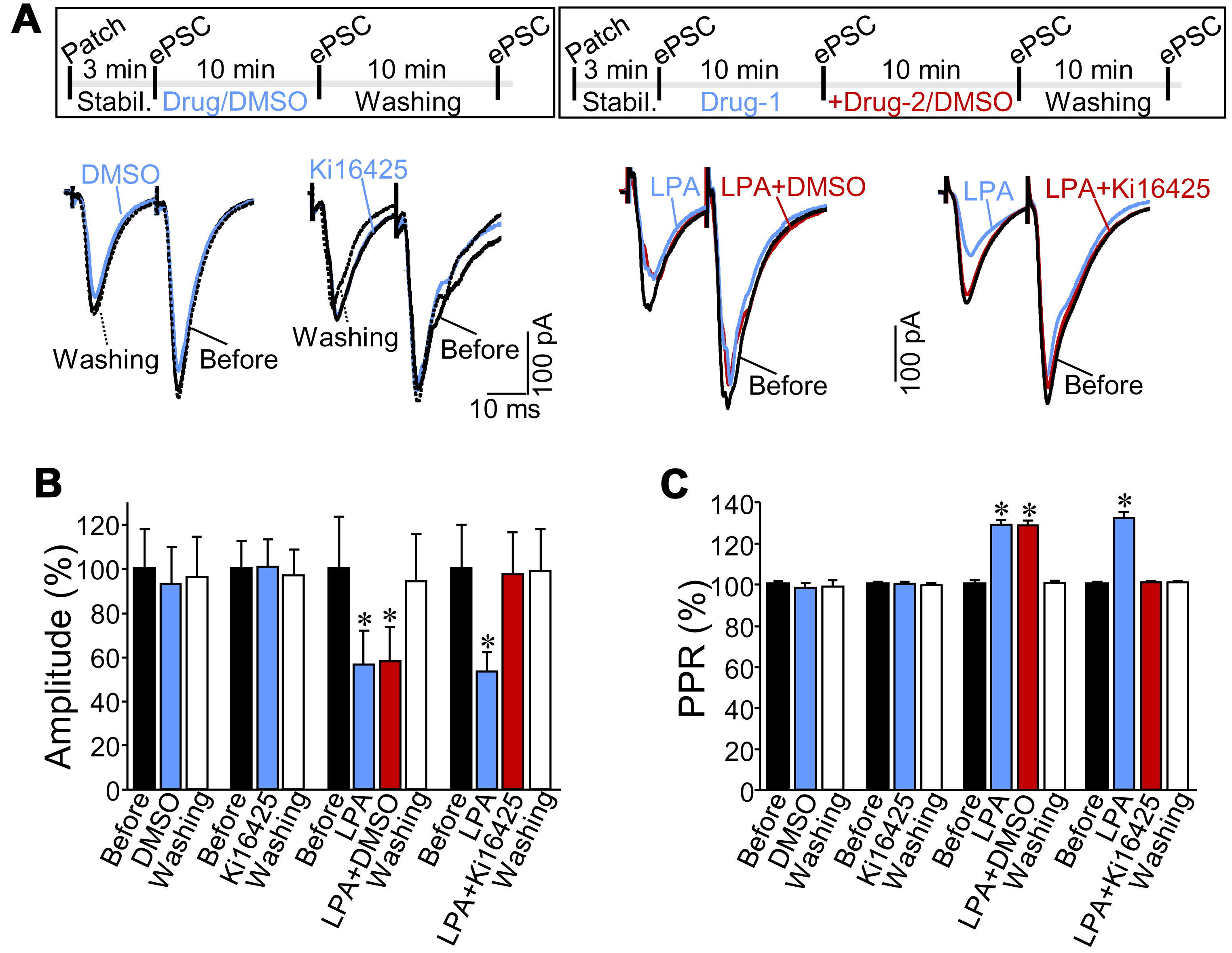

Supplement: S6 Fig — (A) Top, timing of experimental protocols. HMNs were initially allowed to stabilize (Stabil.) with normal aCSF to obtain baseline control recordings. Slices were then superfused for 10 min with aCSF supplemented with 0.2% DMSO, the LPA1/3 inhibitor Ki16425 (0.4 μM in 0.2% DMSO; Drug, left protocol) or LPA (2.5 μM; Drug-1, right protocol) before current responses were acquired again. In the right protocol, slices were additionally incubated for 10 min with LPA plus DMSO or with Ki16425 (0.4 μM; Drug-2). Finally, a last round of acquisition was taken after a 10 min washout with drug-free aCSF. Bottom, representative eEPSCsAMPA from HMNs recorded at the indicated conditions. (B, C) Mean eEPSCsAMPA amplitude (B) and PPR ratio (C) measured at 25 ms interpulse intervals for HMNs recorded under the indicated treatments (n ≥ 5 HMNs per condition). *p < 0.05, one-way RM-ANOVA relative to control (before) condition. Plots data can be found in S1 Data. (TIF) [file pbio.1002153.s007.tif]

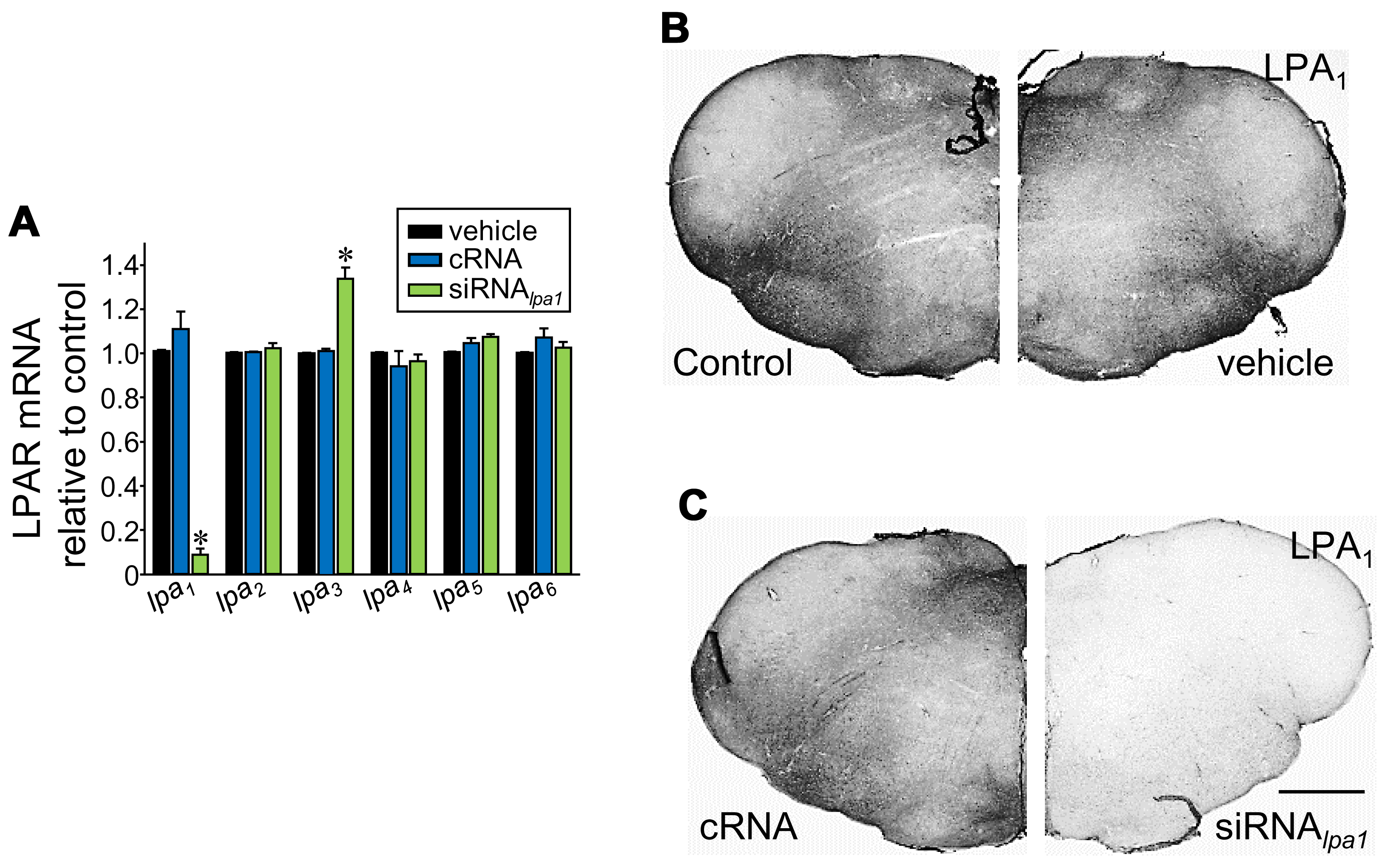

Supplement: S7 Fig — (A) Expression levels of mRNA for indicated LPARs obtained by qRT-PCR of isolated brain stems at P6 after receiving the indicated treatments at P4. GAPDH was used as housekeeping. Values were normalized taking control condition (untreated animals) as 1. *p < 0.05, one-way ANOVA on Ranks relative to control, vehicle, and cRNA conditions for each receptor. (B, C) Immunohistochemistry against LPA1 of brain stem coronal hemisections obtained from P6 pups untreated (Control), or receiving the indicated treatments at P4. Scale bar: 500 μm. Plot data can be found in S1 Data. (TIF) [file pbio.1002153.s008.tif]

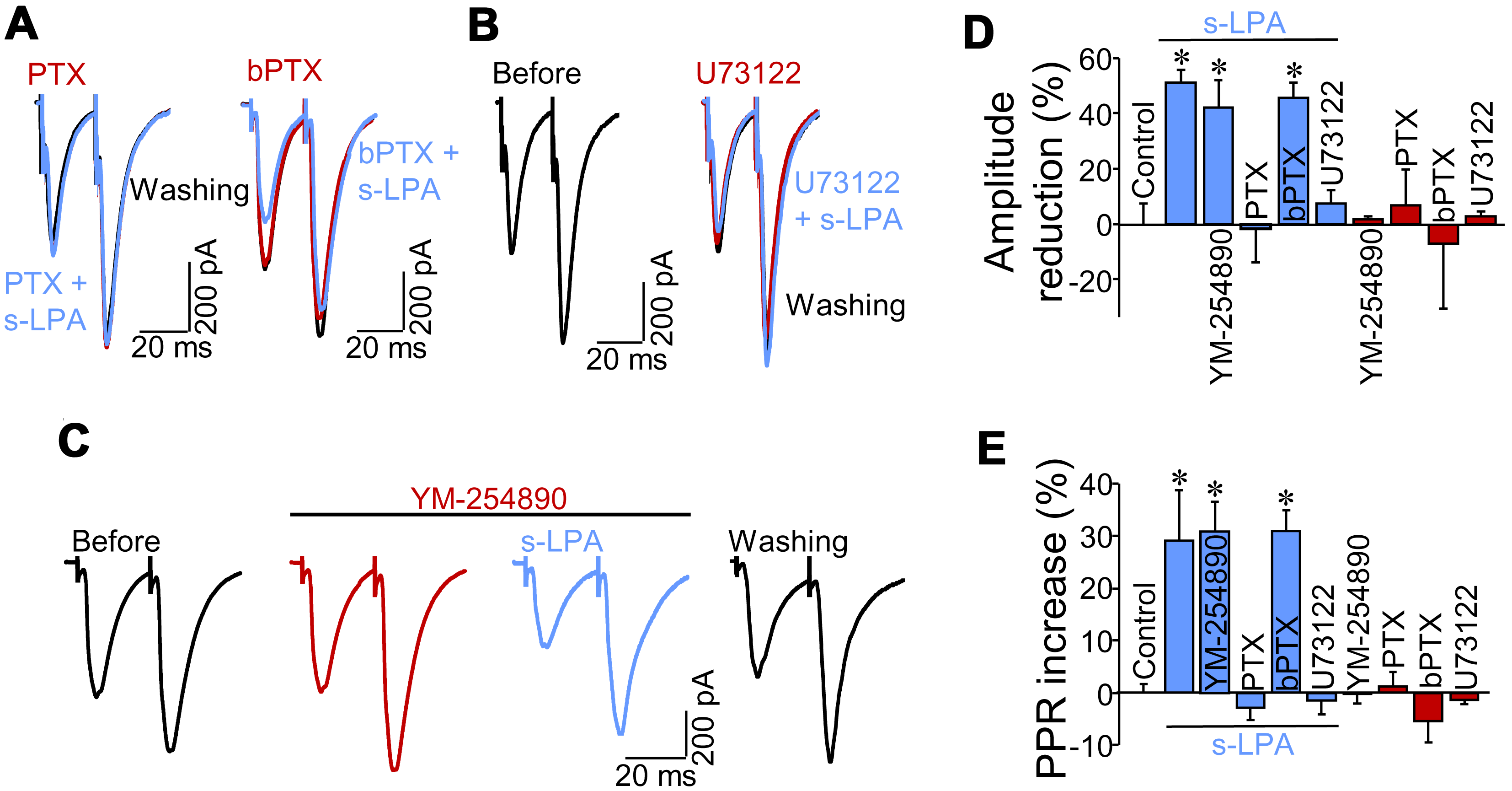

Supplement: S8 Fig — (A–C) Representative recordings showing the effect of s-LPA (40 μM) on eEPSCsAMPA from 4 HMNs in response to paired-pulse stimulation in the presence of the Gαi/o inhibitor PTX (100 ng/ml; A, left), the noncatalytic bPTX (100 ng/ml; A, right), the PLC inhibitor U73122 (1 μM; B), or the Gαq/11 inhibitor YM-254890 (1 μM; C). Stimulus interval was 25 ms. (D, E) Mean eEPSCsAMPA amplitude reduction (D) and PPR ratio increase (E) measured at 25 ms interpulse intervals for HMNs recorded under the indicated treatments (n ≥ 4 HMNs per condition). *p < 0.05, one-way ANOVA relative to control condition. Plots data can be found in S1 Data. (TIF) [file pbio.1002153.s009.tif]

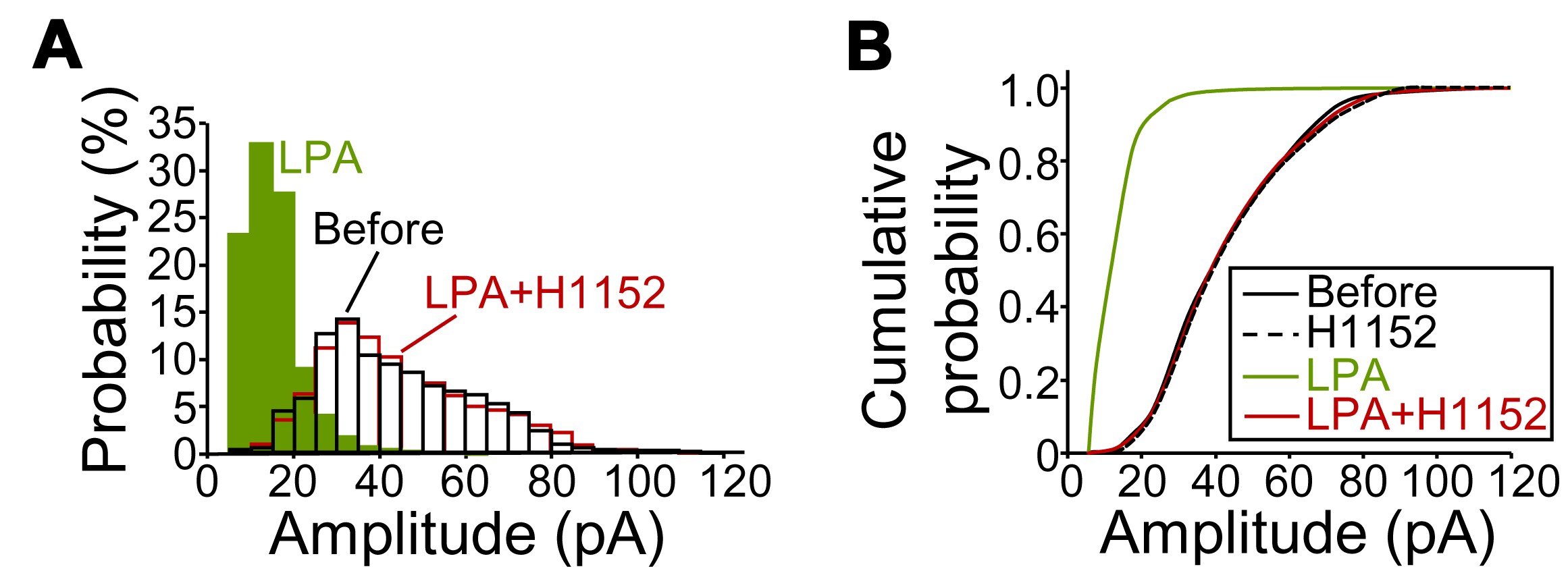

Supplement: S9 Fig — Amplitude distribution histograms (A) and cumulative probability functions (B) of mIPSCsGABAA at the indicated conditions. Each condition is represented by 600 events (5 pA bin width) pooled from 5 HMNs. Note that H1152 reversed the LPA-induced shift to the left of the distribution histograms and the cumulative probability functions of mIPSCsGABAA amplitude (p < 0.05; Kolmogorov-Smirnov test). Plots data can be found in S1 Data. (TIF) [file pbio.1002153.s010.tif]

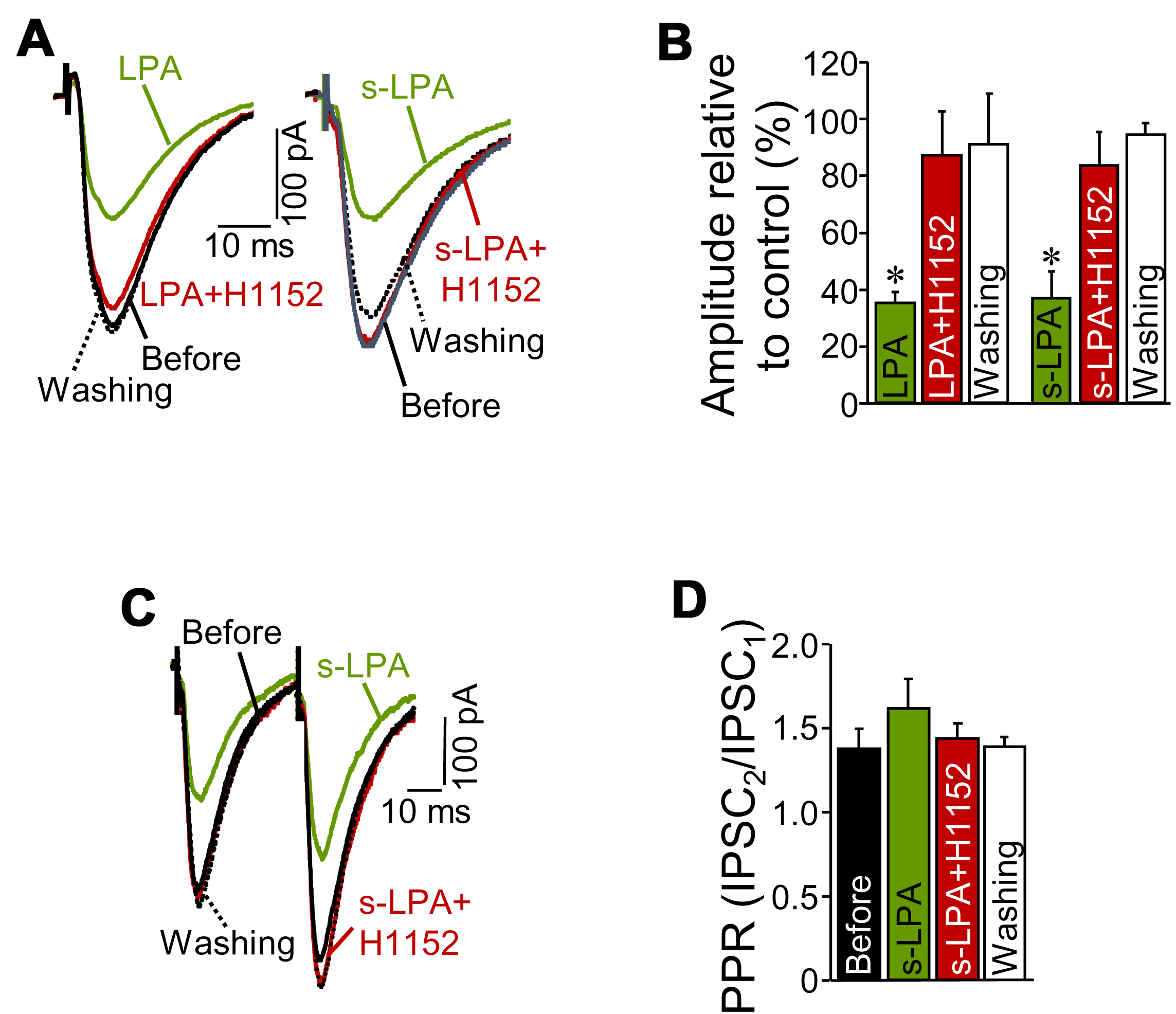

Supplement: S10 Fig — (A, B) Illustrative eIPSCsGABAA of two HMNs (A) and summary data of eIPSCsGABAA amplitude (B) recorded before and after LPA (2.5 μM; left) or s-LPA (40 μM; right) treatment, after the next coaddition of H1152 (20 μM) and subsequent washing (n = 4 HMNs). *p < 0.05, one-way RM-ANOVA relative to the control (before) condition. (C, D) Examples of eIPSCsGABAA recorded in a HMN (C) in response to paired-pulse stimulation of VLRF axons and changes in PPR (D) (n = 4 HMNs). Plots data can be found in S1 Data. (TIF) [file pbio.1002153.s011.tif]

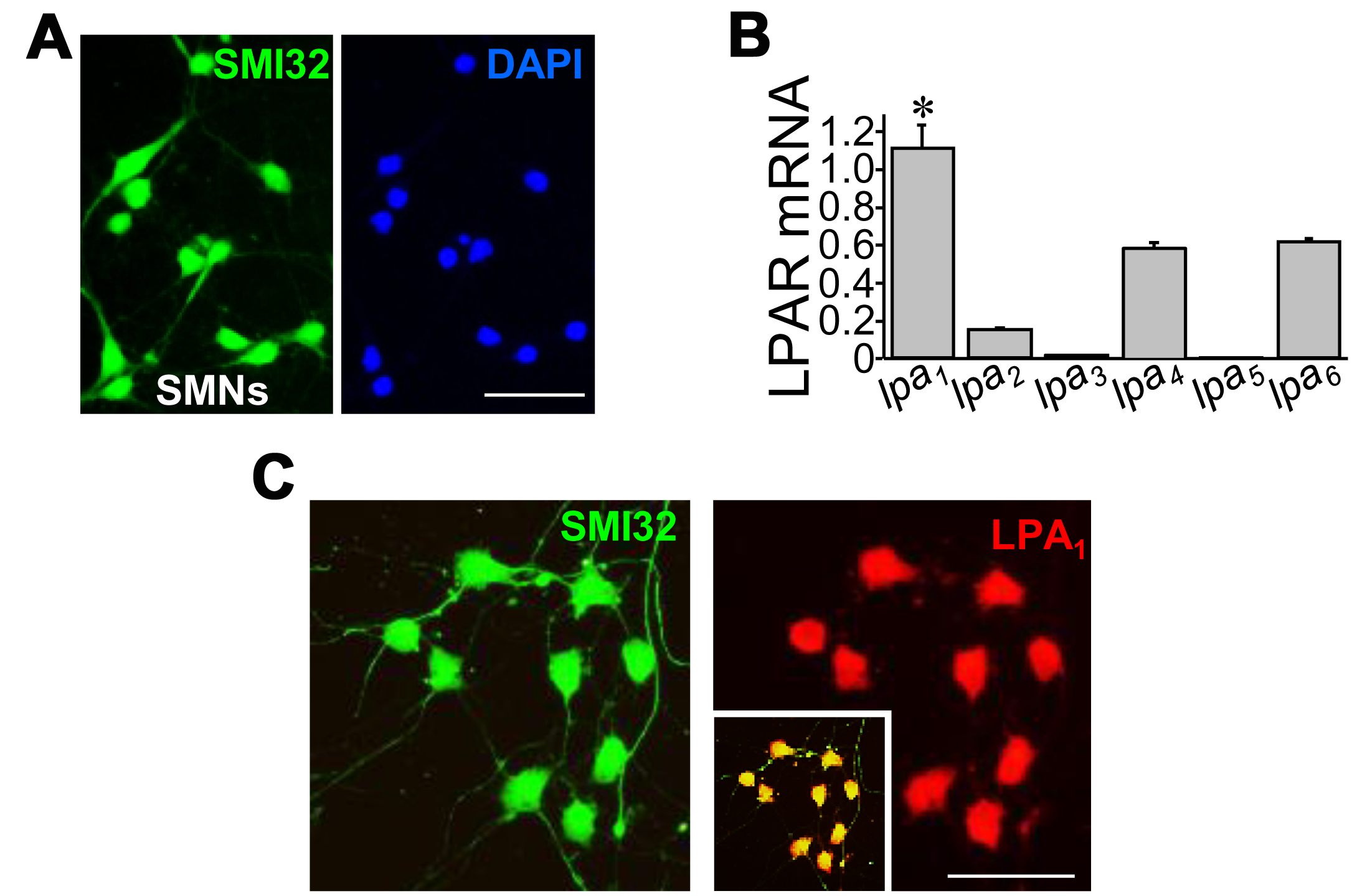

Supplement: S11 Fig — (A) Epifluorescence images of cultured SMNs processed by immunohistochemistry for the motoneuron marker SMI32 (left) and counterstained with the nuclear marker DAPI (right). Note that all cells in the field are SMI32-ir. (B) Expression levels of mRNA for the indicated LPARs obtained by qRT-PCR of cultured SMNs relative to the housekeeping GAPDH. *p < 0.05, one-way ANOVA on Ranks relative to lpa 2–6. (C) Epifluorescence images of cultured SMNs processed by immunohistochemistry for SMI32 (top) and LPA1 (bottom). Scale bars: A, 25 μm; C, 100 μm. Plot data can be found in S1 Data. (TIF) [file pbio.1002153.s012.tif]

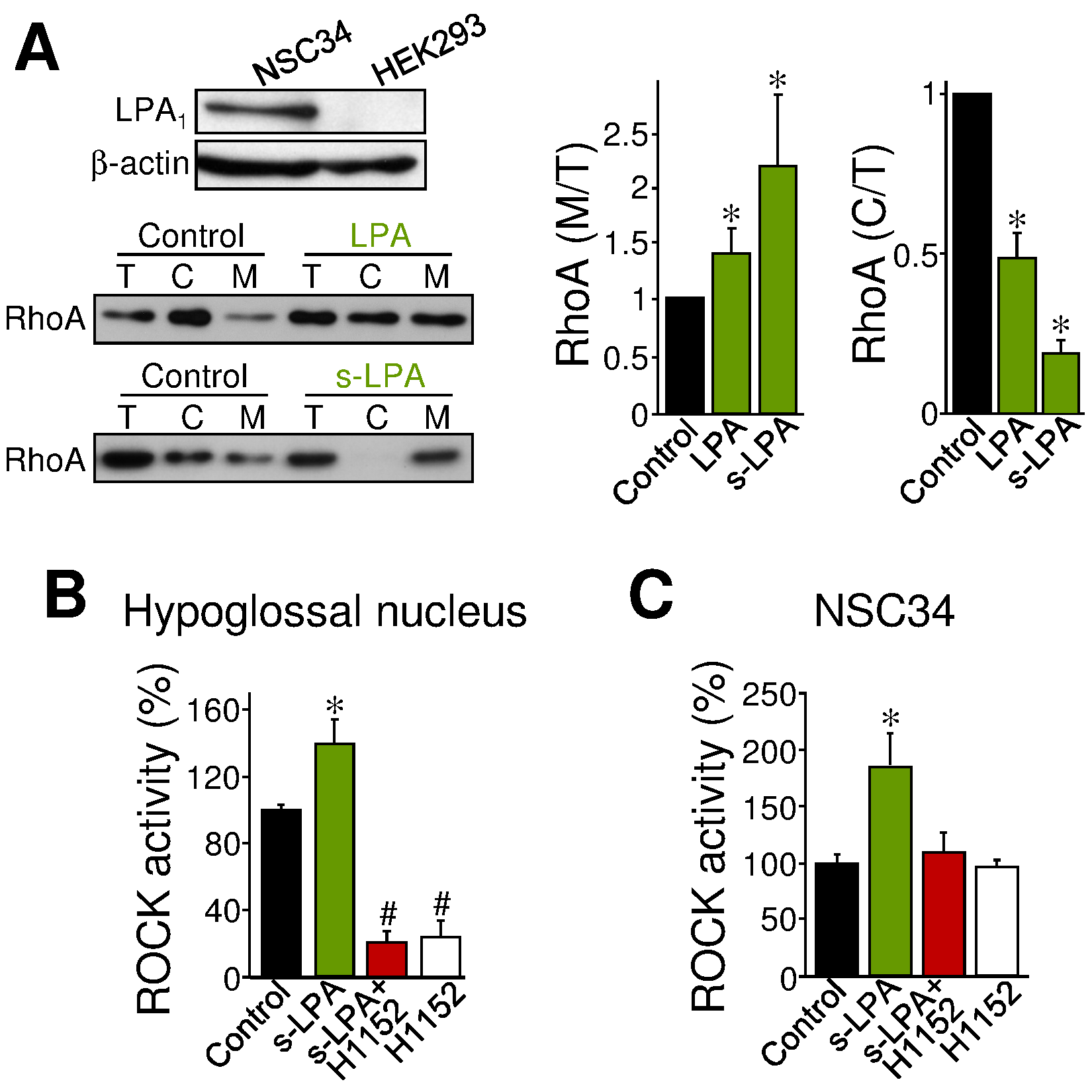

Supplement: S12 Fig — (A) Left, western blots of LPA1 and total (T), cytosolic (C), and membrane-associated (M) RhoA in the motoneuron-like cell line NSC34 after indicated treatments. For LPA1, the cell line HEK293 was taken as a negative control and β-actin expression was used as an internal loading reference. Right, histogram showing the average ratio of densitometric intensity in M or C fractions relative to total RhoA at the indicated conditions. Ratio values were normalized relative to the control group. (B, C) Summary histogram of changes in ROCK activity in homogenates from HN (B) and cultured NSC34 (C) untreated (control) or treated with either s-LPA (40 μM), H1152 (100 μM), or s-LPA plus H1152. *, # p < 0.05, one-way ANOVA on Ranks relative to the control and both control and s-LPA-treated groups, respectively. Plots data can be found in S1 Data. (TIF) [file pbio.1002153.s013.tif]

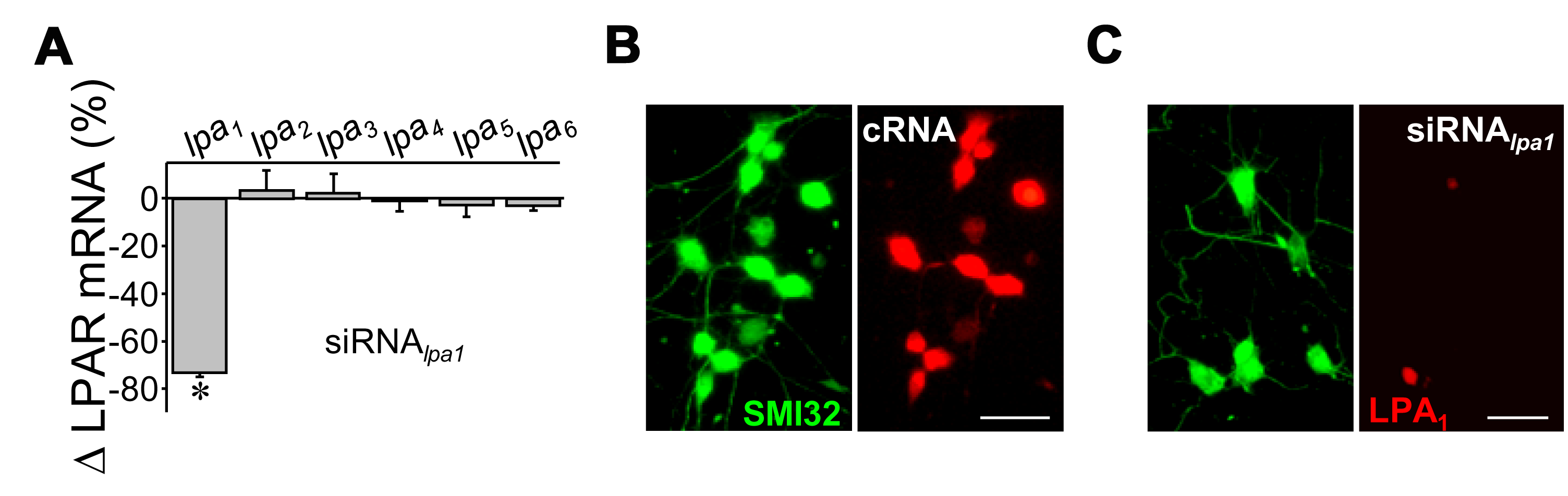

Supplement: S13 Fig — (A) Expression levels of LPAR mRNAs in SMNs after incubation with the small interfering RNA against lpa 1 (siRNAlpa1) relative to cultures treated with a nontargeting siRNA (cRNA). *p < 0.05, one-way ANOVA on Ranks relative to lpa 2–6. (B, C) Epifluorescence images of cultured SMNs receiving the indicated treatments processed by immunohistochemistry for SMI32 and LPA1. Immunohistochemical processing was performed in parallel. Scale bars: 25 μm. Plot data can be found in S1 Data. (TIF) [file pbio.1002153.s014.tif]

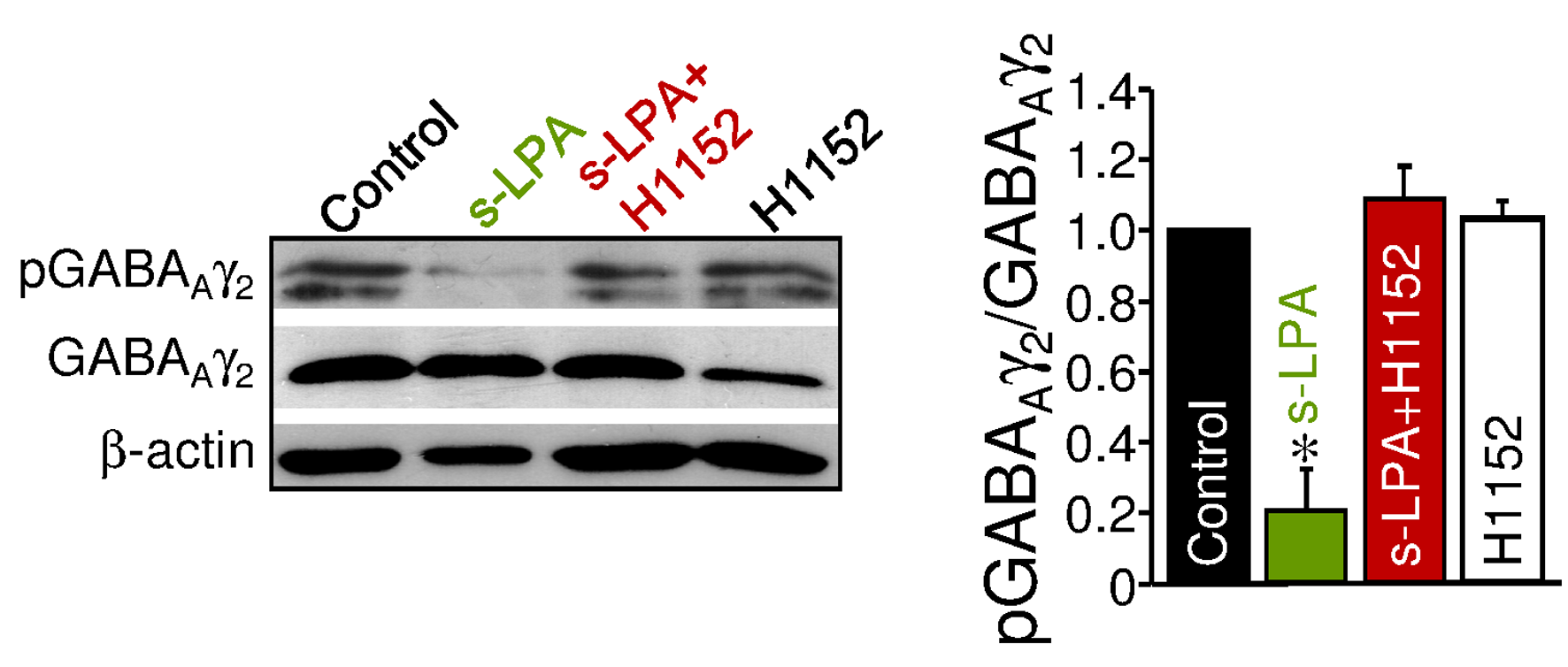

Supplement: S14 Fig — Western blot (top) and averaged ratio (bottom) of phosphorylated and total GABAAγ2 subunit protein levels (denoted as pGABAAγ2 and GABAAγ2, respectively) in the HN of neonatal brain stem slices incubated (10 min) with aCSF alone (control) or supplemented with indicated drugs. β-actin was an internal loading reference. *p < 0.05, one-way ANOVA on Ranks relative to control condition. Plot data can be found in S1 Data. (TIF) [file pbio.1002153.s015.tif]

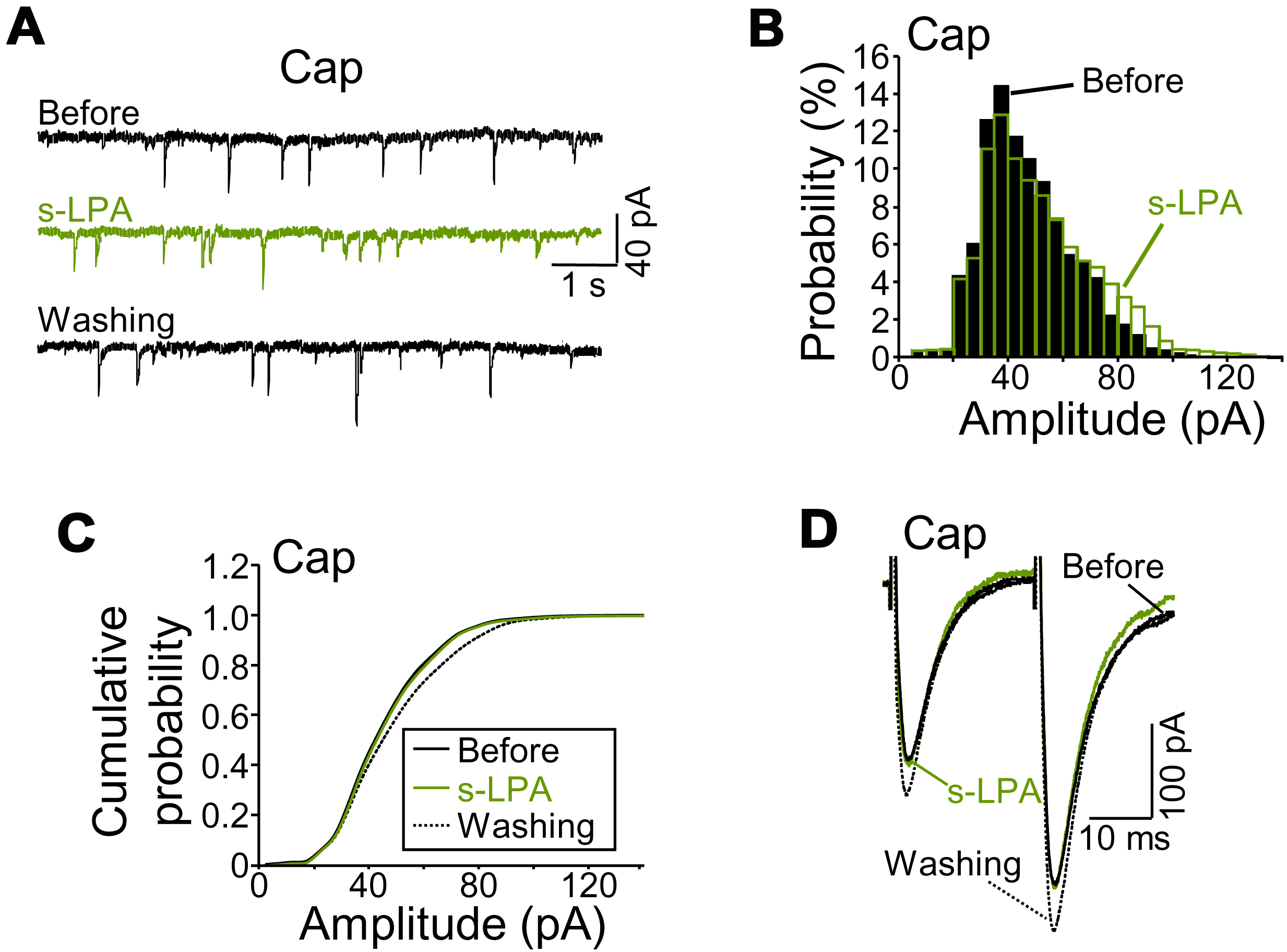

Supplement: S15 Fig — (A–C) Traces of spontaneously occurring mIPSCsGABAA (A), amplitude distribution histograms (B, 5 pA bin size), and cumulative probability functions (C, 5 pA bin size) pooled from 8 HMNs before and after exposure to s-LPA (40 μM). (D) Examples of recorded eIPSCsGABAA in a HMN in response to paired-pulse stimulation of VLRF under the specified treatments. All HMNs were recorded in the presence of CaN autoinhibitory peptide (Cap; 12.5 μM) added to the recording pipette solution. Plots data can be found in S1 Data. (TIF) [file pbio.1002153.s016.tif]
