# Supplementary material for: Membrane-Derived Phospholipids Control Synaptic Neurotransmission and Plasticity
Source: PLoS Biol. 2015 May 21;13(5):e1002153. doi: 10.1371/journal.pbio.1002153 (PMC4440815; doi:10.1371/journal.pbio.1002153)
Supplement: S1 Table — The data used to generate the table can be found in S1 Data. (DOC) [file pbio.1002153.s017.doc]

|  | **Active zone length (μm)** | **Number of a.z. per bouton** | **Bouton area (μm^2^)** | **HMN perimeter (μm)** | **Number of boutons/HMN** |
| --- | --- | --- | --- | --- | --- |
| **Control** | 0.24 ± 0.01 (169) | 1.27 ± 0.05 (133) | 0.35 ± 0.02 (131) | 70.9 ± 2.3 (50) | 2.8 ± 0.3 (86) |
| **DMSO** | 0.23 ± 0.01 (131) | 1.26 ± 0.05 (104) | 0.32 ± 0.02 (98) | 70.1 ± 1.4 (58) | 2.3 ± 0.3 (92) |
| **LPA** | 0.23 ± 0.01 (127) | 1.25 ± 0.05 (102) | 0.31 ± 0.02 (96) | 67.8 ± 1.4 (40) | 2.7 ± 0.3 (65) |
| **LPA+**  **ML-7** | 0.26 ± 0.01 (146) | 1.18 ± 0.04 (124) | 0.37 ± 0.02 (118) | 70.5 ± 1.5 (45) | 2.3 ± 0.3 (67) |

Number of sampled motoneurons, synaptic terminals or active zones (a.z.) is indicated in parentheses.
