# Supplementary material for: Membrane-Derived Phospholipids Control Synaptic Neurotransmission and Plasticity
Source: PLoS Biol. 2015 May 21;13(5):e1002153. doi: 10.1371/journal.pbio.1002153 (PMC4440815; doi:10.1371/journal.pbio.1002153)
Supplement: S2 Table — (DOC) [file pbio.1002153.s018.doc]

| Oligonucleotide primer sequences (5' 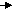 3') | | | | | | |
| --- | --- | --- | --- | --- | --- | --- |
| **Gene** | **Forward** | **Reverse** | **Product**  **size (bp)** | | | **Annealing temperature (ºC)** |
|  |  |  | |  |  | |
| *For mice* | | | |  |  | |
|  |  |  | |  |  | |
| *lpa_1_* | TGACTGTTAGCACGTGGCTCCT | GGAAAACCGTGATGTGCCTCT | | 108 | | 60 |
| *lpa_2_* | TACCTGCACACTTCTGGCACTG | GGAAGACAAGCAGGCTGGATAG | | 114 | | 60 |
| *lpa_3_* | ACATCTCGGCCTGCTCTTCTCT | AGATGCGTACGTATACCGCCAC | | 114 | | 60 |
| *lpa_4_* | TTATATGCCTTGGTACGCTCCC | CAATTCAGAGTTGCAAGGCACA | | 101 | | 60 |
| *lpa_5_* | TGCTTCGTGCCCTATAACTCCA | ACCATTATCATCAGCACCCCG | | 113 | | 60 |
| *lpa_6_* | TTGCATTGCTGTGTGGTTCAC | GCCGCTGGAAAGTTCTCAAA | | 117 | | 60 |
| *gapdh* | AGAACATCATCCCTGCATCCA | AGATCCACGACGGACACATTG | | 124 | | 60 |
|  |  |  | |  | |  |
| *For rats* |  |  | |  | |  |
|  |  |  | |  | |  |
| *lpa_1_* | GGAACACTGTGAGCAAGCTGGT | GCGGTTGACGTAAATTGCCA | | 101 | | 60 |
| *lpa_2_* | TGGTATTGCTGACCAACCTGC | GGCCAGTATGGAACATGAGGAA | | 147 | | 60 |
| *lpa_3_* | TCTGCAACATCTCAGCCTGCT | CACCATGATGAAGAAGGCCAG | | 101 | | 60 |
| *lpa_4_* | ACAACTTTAACCGCCACTGGC | CGATCCACACTAATGCAGGTGA | | 115 | | 60 |
| *lpa_5_* | TCCTACTATGCACGGCACCACT | ATAGCGGTCCACGTTGATGAGC | | 123 | | 60 |
| *lpa_6_* | TGCTGTGTGGTTCACCGTGAT | TCCATGTGGCTGCTGGAAAGT | | 119 | | 60 |
| *gapdh* | AGAACATCATCCCTGCATCCA | AGATCCACGACGGACACATTG | | 124 | | 60 |
