# Supplementary material for: Membrane-Derived Phospholipids Control Synaptic Neurotransmission and Plasticity
Source: PLoS Biol. 2015 May 21;13(5):e1002153. doi: 10.1371/journal.pbio.1002153 (PMC4440815; doi:10.1371/journal.pbio.1002153)
Supplement: S1 Text — (DOC) [file pbio.1002153.s019.doc]

**SUPPORTING INFORMATION**

**Results**

***Settings for isolation and recording of the AMPAergic and GABA_A_ergic components of ePSCs***

Electrical stimulation of the ventrolateral reticular formation (VLRF) evokes post-synaptic currents (ePSCs) in HMNs (Fig. 2A) using KGluconate-filled electrodes [1,2]. *In vivo*, most HMNs exhibit a repetitive rhythmic firing, consisting of inspiratory-related bursting discharges mainly driven by AMPAR-mediated excitatory brainstem afferences [3,4]. In the present study, we pharmacologically isolated the AMPAergic component of excitatory PSCs (eEPSCs_AMPA_) evoked by electrical stimulation of the VLRF in HMNs held at -65 mV (Fig. 2B, left traces). ePSCs also possess a subtle inhibitory component (eIPSCs) [2]. To maximize eIPSCs generated by the opening of chloride-selective channels (glycine and GABA_A_ receptors), HMNs were held at -70 mV and recorded with a high-chloride pipette solution. Evoked GABA_A_ergic IPSCs (eIPSCs_GABAA_) were pharmacologically isolated to assure that the remaining eIPSCs were GABA_A_R-mediated [2] (Fig. 2B, middle traces).

***Effect of vehicle on excitatory and inhibitory inputs***

Continuous perfusion with vehicle (artificial cerebrospinal fluid [aCSF]) for 10 min resulted in a significant reduction (*p* < 0.05, one-way RM-ANOVA) of -4.3 ± 0.7% for eEPSCs_AMPA_ (n = 5 HMNs) and -6.3 ± 3.6% for eIPSCs_GABAA_ (n = 6 HMNs). Attenuation did not increase after 10 min of additional washing. Mean values of vehicle-induced attenuation of ePSCs were subtracted from changes in ePSCs amplitude induced by the different concentrations of LPA tested (Fig. 2C).

***LPA does not act post-synaptically on AMPAergic signaling***

Miniature quantal events represent the post-synaptic responses to transmitter release from single SVs, and the average amplitude is taken as a measure of the sensitivity and/or number of post-synaptic receptors [5,6]. First, we examined the effect of LPA on pharmacologically isolated miniature EPSCs_AMPA_ (mEPSCs_AMPA_); we found no significant effect on amplitude (S1A Fig.). The mean amplitude of mEPSCs_AMPA_ recorded after 10 min exposure to this phospholipid was 21.9 ± 0.8 pA, which was comparable to the average mEPSCs_AMPA_ amplitude measured before drug administration (23.3 ± 1.2 pA, *p* = 0.20, one-way RM-ANOVA). This was also supported by the drug’s lack of effect on the cumulative probability function of mEPSCs_AMPA_ amplitude (S1B Fig.; *p* = 0.21; Kolmogorov-Smirnov test). Likewise, no LPA effect on the average mEPSCs_AMPA_ frequency was observed (before: 8.0 ± 1.6 Hz; LPA: 11.3 ± 3.0 Hz, *p* = 0.367, one-way RM-ANOVA). Additionally, HMNs were tested for their sensitivity to exogenously applied glutamate pulses before and after LPA application. Exogenous glutamate pulses consistently evoked large inward AMPAergic currents (4.18 ± 0.33 nA) in all 7 HMNs tested (S1C Fig.). Bath perfusion (10 min) of LPA had no significant effect on the mean peak amplitude (4.12 ± 0.27 nA, *p* = 0.50, one-way RM-ANOVA) of the glutamate-evoked currents (S1C Fig.). These post-synaptic responses were effectively blocked (>95%) with the AMPAR antagonist NBQX [2]. Therefore, the inhibitory effect of LPA signaling on AMPAR-mediated transmission is likely not attributable to changes in the post-synaptic sensitivity to glutamate.

***LPA potentiates the facilitation index of eEPSCs_AMPA_ under repeated VLRF stimulation***

Repeated VLRF stimulation with a series of 20 shocks at 40 Hz also resulted in a powerful facilitation of eEPSCs_AMPA_, with a progressive rise in the eEPSCs_AMPA_ amplitude throughout the train (S3 Fig.). The standard facilitation index was calculated by measuring the amplitude of the individual eEPSCs_AMPA_ throughout the train, compared to the first eEPSC_AMPA_. In control recordings, the average values of the facilitation index for the 10^th^ and 20^th^ eEPSCs_AMPA_ were 2.07 ± 0.23 and 2.25 ± 0.35, respectively. LPA reversibly potentiated the facilitation index from the 6^th^ to the 20^th^ eEPSCs_AMPA_ in all HMNs tested (S3 Fig.). Thus, after LPA the facilitation index for the 10^th^ eEPSCs_AMPA_ was 3.28 ± 0.32 and 4.08 ± 0.46 for the 20^th^.

***Evidence for LPA_1_ as a key receptor mediating LPA-induced depression of AMPAR-mediated synaptic neurotransmission***

*q*RT-PCR and immunohistochemical studies point to LPA_1_ as a pivotal LPA receptor affecting glutamatergic synapses. In line with this hypothesis, addition to the bath of the LPA_3_ specific agonist OMPT (1 μM) for 10 min at an effective dose to activate LPA_3_ [7] or the LPA_1/3_ antagonist VPC 32183 (1 μM) did not alter AMPAR-mediated eEPSCs *in vitro* (S5 Fig.). However, VCP 32183 fully reversed s-/LPA-induced excitatory depression (S5 Fig.). These results were ratified by another selective inhibitor of LPA_1/3_, Ki16425 [8]. Incubation for 10 min with vehicle (0.2 % DMSO) or with Ki16425 (0.4 μM) did not alter eEPSCs_AMPA_ amplitude nor PPR (S6 Fig.). However, Ki16425 (0.4 μM), but not vehicle (amplitude: -42.1 ± 15.6%; PPR: 28.8 ± 2.5%), completely reversed changes induced by LPA on amplitude (-2.6 ± 19.1%) and PPR (0.8 ± 0.6%) (S6 Fig.). In our experimental conditions, baseline LPA_1/3_-mediated signaling seems to not regulate excitatory synaptic strength, and LPA_3_ agonism does not mimic LPA-induced eEPSCs_AMPA_ STD. Altogether, these data point to LPA_1_ as a likely key receptor mediating LPA-induced depression of AMPAR-mediated synaptic transmission.

***Effectiveness of siRNA_lpa1_ in knockdown LPA_1_ in the brainstem of neonatal rats***

Sequence-specific gene silencing using small interfering RNA (siRNA) is a promising method, with potential clinical utility, for inhibiting the expression of targeted genes and inducing knockdown of associated proteins both *in vitro* and *in vivo* [9,10]. Here, we have used a modified siRNA that after a single intracerebroventricular injection in the adult rat induces a rapid and targeted knockdown of the selected genes in the CNS, with absence of off-target effects, toxicity, and recruitment of immune responses [9]. A siRNA against *lpa_1_* (siRNA*_lpa1_*, 2 μg/2 μl) was injected into the fourth ventricle of rat pups at P4. As controls for this group of experiments, the vehicle (RNase-free phosphate buffered saline [PBS], 2 μl) or a control non-interfering siRNA (cRNA, 2 μg/2 μl) were administered (Fig. 4A). Injected siRNA*_lpa1_* was selected, from a pool of four supplied by the manufacturer as the most effective to silence *lpa_1_* in primary cultures of spinal cord motoneurons (SMNs) isolated from mouse embryos at E12.5. Histological and electrophysiological studies were carried out at P6, when maximal down-regulation of *lpa_1_* in the brainstem was confirmed by *q*RT-PCR in siRNA*_lpa1_*- relative to cRNA-treated animals (P6: -91.2 ± 3.0%; P7: -58.1 ± 10.5%; P8: -43.9 ± 2.3%; n = 3 animals per age and treatment). *q*RT-PCR analysis of the *lpa_2-6_* transcripts revealed that only *lpa_3_* expression levels were slightly up-regulated (+33.6 ± 5.3%) by siRNA*_lpa1_*-treatment (S7A Fig.). Consistent with *q*RT-PCR studies, mean optical density analysis revealed a strong reduction (~74%) in LPA_1_-ir in brainstem sections from siRNA*_lpa1_*-treated pups (26.1 ± 2.5%; *p* < 0.001, one-way ANOVA) relative to untreated (100.0 ± 3.4%) or vehicle- (90.8 ± 5.1%) or cRNA-injected (99.9 ± 4.6%) pups (S7B and S7C Fig.). *q*RT-PCR and immunohistochemistry indicated that LPA_1_ expression was efficiently reduced at two days after a single injection of siRNA*_lpa1_*, at least in the brainstem.

***LPA-induced excitatory STD seems to not be mediated by G_α12/13_ protein***

It would be expected that LPA effects on eEPSCs_AMPA_ do not involve the G_α12/13_, given that an inhibitor of the main G_α12/13_-effector ROCK [11] attenuated (by >50%) eEPSCs_AMPA_ amplitude *per se* by a pre-synaptic mechanism of action [2]. As anticipated, H1152 (10 min, 20 μM), a specific inhibitor of ROCK, did not alter STD evoked by LPA (LPA: -39.5 ± 8.6%; LPA+H1152: -37.7 ± 3.7%; n = 7 HMNs).

***Identification, characterization and quantitative effects of LPA on the SV content of S-type boutons***

Ultrastructural organization of excitatory (S-type) synaptic boutons on HMNs in control and treated neonatal brainstem slices were analyzed. S-type boutons were classified by the type of SVs (spherical), assisted by asymmetric synaptic densities when these were evident [1,12]. To quantify the spatial distribution of SVs relative to the a.z. within S-type boutons, three regions were delimited by lines parallel to the a.z. profile and the number of vesicles included in the intervals of 0-100 nm, 100-200 nm, and 200-300 nm from the a.z. were counted (Fig. 5G). To evaluate potential effects on vesicle trafficking and/or recycling, we also counted the number of SVs included in the total pool for each analyzed bouton in the same section where intervals were analyzed (Fig. 5H).

The two regions closest to the a.z. showed a decline in the number of SVs after LPA treatment (0-100 nm: -25.9 ± 3.9%; 100-200 nm: -20.1 ± 5.5%), which was absent in slices co-incubated with LPA plus ML-7 (0-100 nm: -4.8 ± 4.6%; 100-200 nm: -4.1 ± 5.5%), compared to slices incubated with aCSF alone or supplemented with 0.2% DMSO, the vehicle for ML-7 (Fig. 5K and 5L). Since none of the treatments altered the total number of SVs per a.z., effects on SVs trafficking and/or recycling could be discarded (Fig. 5L). No effects were observed on the average a.z. length, mean number of a.z. per bouton, average number of excitatory boutons per motoneuron, or mean cross-sectional bouton area (S1 Table).

***LPA_1_ expression assessment in SMNs***

Post-synaptic action and the molecular signaling underlying LPA-induced modulation of GABA_A_ergic system were assessed in SMNs isolated from mouse embryos (~12.5 days). After 4-5 days *in vitro*, virtually all cells in cultures were immunoreactive for the motoneuron marker SMI32 (S11A Fig.). *q*RT-PCR analysis showed that SMN cultures predominantly expressed *lpa_1_* relative to *lpa_2-6_* transcripts (S11B Fig.). All SMI32-positive SMNs were also LPA_1_-ir (S11C Fig.).

***Evidence that LPA signaling activates the RhoA/ROCK pathway in motoneurons***

Supplementary experiments were carried out to look for evidence that LPA stimulates RhoA/ROCK pathway in motoneurons. We observed that s-/LPA (for 10-15 min) increased the relative amount (LPA: +40.1 ± 21.9%; s-LPA: +116.5 ± 62.2%) of the small GTP-binding protein RhoA, the major ROCK activator, in the membrane (M) fraction of the motoneuron-like cell line NSC34, which also expresses (at least) LPA_1_ (S12A Fig.). s-/LPA induced a parallel reduction of RhoA in the cytosolic (C) fraction (LPA: -51.1 ± 7.8%; s-LPA: -80.9 ± 4.3%; S12A Fig.). These observations indicate that both phospholipids induce RhoA activation in this cell line. ROCK activity, in microdissected HNs from rat pups (P7), increased after incubation with s-LPA (+39.7 ± 14.7%). H1152 application during the activity assay reduced ROCK activity in both untreated and s-LPA-treated HNs (-79.5 ± 7.1%; -76.2 ± 9.8%, respectively) (S12B Fig.). Therefore, under our experimental conditions there is a baseline ROCK activity in HN from rat pups. Interestingly, s-LPA enhanced ROCK activity (+84.9 ± 38.8%) in NSC34 cells but it remained as in control when s-LPA-treated NSC34 cells were incubated with H1152 during the activity assay (+9.4 ± 17.6%) (S12C Fig.). This finding indicates that the ROCK activity was fully due to s-LPA treatment in this cell line. In addition, H1152 did not reduce ROCK activity (-4.4 ± 6.5%) in samples from untreated NSC34 (S12C Fig.). Thus, there is no evidence of endogenous ROCK activity in this cell line in our experimental conditions. Taken together, these data indicate that LPA signaling activates the RhoA/ROCK pathway, at least, in motoneurons.

***Effectiveness of siRNA_lpa1_ in knockdown LPA_1_ in SMNs***

Pre-incubation with siRNA*_lpa1_* robustly reduced (-74.0 ± 9.0%) the amount of *lpa_1_* mRNA in SMNs, but did not alter the levels of *lpa_2-6_* transcripts, relative to cRNA (S13A Fig.). LPA_1_ knockdown was also supported since LPA_1_-ir almost fully disappeared after siRNA*_lpa1_* treatment (S13B and S13C Fig.).

***s-LPA induces GABA_A_γ_2_ dephosphorylation in the HN by a ROCK-dependent mechanism***

Contrary to expectations of a direct interaction between ROCK and GABA_A_γ_2_, s-LPA induced a robust reduction (-79.3 ± 11.5%) of the pGABA_A_γ_2_/GABA_A_γ_2_ ratio in the HN, which was not explained by a change in GABA_A_γ_2_ expression levels (-9.4 ± 6.4%, *p* = 0.438, one-way ANOVA on Ranks) relative to β-actin (S14 Fig.). Co-addition to the bath of H1152 fully prevented (+9.0 ± 9.1%) any s-LPA-induced decrease in pGABA_A_γ_2_. Furthermore, H1152 did not modify (+3.0 ± 5.4%) the pGABA_A_γ_2_/GABA_A_γ_2_ ratio by itself (S14 Fig.). Therefore, ROCK had no effect on phosphorylation of Ser^327^GABA_A_γ_2_, at least under basal conditions, on the timescale tested.

***s-LPA-induced alterations in mIPSCs_GABAA_ and eIPSCs_GABAA_ in HMNs were CaN-dependent***

Addition to the recording pipette solution of the CaN auto-inhibitory peptide (Cap; 12.5 μM) increased amplitude (Cap: 42.6 ± 1.4 pA, n = 8 HMNs; untreated: 34.5 ± 1.2 pA, n = 10 HMNs, *p* = 0.003, one-way ANOVA on Ranks), but not frequency (Cap: 4.0 ± 0.2 Hz; untreated: 3.3 ± 1.8 Hz; *p* = 0.965), of mIPSCs_GABAA_ recorded in HMNs relative to untreated ones. Thus, baseline activity of CaN, acting at a post-synaptic level, likely restrains GABA_A_ergic inputs on HMNs in our experimental conditions. Cap prevented s-LPA-induced reduction of mIPSCs_GABAA_ amplitude (41.3 ± 2.2 pA, *p* = 0.125; n = 4 HMNs), as verified by the distribution histograms and the cumulative probability functions (S15B and S15C Fig.; Kolmogorov-Smirnov test). s-LPA did not alter eIPSCs_GABAA_ amplitude (before: 179.9 ± 33.5 pA; s-LPA: 252.6 ± 45.9 pA; n = 5 HMNs) and PPR (before: 1.8 ± 0.1; s-LPA: 1.6 ± 0.1) under Cap treatment (S15D Fig.). These results point to CaN as a pivotal enzyme in the intracellular molecular cascade mediating LPA_1_-induced modulation of inhibitory synaptic strength.

**Extended Materials and Methods**

Wistar rats of either sex and CD1 pregnant mice were obtained from an authorized supplier (Animal Supply Services, University of Cádiz, Spain), and were cared for and handled in accordance with the guidelines of the European Union Council (86/609/UE) and Spanish regulations (BOE 67/8509-12; BOE 1201/2005) on the use of laboratory animals. Animals were individually housed -except neonatal animals, which were housed with their mother- in cages with water and food pellets available ad libitum, under temperature-controlled conditions at 21 ± 1ºC, with a 12-h light/dark cycle. Efforts were made to minimize the number of animals used and their suffering. All surgical procedures were carried out under aseptic conditions. Experimental procedures were approved by the local Animal Care and Ethics Committee.

***Electrophysiological recordings***

**In vitro *whole-cell patch-clamp recordings of motoneurons***

Whole-cell patch-clamp recordings were obtained from cultured SMNs or HMNs from coronal brainstem slices as previously described [1,2,13]. Neonatal rats (P6-P9) were anesthetized by hypothermia (10-15 min at 4ºC), decapitated, and their brainstems rapidly extracted [1]. Dissection was in sucrose aCSF at 4°C (in mM: 26 NaHCO_3_, 10 glucose, 3 KCl, 1.25 NaH_2_PO_4_, 2 MgCl_2_, and 218 sucrose) bubbled with 95% O_2_ and 5% CO_2_. Transverse slices (300-400 μm-thick), obtained using a vibroslicer (NVSL; WPI), were transferred to normal oxygenated aCSF (in mM: 26 NaHCO_3_, 10 glucose, 3 KCl, 1.25 NaH_2_PO_4_, 2 MgCl_2_, 130 NaCl, and 2 CaCl_2_) and allowed to stabilize at ~37°C for 30 min. Slices were then transferred to a recording chamber for whole-cell patch-clamp recordings.

SMN cultures were prepared from embryonic day 12.5 (E12.5) mouse spinal cords, essentially as previously described [14]. Isolated cells were pooled in a tube containing culture medium and plated. Cultured SMNs were clearly identified by immunofluorescence using the SMI32 antibody or by morphological criteria (Fig. 5I) [14]. Isolated SMNs were plated on 24-mm Corning glass coverslips (Corning, NY) for electrophysiological experiments (19,000 neurons per well). Culture medium was Neurobasal (Gibco, Invitrogen, Paisley, UK) supplemented with B27 (Gibco; Invitrogen), horse serum (2% v/v), L-glutamine (0.5 mM), and 2-mercaptoethanol (25 μM; Sigma-Aldrich) and a cocktail of recombinant neurotrophic factors: 1 ng/mL brain derived neurotrophic factor, 10 ng/mL glial cell-line derived neurotrophic factor, 10 ng/mL ciliary neurotrophic factor, and 10 ng/mL hepatocyte growth factor (PreProtech, London, UK). Experimental procedures were performed 4-6 days after plating.

Recordings were performed at 31ºC on slices or SMN cultures superfused (rate ~3-4 ml/min) with aCSF solution equilibrated with 95% O_2_ and 5% CO_2_. HMNs or SMNs were identified based on their characteristic size and shape and patched under visual guidance using infrared-differential interference contrast optics. Patch electrodes (1.5-3 MΩ resistance) contained either a KGluconate-based internal solution (composition in mM: 17.5 KCl; 122.5 KGluconate, 9 NaCl; 1 MgCl_2_; 10 HEPES; 0.2 EGTA; 3 Mg-ATP, 0.3 GTP-Tris with pH buffered to 7.2) to record EPSCs or a CsCl-based internal solution (composition in mM: 120 CsCl; 4 NaCl; 4 MgCl_2_; 1 Cl_2_Ca; 10 HEPES; 0.2 EGTA; 3 Mg-ATP, 0.3 GTP-Tris) to record IPSCs_GABAA_. Whole-cell AMPAergic responses were recorded at a holding potential of -65 mV. GABA_A_ post-synaptic currents were recorded in cells voltage-clamped at -70 mV. Voltage-clamp recordings were obtained and low-pass bessel-filtered at 10 kHz with a MultiClamp 700B amplifier. Data were digitized at 20 kHz with a Digidata 1332A analog-to-digital converter and acquired using pCLAMP 9.2 software (Axon Instruments, Foster City, CA). Only recordings with access resistance between 5 and 20 MΩ were considered acceptable for analysis. The access resistance was checked throughout the experiments, and recording was abandoned if it changed >15%. Series resistance was routinely compensated 65% to 75%. Leak currents and liquid junction potentials were not corrected. Data were stored on a computer disk and analyzed off-line by using MiniAnalysis (Synaptosoft, Inc, Decatur, GA) and Clampfit 9.2 (Axon Instruments, Foster City, CA) software.

For each cell, peak amplitudes and inter-event intervals of single mEPSCs_AMPA_, mIPSCs_GABAA_ and sEPSCs_AMPA_ were measured across the different recording periods and used to calculate mean values and generate cumulative probability plots. For each cell and treatment condition, we obtained segments of continuous recording (5-8 min) of spontaneous activity. During analysis, the threshold level for detection of events was set at 3 times the root-mean-squares noise (~2-3 pA, detection threshold ~6-9 pA). Spurious events and noise artifacts were manually rejected after visual inspection of the automatically screened events.

To test sensitivities of motoneurons to glutamate or GABA we placed, at 75 to 100 µm distance from the recorded cells, a second micropipette containing 1 mM glutamate or GABA (Tocris) coupled to a Picospritzer II (General Valve Corporation, Fairfield, NJ) pressure ejection system. The cells were voltage clamped at -65 mV or -70 mV and tested for their response to 100 ms pulses of glutamate or GABA (20 or 10 psi, respectively). To avoid contamination from other possible synaptic currents evoked by the pulse, aCSF was replaced with a modified extracellular solution containing TTX and nominally zero Ca^2+^. The AMPAergic or GABA_A_ergic component of the evoked currents was pharmacologically isolated as indicated in the legend of Fig. 1M. For each cell we obtained one or two series of test pulses (5 to 10 at 0.05 Hz) under each experimental condition. Traces were averaged and peak amplitude of response was measured for each treatment condition in each cell.

eEPSCs_AMPA_ and eIPSCs_GABAA_ were elicited by means of a concentric bipolar tungsten electrode placed in the VLRF about 0.5 to 1 mm from the border of the HN. Stimulation parameters (<400 μA, 50-μs, 0.05 Hz) were chosen to generate a sizable and reproducible post-synaptic response. As otherwise stated, minimal stimulus intensity to evoke a post-synaptic response of maximal amplitude was adjusted and then it was maintained constant throughout the recording period. A minimum of 10 responses was recorded under each pharmacological condition and averaged for analysis of eEPSCs_AMPA_ or eIPSCs_GABAA_ amplitudes. The effect of LPA on evoked glutamate release at VLRF inputs was also tested using a minimal-stimulation protocol. Pharmacologically isolated eEPSCs_AMPA_ were elicited by single pulses of minimum strength delivered at a frequency of 0.2 Hz via the VLRF stimulation electrode. ‘‘Minimal’’ stimulation was defined as a percentage of eEPSCs_AMPA_ failures in the range between 30% and 40%. The detection threshold was set at 3 times the root mean squares noise (threshold 9 pA). Evoked responses below than the threshold level were counted as failures.

For paired pulse facilitation, VLRF was stimulated with pairs of electrical pulses with inter-pulse intervals ranging from 25 to 200 ms in 25 ms steps delivered with 30 s delay between pairs. At least 10 individual responses were averaged at each interval. The repetitive stimulation protocol consisted of a train of 20 pulses at 40 Hz applied to the VLRF. For averaging, 5 to 10 trains were applied, with 60 s intervals between successive trains. The peak amplitude of the individual response throughout the train was determined from the average trace. Estimates of facilitation were expressed relative to the amplitude of first response in the train.

***Unitary extracellular recordings of HMNs* *in the adult rat***

Adult animals (250-300 gr) were prepared for extracellular recordings as reported previously [15,16]. Anesthetized rats (1.5-3% isoflurane mixed with 100% O_2_) received intramuscular injection of atropine (0.2 mg/kg) and dexamethasone sodium phosphate (0.8 mg⁄kg). Teflon-isolated silver bipolar electrodes were fixed around the right XIIth nerves. Electrodes were electrically isolated from neighboring tissue with Vaseline jelly and parafilm. Tracheotomized, vagotomized and decerebrated animals were paralyzed with gallamine triethiodide (20 mg/kg, i.v., initially; 4 mg/kg, i.v., as needed) and mechanically ventilated. Expired CO_2_ and O_2_ were monitored continuously (Eliza duo; Gambro Engström, Bromma, Sweden) and the end-tidal CO_2_ was kept at 4.8-5.2% along the recording session. Rectal temperature (37 ± 1ºC) was continuously monitored.

Three-barreled, microfilament-filled glass pipettes, pulled and broken to a diameter of 5-7 μm, were used for single-unit recording and iontophoresis. The recording barrel (1-3 MΩ) was filled with 3 M NaCl. A second barrel was filled with one of the LPA_1/3_ specific antagonists VPC 32179, VPC 32183 or Ki16425 or alternatively with the vehicle (10% DMSO at pH 8.0) solution. Individual neuronal current response curves to drugs/vehicle were determined by applying increasing currents (-20 to -140 nA, 20 nA steps, 30 s duration) through the drug barrels using the Neurophore BH-2 system (Harvard Apparatus). Retaining currents of +5 nA were applied between steps to minimize undesired drug flow from the barrel.

Multibarrel glass micropipettes were placed under visual guidance and advanced through the brainstem into the HN. The correct position of the micropipette was confirmed by recording the characteristic inspiratory pattern and the presence of the antidromic field potential elicited by electrical stimulation of the ipsilateral XIIth nerve. HMNs were identified by their antidromic activation from the XIIth nerve and by the collision test (Fig. 9I) [15]. The electrical signals were amplified and filtered at a bandwidth of 10 Hz-10 kHz for display and digitization purposes.

Only inspiratory HMNs discharging at basal conditions (end tidal CO_2_ = 4.8-5.2%) were considered in this study. Unitary discharge activity and percentages of expired CO_2_ and O_2_ recordings were amplified, filtered, digitized and stored on computer using the PowerLab/8SP A/D interface (ADInstruments, Castle Hill, Australia) for offline analysis. The unitary mean firing rate (mFR) (spikes/s) in each burst was measured. Bursts were automatically selected using a macro function and parameters were saved in a data pad for subsequent statistical analysis.

***Immunohistochemistry***

Animals were anesthetized with chloral hydrate, injected intraventricularly with heparin, and perfused transcardially with PBS, followed by 4% paraformaldehyde (PFA) in 0.1 M phosphate buffer (PB), pH 7.4, at 4°C. The brains were removed and postfixed for 2 h in the same fixative solution. Brains were cryoprotected by overnight immersion in 30% sucrose in 0.1 M PB at 4°C. Serial coronal sections (30 μm-thick) were obtained from brainstems using a microtome and stored at -20°C in a cryoprotectant solution (glycerol/PBS, 1:1 v/v) [1].

Sections were rinsed in PBS and immersed in 2.5% (w/v) bovine serum albumin, 0.25% (w/v) sodium azide, and 0.1% (v/v) Triton X-100 in PBS for 30 min, followed by overnight incubation at 4°C with different combinations of up to 3 primary antibodies. Polyclonal primary antibodies used in this study were anti-VGLUT2 (1:2000) developed in guinea pig, anti-VGAT (1:2000; Millipore Bioscience Research Reagents), anti-gephyrin (1:50; Santa Cruz Biotechnology) and anti-Munc13-1 (1:1000; Synaptic systems, Goettingen, Germany) developed in rabbit; anti-LPA_1_ (1:50; Santa Cruz Biotechnology) developed in goat, and anti-SMI32 (1:8000; Covance) developed in mouse. Subsequently, after rinsing with PBS the tissue was incubated for 2 h at room temperature with the secondary antibodies, developed in donkey: anti-guinea pig, anti-goat, anti-mouse, or anti-rabbit IgGs labeled with the cyanine 2, 3, or 5 (Cy2, Cy3, Cy5) (1:200; Jackson ImmunoResearch Laboratories). Finally, sections were washed with PBS and mounted on slides with a solution of propyl gallate (0.1 mM in PBS/glycerol, 1:9 v/v). Omission of the primary antibodies resulted in no detectable staining.

Slides were analyzed using a Leica confocal microscope for fluorescence. Images were acquired in a z-plane where optimal antibodies diffusion was reached. Synaptic inputs of motoneurons perikarya were analyzed in a z-plane containing the nucleus as previously reported [1,2,12,16]. The pinhole opening was 1 Airy unit. As established previously [17], only puncta reporting an area of > 0.04 μm^2^ were taken as specific.

Isolated SMNs were plated in four-well tissue culture dishes (Nunc, Thermo Fisher Scientific) for immunohistochemistry (70,000 cells per well). The cultures of SMNs were fixed in 4% paraformaldehyde (15 min at room temperature) and 100% methanol (10 min at 4ºC), incubated overnight at 4°C with a polyclonal anti-SMI32 (1:8000), and further incubated with an anti-mouse secondary antibody conjugated with Cy2 for 1 h at room temperature protected from light. Nuclear staining was obtained with 0.1 μg/µl DAPI for 1 hour at room temperature. Micrographs were obtained using a BX60 Olympus (Tokyo, Japan) epifluorescence microscope.

For GABA_A_γ_2_ and gephyrin immunocytochemistry, SMNs at DIV 6 were incubated for 40 min with aCSF alone (control), 30 min with aCSF plus 10 min with s-LPA (40 μM) dissolved in aCSF, 40 min with dynasore (80 μM) or 30 min with dynasore plus 10 min with dynasore+s-LPA. Cells were immediately fixed as above. For immunolabeling mouse monoclonal anti-gephyrin (1:700; Synaptic Systems) and rabbit polyclonal anti-GABA_A_γ_2_ (1:500; Synaptic Systems) primary antibodies were used.

Primary antibodies were added sequentially; first cells were incubated for 40 min (RT) with the anti-GABA_A_γ_2_ prior to permeabilization to label GABA_A_Rs present at the plasma membrane, and subsequently, SMNs were incubated with anti-gephyrin for 1 hr (RT) after permeabilization (as above). Incubation with appropriate secondary antibodies was performed as above. Isolated cells immunolabeled for GABA_A_γ_2_ and gephyrin were chosen and acquired as previously reported [18].

Images for quantification were acquired under a 60X oil-immersion objective by a cooled CCD camera. All images for each antibody were acquired with the same exposure time. Quantifications of GABA_A_γ_2_- or gephyrin-ir clusters were performed using ImageJ 1.48v software from the National Institutes of Health. Images for quantification were flattened and background-filtered to enhance cluster outlines and a user-defined intensity threshold was applied to select clusters as previously described [18].

***Electron microscopy***

Brainstem slices (300 μm-thick), obtained as above, were incubated for 10 min (~22°C) with aCSF alone, 0.2% DMSO (vehicle) or with various drug treatments. Afterward, the tissue was immediately fixed by 45 min immersion in 3.5% glutaraldehyde in 0.1 M PB, pH 7.4, at 36ºC. Slices were then left 30 min at room temperature before storing 20 h at 4ºC. Finally the slices were rinsed with PB and stored in PB with 0.05% sodium azide at 4°C until electron microscopy processing. Sections were postfixed with 2% osmium tetroxide in PB for 90 min. Subsequently, the slices were rinsed with distilled water, dehydrated in graded series of ethanol, and stained for 2 h 30 min in 2% uranyl acetate in 70% ethanol at 4°C. Afterward, sections were dehydrated, washed with propylene oxide (2 x 10 min), embedded in Araldite resin, and left to polymerize for at least 72 h at 70°C [1,2]. Ultrathin sections (70-80 nm thick) obtained with an ultramicrotome (LeicaEMUC6) were analyzed under a FEI Tecnai Spirit electron microscope at high magnification (43,000x) attached to a digital camera (Morada Preview). Captured images were analyzed off-line using the ImageJ free software. Only boutons, contacting with motoneurons at the level of the nucleolus, evidencing at least an a.z. were included in this study [2]. Quantification was performed by investigators who were blind to the experimental conditions.

***siRNA-mediated silencing of* lpa1**

Neonatal rats (P4) were anesthetized (1.5-3% isoflurane in 100% O_2_) and placed in a Kopf stereotaxic instrument. The needle of a microsyringe (5 μl, Hamilton Company, Tokyo, Japan) crossed skull through the middle point of the interparieto-occipital suture and was advanced parallel to occipital bone up to the fourth ventricle (Fig. 3D). The final position of the needle ending was confirmed visually by means of a surgical microscope. The animals then received an acute injection of siRNA*_lpa1_* or non-targeting siRNA (cRNA) (2 μg/rat; Accell, Dharmacon Inc., Lafayette, CO) in 2 μl of RNase-free PBS at a rate of 0.5 μl/min. The target sequence for the siRNA*_lpa1_* was UCAUUGUGCUUGGUGCCUU. Some animals were infused with 2 μl of RNase-free PBS (vehicle) as an additional control. After the injection, the needle was left in place for 5 min, then slowly removed. Brainstems of treated rats were quickly extracted at P6, P7 and P8 for *q*RT-PCR analyses. Rostral and caudal portions of the brainstem region containing the HN, obtained from P6 rats used for electrophysiological studies, were additionally processed for *q*RT-PCR. Only recorded HMNs from animals that reported at least 80% knockdown of *lpa1* were included in this study.

Primary cultures of SMNs were established as above. After 24 h, cells were triple-washed with PBS to remove cell debris. In separate tubes, 2.5 μl of either cRNA or siRNA*_lpa1_* (each 100 μM;) were mixed with 200 μl Accell siRNA Delivery Media supplemented with B27 (2% v/v) and a cocktail of recombinant neurotrophic factors as described above. After addition of siRNA oligos, SMNs were incubated for 72 h at 37ºC according to the manufacturer's protocol. Subsequently, transfection medium was removed and cells were cultured in Neurobasal complete medium as described above. Cells were then collected for *q*RT-PCR analyses or were used for electrophysiological studies.

***Quantitative real-time reverse transcriptase PCR (*q*RT-PCR)***

Total RNA was extracted from the HN or cultured SMNs (100,000 cells per well) using TRIzol (BioLine). To reduce DNA contamination, samples were additionally treated with the RNase-free DNase set according to the manufacturer (Qiagen). The concentration and purity of RNA samples were determined by spectrophotometry at 260 and 280 nm, and 0.5 μg of RNA was used for cDNA synthesis with iScript cDNA synthesis (Bio-Rad). *q*RT-PCR was performed using iQ SYBR Green Supermix (Bio-Rad) with the MiniOpticon real-time PCR detection system (Bio-Rad). The PCR primers were as indicated in S2 Table. In all cases, the validity of amplification was confirmed by the presence of a single peak in the melting temperature analysis and linear amplification with increasing number of PCR cycles. Control samples obtained by omission of RT were used to detect potential contaminations with genomic DNA. Amplification was absent in these RT(-)-controls for all the primers.

***Western Blotting***

Brainstem slices from neonatal rat pups (P6-P9) were obtained as described above and incubated for 10 min in carbogen-bubbled aCSF alone (control) or supplemented with 0.2% DMSO (vehicle) or drug treatments. Next, slices were immediately transferred to ice-cold (~4°C) aCSF supplemented with a protease inhibitor cocktail for mammalian cell lysate (Sigma-Aldrich) and a phosphatase inhibitor cocktail (Pierce), where microdissection of HNs was performed. Using a 1 ml syringe, microdissected nuclei were homogenized in lysis buffer [50 mM Tris/HCl, pH 7.4, 1% (v/v) Triton X-100, 0.5% (w/v) sodium deoxycholate] supplemented with protease and phosphatase inhibitor cocktails. At 10 min after treatment with specific drugs or vehicles, NSC34 cells or SMNs (100,000 cells per well) were rinsed with ice-cold PBS and immediately transferred to the lysis buffer with protease and phosphatase inhibitors. For NSC-34 cell lysis, a commercial lysis buffer (Cell Signaling Technology) was used; lysates were centrifuged (1500 x g for 5 min) at 4°C. The supernatants were collected and their protein concentrations were determined by Bradford-protein assay (BioRad). SMNs were lysed in buffer TRIS 125 mM, pH 6.8, 2% SDS and boiled at 100ºC for 5 min. Protein concentration was determined using a micro BCA protein assay kit (Thermo Scientific).

Membrane and cytosol fractionation was performed as reported previously with minor modifications [19,20]. Briefly, SMNs or NSC34s were rapidly homogenized in 0.32 M cold sucrose containing 1 mM HEPES, 1 mM MgCl_2_, 1 mM NaHCO_3_, and 0.1 mM PMSF, pH 7.4 in the presence of a complete set of proteases and phosphatase inhibitors. The homogenized tissue was centrifuged at 1500 x g for 10 min. The resulting supernatant was centrifuged at 10,000 x g for 15 min (4ºC) to obtain the cytosol fraction (supernatant) and the crude membrane fraction (pellet). The pellet was resuspended and incubated for 1 hour in ice-cold (~4°C) loading buffer.

An equal amount of protein per sample was separated on Tris glycine gels using 1x Tris-glycine SDS running buffer. The separated proteins were electrophoretically transferred onto a PVDF membrane (Amersham), which was then blocked with BSA 5%, 0.1% Tween for 1 hr at room temperature. Membranes were blotted with a specific antibody against GABA_A_γ_2_ developed in goat (1:900; Santa Cruz Biotechnology) and/or p^Ser327^GABA_A_γ_2_ developed in rabbit (1:1000; Abcam), LPA_1_ (1:200) developed in goat, p-MLC and MLC developed in goat and rabbit, respectively (1:200), or RhoA developed in mouse (1:200; Santa Cruz Biotechnology). Membranes were also probed with anti-α_1_-tubulin (1:100,000; Sigma-Aldrich) or anti-β-actin (1:2,500,000; Sigma-Aldrich) antibodies as control for the total amount of protein contained in each well. Analysis was performed using the ImageJ 1.48v software.

***ROCK and CaN activity assays***

Brainstem slices or cells were obtained and incubated as indicated above. The HNs were microdissected and immediately immersed in 20 mM Tris pH 8, 250 mM sucrose (70 µl). The tissue was then homogenized using an insulin syringe, and subsequently centrifuged at 1,500g for 5 min to remove the nuclei. Supernatant was used to measure ROCK activity with the 96-well ROCK Activity Assay Kit (Cell Biolabs) according to the manufacturer’s instructions. To assure that the signal measured was due specifically to ROCK activity, we used as negative controls our samples plus the specific ROCK inhibitor H1152 (100 µM). For this assay, NSC34 cells were allowed to differentiate for 9 days in 6-well tissue culture dish (Cultek) and then they were treated with s-LPA for 10 min. Untreated and treated NSC34 cells were washed with PBS and scraped into the buffer described above. The following procedures were identical to those for HNs.

Primary cultures of SMNs were homogenized in 50 mM Tris, 1 mM DTT, 100 µM EDTA, 100 µM EGTA, 0.2% NP-40 lysis buffer, at pH 7.5. The homogenized cells were boiled for 5 min and then CaN activity was measured using the CaN Cellular Activity Assay Kit (Calbiochem) according to manufacturer instructions.

***Cultures of the motoneuron-like cell line NSC34***

NSC34 cells were purchased from CELLutions Biosystems Inc (Toronto, Canada). Undifferentiated NSC34 cells were maintained in high glucose Dulbecco’s modified Eagle’s medium (DMEM), supplemented with 10% (vol/vol) fetal calf serum (FCS), 2 mM L-glutamine and 1% (vol/vol) penicillin/streptomycin. The medium was exchanged every 2 days. To favor their maturation, NSC34 were plated at low density (4000 cells/cm^2^) in 1:1 DMEM plus Ham’s F12 medium supplemented with 1% (vol/vol) FCS, 1% penicillin/streptomycin, and 1% modified Eagle’s medium nonessential amino acid (Sigma-Aldrich), as described previously [21]. After 7-9 days in this medium the differentiated phenotype of these cells was visually verified. Cell culture consumables were from GibcoBRL, except when otherwise specified. Tests for RhoA/ROCK activity and LPA_1_ expression were performed after nine days in differentiating conditions.

***Drugs and treatments***

To isolate different components of synaptic signaling on motoneurons, the following drugs were used: the voltage-gated sodium channels inhibitor tetrodotoxin (TTX, 1 μM), the glycine receptor antagonist strychnine hydrochloride (1 μM), the nicotinic acetylcholine receptor antagonist d-tubocurarine (30 µM), the NMDA receptor antagonist (DL)-APV (50 μM), the GABA_A_ receptor antagonist bicuculline methochloride (10 µM) and the AMPA receptor antagonist NBQX (20 μM), all of them purchased from Tocris Cookson (Bristol, UK).

Either alone or in different combinations, the following drugs were used along this study: 1-oleoyl-2-hydroxy-*sn*-glycero-3-phosphate (18:1 LPA; 1 nM to 20 μM; Sigma-Aldrich), 1-stearoyl-2-hydroxy-*sn*-glycero-3-phosphate (18:0, s-LPA; 40 μM), the LPA_3_ specific agonist OMPT (1 μM), the LPA_1,3_ specific antagonists VPC 32179 (1 μM for *in vitro* and 0.5 mM for *in vivo* procedures), VPC 32183 (1 μM for *in vitro* or 1 mM for *in vivo* procedures; Avanti Polar Lipids Inc, Alabaster, AL) or Ki16425 (0.04 or 0.4 μM for *in vitro* or 2 mM for *in vivo* procedures; Cayman Chemical Company, Ann Arbor, Michigan), the specific inhibitor of ROCK (*S*)-(+)-2-methyl-1-[(4-methyl-5-isoquinolinyl)sulfonyl]-hexahydro-1H-1,4-diazepine dihydrochloride (H1152; 20 or 100 μM; Tocris Bioscience), the MLCK inhibitor 1-(5-Iodonaphthalene-1-sulfonyl)-1H-hexahydro-1,4-diazepine hydrochloride (ML-7; 10 μM; Sigma-Aldrich), the CaN autoinhibitory peptide (Cap; 12.5 or 50 μM; Calbiochem), the specific inhibitor of phospholipase C (PLC) U73122 (1 μM; Tocris) and its inactive analog U73343 (5 μM; Sigma-Aldrich), the specific inhibitor of G_αi/o_ pertussis toxin (PTX; 100 ng/ml) and its non-catalytic membrane permeable B oligomer of PTX (bPTX; 100 ng/ml; Calbiochem), the specific inhibitor of G_αq/11_ YM-254890 (1 μM; a generous gift from Taiho Pharmaceutical Co, Tsukuba, Ibaraki, Japan), and the dynamin inhibitor dynasore (80 μM; Sigma-Aldrich). All products were water soluble except Ki16425, ML-7, U73122, U73343, and dynasore which were dissolved in 0.2% DMSO for *in vitro* procedures.

All studies *in vitro* were performed at least 8 to 10 min after addition of drugs to the bath solution except for Cap and dynasore, which were added to the patch pipette solution (Cap) or to the bath for 30 min before recordings or lysates performance, and PTX and bPTX, which were added to the bath 2 h before recordings.

All drugs were prepared just before the experiment from stock solutions maintained at -20°C and applied to the recording bath either alone, in combination, or in specific sequences by switching the perfusion line manually. In general, the protocol used for studying the effects of the drugs was as follows: all motoneurons were initially perfused with normal aCSF to obtain baseline control data. After baseline pre-exposure recording, the specimen was superfused for 10 min with aCSF containing a given drug before voltage responses were acquired again. In some experiments, a second drug was subsequently added to the perfusate and its effects tested 10 min later. Finally, a last round of acquisition was taken after a 10 min washout with drug-free aCSF. Schematic diagrams illustrating the timing of drugs addition to the bath solution and PSCs recordings are shown in Fig. 1M and 3A.

***Statistics and data analysis***

Data are expressed as the mean ± standard error of the mean (SEM). The number of analyzed specimens per experimental condition is indicated in figure legends or in the results section. Data were obtained from at least 3 animals per experimental condition. In ROCK activity, western blotting and *q*RT-PCR experiments, each individual assay was performed using tissue samples collected from at least 6 animals per experimental condition. Quantitative data from ROCK and CaN activity assays, western blot and qRT-PCR represent the average of at least 3 independent experiments. Applied statistical tests per experimental condition are indicated in figure legends or in results. *Post hoc* Holm Sidak or Dunn tests were applied for ANOVA for repeated measures or on Ranks, respectively. In all cases, the minimum significance level was set at *p* < 0.05.

**REFERENCES**

1. Sunico CR, Gonzalez-Forero D, Dominguez G, Garcia-Verdugo JM, Moreno-Lopez B (2010) Nitric oxide induces pathological synapse loss by a protein kinase G-, Rho kinase-dependent mechanism preceded by myosin light chain phosphorylation. J Neurosci 30: 973-984.

2. Gonzalez-Forero D, Montero F, Garcia-Morales V, Dominguez G, Gomez-Perez L, et al. (2012) Endogenous Rho-kinase signaling maintains synaptic strength by stabilizing the size of the readily releasable pool of synaptic vesicles. J Neurosci 32: 68-84.

3. Gonzalez-Forero D, Portillo F, Sunico CR, Moreno-Lopez B (2004) Nerve injury reduces responses of hypoglossal motoneurones to baseline and chemoreceptor-modulated inspiratory drive in the adult rat. Journal of Physiology-London 557: 991-1011.

4. Rekling JC, Feldman JL (1998) PreBotzinger complex and pacemaker neurons: hypothesized site and kernel for respiratory rhythm generation. Annu Rev Physiol 60: 385-405.

5. Turrigiano GG, Leslie KR, Desai NS, Rutherford LC, Nelson SB (1998) Activity-dependent scaling of quantal amplitude in neocortical neurons. Nature 391: 892-896.

6. Carroll RC, Lissin DV, von Zastrow M, Nicoll RA, Malenka RC (1999) Rapid redistribution of glutamate receptors contributes to long-term depression in hippocampal cultures. Nat Neurosci 2: 454-460.

7. Sriwai W, Zhou H, Murthy KS (2008) G(q)-dependent signalling by the lysophosphatidic acid receptor LPA(3) in gastric smooth muscle: reciprocal regulation of MYPT1 phosphorylation by Rho kinase and cAMP-independent PKA. Biochem J 411: 543-551.

8. Ohta H, Sato K, Murata N, Damirin A, Malchinkhuu E, et al. (2003) Ki16425, a subtype-selective antagonist for EDG-family lysophosphatidic acid receptors. Mol Pharmacol 64: 994-1005.

9. Nakajima H, Kubo T, Semi Y, Itakura M, Kuwamura M, et al. (2012) A rapid, targeted, neuron-selective, in vivo knockdown following a single intracerebroventricular injection of a novel chemically modified siRNA in the adult rat brain. J Biotechnol 157: 326-333.

10. Akhtar S, Benter IF (2007) Nonviral delivery of synthetic siRNAs in vivo. J Clin Invest 117: 3623-3632.

11. Choi JW, Chun J (2013) Lysophospholipids and their receptors in the central nervous system. Biochim Biophys Acta 1831: 20-32.

12. Sunico CR, Dominguez G, Garcia-Verdugo JM, Osta R, Montero F, et al. (2011) Reduction in the motoneuron inhibitory/excitatory synaptic ratio in an early-symptomatic mouse model of amyotrophic lateral sclerosis. Brain Pathol 21: 1-15.

13. Gonzalez-Forero D, Portillo F, Gomez L, Montero F, Kasparov S, et al. (2007) Inhibition of resting potassium conductances by long-term activation of the NO/cGMP/protein kinase G pathway: A new mechanism regulating neuronal excitability. Journal of Neuroscience 27: 6302-6312.

14. Gou-Fabregas M, Garcera A, Mincheva S, Perez-Garcia MJ, Comella JX, et al. (2009) Specific vulnerability of mouse spinal cord motoneurons to membrane depolarization. J Neurochem 110: 1842-1854.

15. Montero F, Portillo F, Gonzalez-Forero D, Moreno-Lopez B (2008) The nitric oxide/cyclic guanosine monophosphate pathway modulates the inspiratory-related activity of hypoglossal motoneurons in the adult rat. European Journal of Neuroscience 28: 107-116.

16. Sunico CR, Portillo F, Gonzalez-Forero D, Moreno-Lopez B (2005) Nitric oxide-directed synaptic remodeling in the adult mammal CNS. Journal of Neuroscience 25: 1448-1458.

17. Trimbuch T, Beed P, Vogt J, Schuchmann S, Maier N, et al. (2009) Synaptic PRG-1 modulates excitatory transmission via lipid phosphate-mediated signaling. Cell 138: 1222-1235.

18. Bannai H, Levi S, Schweizer C, Inoue T, Launey T, et al. (2009) Activity-dependent tuning of inhibitory neurotransmission based on GABAAR diffusion dynamics. Neuron 62: 670-682.

19. Gardoni F, Picconi B, Ghiglieri V, Polli F, Bagetta V, et al. (2006) A critical interaction between NR2B and MAGUK in L-DOPA induced dyskinesia. J Neurosci 26: 2914-2922.

20. Goebel-Goody SM, Davies KD, Alvestad Linger RM, Freund RK, Browning MD (2009) Phospho-regulation of synaptic and extrasynaptic N-methyl-d-aspartate receptors in adult hippocampal slices. Neuroscience 158: 1446-1459.

21. Eggett CJ, Crosier S, Manning P, Cookson MR, Menzies FM, et al. (2000) Development and characterisation of a glutamate-sensitive motor neurone cell line. J Neurochem 74: 1895-1902.
